# Acknowledgment to Reviewers of *Cancers* in 2020

**DOI:** 10.3390/cancers13030382

**Published:** 2021-01-21

**Authors:** Cancers Editorial Office

**Affiliations:** MDPI AG, St. Alban-Anlage 66, 4052 Basel, Switzerland

Peer review is the driving force of journal development, and reviewers are gatekeepers who ensure that *Cancers* maintains its standards for the high quality of its published papers. Thanks to the cooperation of our reviewers, in 2020, the median time to first decision was 16.2 days and the median time to publication was 36 days. The editors would like to express their sincere gratitude to the following reviewers for their precious time and dedication, regardless of whether the papers were finally published:
Abad, CatalinaLindqvist, Ebba KAbba, Mohammed L.Lindsay, HowardAbbondanza, CiroLindström, Mikael S.Abd Elmageed, Zakaria Y.Link, AndreasAbdel Fattah, Abdel RahmanLink, WolfgangAbdel-Daim, MohamedLinnebacher, MichaelAbdelrahim, MaenLinnenbringer, ErinAbdel-Rahman, Mohamed H.Linxweiler, MaximilianAbe, HiroyukiLiobikas, JuliusAbe, MasahiroLion, ThomasAbe, TakashigeLiontos, MichalisAbe, TatsuyaLiou, Geou-YarhAbedi Ardekani, BehnoushLiou, Yih-CherngAberger, FritzLipina, RadimAbeykoon, JithmaLipkowitz, StanleyAbian, OlgaLippert, EricAbiko, KaoruLis, AnnetteAbken, HinrichLishner, MichaelAbou-Alfa, GhassanLisi, LuciaAbounader, RogerLiss, AndrewAbramowicz, AgataLita, AdrianAbruzzese, ElisabettaLithgow, BrianAbu Eid, RashaLittle, Andrew C.Accardi, LuisaLittle, JulianAcedo, PilarLitvinov, IvanAcharyya, SwarnaliLiu, Chao lienAcheva, AnnaLiu, Chung-JungAchimas, PatriciuLiu, Chun-YuAchreja, AbhinavLiu, DongfangAcker, GülizLiu, DongxuAckermann, SandraLiu, FengAcquaviva, ClaireLiu, GuigenAdamczewski, ZbigniewLiu, JiaAdamek, AgnieszkaLiu, Jui-MingAdams, Mark N.Liu, Ko-JiunnAdams, Sarah F.Liu, LihuaAdamson, David CoryLiu, LinboAdeberg, SebastianLiu, Ming-CheAdejare, AdeboyeLiu, MoAdekoya, Timothy O.Liu, Pei Y.Adhikari, ManishLiu, PengdaAdriaens, CarmenLiu, QunAdunyah, Samuel E.Liu, RuiwuAeddula, Narothama ReddyLiu, ShirongAfaq, FarrukhLiu, TaoAfelik, SolomonLiu, WeiAffolter, AnnetteLiu, Wei-HsiuAfonso, JulietaLiu, Wei-MinAgarwal, Atin A.Liu, XiangleiAgarwal, NeerajLiu, XiaoyingAgarwal, PoonamLiu, XiyongAgarwal, SaurabhLiu, XuefengAgarwal, SumitLiu, YangAger, AnnLiu, Yi-WenAgerbæk, Mette Ø.Liu, YuAglietta, MassimoLiu, YuanAgnieszka, GizakLiu, Yu-ChangAgnoletto, ChiaraLiu, Yu-PengAgodi, AntonellaLiu, ZhenqiuAgostini, FrancescoLiu, Zhi-jie (Jason)Agoulnik, AlexanderLiu, ZhongweiAgresta, FerdinandoLiu, ZiqingAguilera, ToddLiu-Smith, FengAhadova, AyselLiverani, ElisabettaAhamed, MuneerLlacer, CasildaAhlman, Mark A.Lleo De Nalda, AnaAhluwalia, AmritaLo Presti, ElenaAhmad, AamirLo Re, DanieleAhmad, FahimLo Sardo, FedericaAhmadian, M. RezaLo, Hui-WenAhmed, Atif AliLo, Jeng-FanAhmed, Atique ULo, Kwok WaiAhmed, KhalilLo, Leu-WeiAhmed, Shafiq ULo, SimonAhn, Kook-jinLo, Su HaoAhn, Myung-JuLo, U. GingAhn, Yong ChanLobo, JoaoAi, YongLockman, Paul R.Aiba, HisakiLockridge, OksanaAigner, AchimLoda, MassimoAigner, ClemensLoffroy, RomaricAikata, HiroshiLohmann, DietmarAiraksinen, Anu J.Löhr, MatthiasAjioka, ItsukiLoizidou, MarilenaAkamatsu, ShusukeLok, Chun NamAkbari, Mohamad R.Lokeshwar, BalakrishnaAkbari, OmidLokman, NoorAkhavan-Sigari, RezaLo-Kong, ChanAkimoto, KazunoriLokshin, Anna E.Akıncılar, Semih CanLolkema, Martijn P.Akkari, YassmineLollo, GiovannaAkula, ShawLomas, OliverAl-Abdulla, RubaLombardi, AngelaAlahari, SureshLombardi, GiuseppeAlaibac, MauroLombardi, TommasoAlaimo, AlessandroLomberk, GwenAlam, Mohammad AbrarLonardo, AmedeoAlam, Mohammad ParvezLonardo, EnzaAlam, SaminaLondin, EricAlama, AngelaLondon, NyallAlanna Kulchak, RahmLonetti, AnnalisaAlao, John PatrickLong, DavidAlarcón Fortepiani, María LourdesLong, MeixiaoAlavi, AbassLongo, AntonioAlbano, DomenicoLongo, FrancescoAlbeituni, SabrinLongy, MichelAlbers, PeterLoog, MartAlbert, RekaLooijenga, LeendertAlbert, StewartLoomans-Kropp, HolliAlberti, SaverioLoosen, Sven H.Alberto, ZaniboniLopatina, TatianaAlbuquerque, KevinLopci, EgestaAlcantara, MarionLopes, Jose ManuelAldieri, ElisabettaLópez, CristinaAldinucci, DonatellaLõpez, FernandoAldrich, Melissa B.López, José I.Alegret, NuriaLopez, Jose IgnacioAlemany, RamónLopez-Bergami, PabloAleš, RozmanLópez-Camarillo, CésarAlessandro, FanzaniLópez-Guerrero, José AntonioAlessio, AnnovazziLópez-Hernández, Luz BereniceAlexander, David B.Lopez-Jornet, PiaAlexander, H. DenisLópez-López, ElixabetAlexandraki, KrystalleniaLopez-Martin, Jose A.Alexandrova, ElenaLöpez-Montero, IvanAlexa-Stratulat, TeodoraLópez-Nieva, PilarAlfieri, Roberta R.López-Urrutia, EduardoAlfonso, PicóLorenzo Bermejo, JustoAl-Hilal, Taslim AhmedLorenzo, SempereAli, AimanLorusso, VitoAlic, LejlaLosi, LorenaAlieva, MariaLoskog, AngelicaAlifano, MarcoLosurdo, GiuseppeAlimandi, MaurizioLötsch, DanielaAliotta, EricLotti, FrancescoAljabali, AlaaLou, EmilAlkasalias, TwanaLoughan, Ashlee R.Alkhateeb, AbedalrhmanLoughlin, Kevin R.Allan, AlisonLourdusamy, AnbarasuAllan, John N.Loureiro, Felipe Dos SantosAllard-Chamard, HuguesLovejoy, DavidAllavena, PaolaLow, Siew-KeeAllegra, EugeniaLowery, MaeveAllegrucci, CinziaLoza-Coll, Mariano AndresAllen, BryanLozano, ElisaAllen-Petersen, BrittanyLu, Jeng-WeiAllison, SimonLu, Jyh-FengAllouche, MichèleLu, Li-FanAlmangush, AlhadiLu, LingengAlmeida, Afonso Rocha MartinsLu, Long-ShengAlmeida, Luciana OliveiraLu, Mei-ChinAlmeida, RaquelLu, MinAlmeida, Rodrigo DeLu, MingyangAlmont, ThierryLu, ShellyAlmstrup, KristianLu, XinAlongi, PierpaoloLu, YunxiaAlonso, JavierLubecka-Pietruszewska, K.Alonso, SergioLuca, FalzoneAlonso, VeronicaLuca, RossellaAlonso-Colmenar, Luis M.Lucacchini, AntonioAlonso-Moreno, CarlosLucarelli, GiuseppeAlpaugh, KatherineLucarini, ValeriaAlpini, GianfrancoLucas, ChristopherAlpy, FabienLucas, EmmaAlsaab, HashemLucchetti, DonatellaAlsadeq, AmeeraLucchi, MarcoAl-Samadi, AhmedLuchinat, ClaudioAlshareef, AbdulraheemLuchini, ClaudioAltieri, BarbaraLucia, FrançoisAltieri, FabioLucibello, MariaAltman, Brian J.Lucotti, SerenaAltomonte, JenniferLuddy, Kimberly A.Altucci, LuciaLudlow, Andrew T.Alugubelly, NavathaLudovico, PaulaAlvarez-Dominguez, CarmenLudovini, ViennaÁlvarez-Mercado, Ana I.Ludvikova, MarieAlvarez-Vallina, LuisLudwig, Dale L.Alves, CelsoLudwig, MichaelAlvisi, GualtieroLudwig-Słomczyńska, Agnieszka H.Alyamani, MohammadLuglio, GaetanoAlzrigat, MohammadLui, Vivian Wai YanAlzubaidi, LaithLukas, Rimas VÅman, PierreLuksic, IvicaAmano, JunkoLukyanov, Konstantin A.Amaral, CristinaLuminari, StefanoAmaraL, TeresaLuna, AntonioAmaro, AdrianoLunardi, AndreaAmato, RosarioLund, ThomasAmbriovich Ristov, AndrejaLunde Husebø, Anne MarieAmbro, LubosLundstrom, KennethAmbros, PeterLunghi, MoniaAmbrosini, GraziaLunov, OlegAmbrosino, ConcettaLuo, Chi-WenAmelio, Antonio L.Luo, DanmengAmerio, PaoloLuo, JianAmicone, LauraLuo, Ji-DungAmini, AryaLuo, MingAminin, DmitryLuo, Sheng-DeanAmit, MoranLuo, XuAmmendola, MicheleLupiáñez, José A.Ammirati, MarioLupu, MihaiAmodio, NicolaLüscher, BernhardAmornyotin, SomchaiLuscinskas, Francis William (Bill)Ampollini, LucaŁuszczki, JarogniewAmrani, AbdelazizLuwor, RodneyAmrutkar, ManojLuxton, G. W. GantAnand, KartikLynam-Lennon, NiamhAnand, SanjayLyng, FionaAnania, Maria ChiaraLynge, ElsebethAnaniev, JulianLyons, Kathleen D.Anastasiou, Olympia E.Lyons, ShawnAndani, SonaliLyons, TraciAndera, LadislavLyons, Traci R.Anděra, LadislavLyratzopoulos, GeorgiosAnderson, Alyce J. M.M’kacher, RadhiaAnderson, Mary AnnMa, CynthiaAnderson, PeterMa, ShuanggeAnderson, Rhona S.Ma, StephanieAnderson, RobinMa, YingAndersson, MattiasMaass, KendraAndersson, YvetteMaayah, ZaidAndl, Claudia D.Maccallini, CristinaAndo, KoichiMaccarinelli, FedericaAndo, KojiMaccaroni, ElenaAndón, Fernando TorresMacchi, BeatriceAndradas, ClaraMacek Jílková, ZuzanaAndré, NathalieMacerola, ElisabettaAndréani, JulienMach, Robert HAndreassen, Paul R.Machairiotis, NikolaosAndreetti, ClaudioMachala, ZdenkoAndreucci, ElenaMachicado, Jorge D.Andreuzzi, EvaMacias, Rocio I. RodriguezAndrews, LesleyMacias-Gonzalez, ManuelAndrews, MilesMaciejczyk, MateuszAndrysic, ZdenekMaciejewski, RyszardAnemona, LuciaMacip, SalvadorAngelousi, AnnaMacLeod, NicholasAngelova, AssiaMacNeill, AmyAngelova, MihaelaMacPherson, DavidAngelucci, AdrianoMacRobert, SandyAngelucci, EmanueleMacurek, LiborAngioni, RobertaMadak-Erdogan, ZeynepAngrand, Pierre-OlivierMadamsetty, Vijay SagarAngrisano, TizianaMadariaga, AinhoaAniello, FrancescoMadden, Leigh A.Anikin, VladimirMadka, VenkateshwarAnken, RalfMadl, TobiasAnnaratone, LauraMadureira, Patrícia A.Annunziata, Christina MessineoMaes, KenAnsa, BenjaminMaestro, RobertaAnsems, MarleenMaffini, FaustoAnthony, DonaldMaffioli, MargheritaAntic, TatjanaMagge, Deepa RAntón, AntonioMagge, RajivAntonarakis, EmmanuelMaggi, FabrizioAntonelli, AlessandroMaggi, Leonard B.Antonioli, ManuelaMaggialetti, NicolaAntonios, GiakountisMaggiolini, MarcelloAntwi, Samuel O.Magi-Galluzzi, CristinaAnvari, BahmanMaglione, ManuelAnwer, FaizMagnelli, LuciaAnyfantakis, Dimitrios I.Magni, ElenaAnzenbacherova, EvaMagnoni, FrancescaAoki, MasahikoMagrassi, LorenzoAoki, MasahiroMahadevan, DarukaAparicio, ThomasMahapatra, SidharthApartsin, EvgenyMahipal, AmitApcher, SébastienMahmoudi, TokamehApostolopoulos, VassoMahtouk, KarèneAppetecchia, MarialuisaMaier, PatrickAprile, MariannaMainprize, JamesAptsiauri, NataliaMaiorano, EugenioAragonés, JuliánMais, ValerioArai, AyakoMaitra, RadhashreeAran, GemmaMaity, ShuvadeepArasanz Esteban, HugoMajd, NazaninArati, SharmaMajem, BlancaAraúzo-Bravo, MarcosMajkowska-Pilip, AgnieszkaAravalli, Rajagopal N.Majumder, MousumiArcangeli, AnnarosaMajumder, PoulamiArcelli, AlessandraMajumder, SarmilaArcese, WilliamMakena, Monish RamArcucci, AlessandroMakishima, HirokazuArczewska, KatarzynaMakishima, MakotoArena, SabrinaMaksymiuk, Andrew W.Arenas, JuanMakwana, NandiniArend, RebeccaMalaguarnera, MicheleArend, Rebecca C.Malapelle, UmbertoArendt, Lisa M.Malara, AlessandroArevalo, Juan CarlosMalara, NataliaArgentiero, AntonellaMalarkannan, SubramaniamArgyropoulos, KimonMalatino, LorenzoAricò, EleonoraMalavasi, FabioAriel, AmiramMalavaud, BernardArif, TasleemMalejczyk, JacekArima, YoshimiMaleki Vareki, SamanArlt, AlexanderMaleki, ZahraArnaut, Luís GuilhermeMaletzki, ClaudiaArndt, ClaudiaMalfitano, Anna MariaArnesen, ThomasMalgor, RamiroArnold, RudolfMalik, KarimArranz, LorenaMalkiewicz, BartoszArribas, AlbertoMallamaci, RosannaArribas, JoaquínMallio, Carlo AugustoArrogante, ÓscarMallipeddi, SrikrishnanArruga, FrancescaMalliri, AngelikiArshad, NajlaMalorni, LucaArtati, AnnaMalpeli, GiorgioAsa, Sylvia L.Malvezzi, MatteoAsano, NaokiMamdani, HirvaAsanuma, KunihiroMamessier, EmilieAshaie, MaeirahMamonkin, MaksimAshihara, EishiManai, FedericoAshman, Jonathan B.Manapov, FarkhadAshton, JohnManca, AntonellaAshton, Nicholas W.Manca, Maria LetiziaAsim, MohammadMancini, MariangelaAsimakopoulos, AnastasiaMancuso, AndreaAspeslagh, SandrineMancuso, FrancescaAsquith, Christopher R.M.Mancuso, Maria-TeresaAssaf, ChalidMancuso, SalvatriceAssenat, EricMandatori, DomitillaAstbury, StuartMandel, JacobAstolfi, AnnalisaMandic, RobertAstsaturov, IgorManenti, AntonioAtanackovic, DjordjeManes, SantosAtaseven, BeyhanManet, EvelyneAtkins, MardelleManfredi, SylvainAtrash, ShebliMangaonkar, AbhishekAttanoos, Richard LutherMangla, AnkitAttarbaschi, AndisheMango, LucioAuberger, PatrickMangogna, AlessandroAudet-Walsh, ÉtienneMangone, LuciaAudrito, ValentinaMani, SridharAurilio, GaetanoManier, SalomonAurrand-Lions, MichelManin, IvanaAust, StefanieMann, MichaelAustin, RobertManna, SaikatAutelli, RiccardoManne, UpenderAuvinen, AnssiMannelli, GiudittaAu-Yeung, Chilam AuManni, AndreaAvgeris, MargaritisManoury, Beńed́icteAvian, AlexanderManov, IrenaAvila, MatiasMansfield, DavidAvin, VijayMansier, OlivierAvino, PasqualeMansouri, AlirezaAvolio, Alfonso W.Mantamadiotis, TheoAvouac, JeromeMantovani, FiammaAvtanski, DimiterMantovani, StefaniaAwada, AhmadManzia, Tommaso MAwada, GilManzo-Merino, JoaquinAxelsson, RimmaManzotti, GloriaAydar, EbruMarampon, FrancescoAydede, Sema K.Maranchie, JodiAydin, YucelMarć, Małgorzata AnnaAye, IrvingMarcato, PaolaAye, JamieMarcel, VirginieAylett, Christopher H.S.Marcenaro, EmanuelaAylon, YaelMarchal, FrédéricAyonrinde, OyekoyaMarchetti, DarioAyuketang Ayuk, FrancisMarchetti, PhilippeAyuso, José MaríaMarchetti, SandrineAzer, Samy A.Marchica, ValentinaAziz, FaisalMarchini, CristinaAzizi, EbrahimMarchini, SergioAzizian, AzadehMarchioni, MicheleAzodi, MasoudMarcia, MarcoAzuma, MizukiMarconett, Crystal N.Azzalin, ClausMarconi, GiovanniBaar, JosephMarcos Gragera, RafaelBaba, HideoMarcovici, GenoBaba, TsukasaMarcu, LoredanaBaba, YuhMarcus, Schmitt-EgenolfBabak, Maria V.Maréchal, AlexandreBabich, JohnMargiotta, AzzurraBabiker, Hani M.Margulis, BorisBabitsch, BirgitMarheineke, KathrinBabyshkina, NataliaMarí-Alexandre, JosepBacalbașa, NicolaeMarian, BrigitteBaccarani, MicheleMariani-Costantini, RenatoBach, Duc-HiepMariano, CaratozzoloBacher, UlrikeMarichalar Mendia, XabierBachet, Jean-BaptisteMarignol, LaureBacso, ZsoltMarijnen, Corrie A.M.Badiola, IkerMarimpietri, DaniloBado, IgorMarín De Evsikova, CaralinaBadr, ChristianMarin, MercedesBae, Dong-HunMarini, Patricia EstelaBae, Jin-wooMarinko, TanjaBae, Jong-MyonMarino, MariaBae, Yong-SooMario, BignardiBaek, Se-JinMarioni, GinoBaer, MariaMariotto, ElenaBaffy, GyorgyMariuzza, Roy A.Bagci-Onder, TugbaMarjanski, TomaszBagga, PuneetMark, Tomer M.Bagge, Roger OlofssonMarkiewicz, AleksandraBago, Juli R.Markiewicz, AnnaBahal, RamanMarkopoulos, Georgios SBahmad, HishamMarkou, AthinaBahrami, ArmitaMarkovina, StephanieBai, Li-PingMarkowitz, JosephBai, Li-YuanMarkus, Waldeck-WeiermairBai, MingfengMármol, InesBai, Xue-fengMarodon, GillesBaietti, Maria FrancescaMarolleau, Jean-PierreBailey, Charles G.Maroni, PaolaBailly, ClementMarosi, ChristineBaiocchi, Gian LucaMarques, Hugo PintoBaiocchi, MartaMarrazzo, PasqualeBaisi, AlessandroMarrelli, DanieleBaj, JacekMarron, ThomasBakator, MihaljMarrone, AldoBaker, MartinMarrone, GiuseppeBakht, MartinMarsano, René MassimilianoBakker, AkkeMarsh, Deborah J.Bakker, Marije FMarshall, JohnBaklouti, FaouziMartano, MarinaBalajthy, ZoltánMartellucci, StefanoBalan, MurugabaskarMartens-Uzunova, Elena S.Balan, SreeKumarMartial, SoniaBalasubramanian, GaneshMartí-Bonmatí, LuisBalayssac, DavidMartignoni, GuidoBalázs, MargitMartin, AlejandroBalcerczyk, AnetaMartin, Elizabeth C.Baldi, AlfonsoMartin, Francis LBaldini, EnkeMartin, Rebecca K.Baldini, NicolaMartin, SophieBalermpas, PanagiotisMartin, SteggallBalestri, FrancescoMartín-Antonio, BeatrizBalgoma, DavidMartinelli, ChiaraBalic, MarijaMartinelli, FabioBalint, L. BalintMartinelli, GiovanniBallarò, RiccardoMartinelli, MariannaBalleine, RosemaryMartínez Beamonte, RobertoBallesta, AnnabelleMartínez Martínez, EstherBallester, Pedro J.Martínez, AlbertoBalogi, ZsoltMartínez, ConstantinoBalzer, Bonnie L.Martinez, InigoBam, RakeshMartinez, IvanBamdad, CynthiaMartinez, MagalyBand, HamidMartínez-Campa, CarlosBandapalli, Obul ReddyMartínez-Guardado, IsmaelBanerjee, AditiMartinez-Jarreta, BegoñaBanerjee, Hirendra NathMartínez-Laperche, CarolinaBanerjee, KasturiMartinez-Useros, JavierBanerji, VershaMartinho, OlgaBang, Chang SeokMartini, AndreaBang, Yung-JueMartini, ClaudiaBaniahmad, AriaMartini, VeronicaBanito, AnaMartín-Montalvo, AlejandroBanks, LoriMartino, FrancescoBanu, AlexandraMartinotti, SimonaBaptista, Pedro MiguelMartins, Andre F.Barabutis, NektariosMartin-Sabroso, CristinaBaralle, MarcoMartinson, HollyBaran, JaroslawMartín-Vasallo, PabloBaranova, AnchaMartner, AnnaBaranowska-Jurkun, AngelikaMartucci, NicolaBaras, Alexander S.Martucciello, GiuseppeBarasch, AndreiMarty, CarolineBaratto, LuciaMarulli, GiuseppeBarault, LudovicMaruoka, YasuhiroBarauskas, GiedriusMarusiak, Anna A.Barazzuol, LaraMarusyk, AndriyBarbáchano, AntonioMaruyama, IchiroBarbagallo, CristinaMarvaso, GiuliaBarbagallo, DavideMarverti, GaetanoBarbara, BreznikMarwitz, SebastianBarbarino, MarcellaMarzo, TizianoBarbazán, JorgeMasago, KatsuhiroBarberis, MassimoMasai, HisaoBarbieri, FedericaMasaki, TsutomuBarbieri, GiovannaMasalova, Olga V.Barbosa, ArmenioMasaoka, TatsuhiroBarbui, TizianoMaschmeyer, GeorgBarcellos-Hoff, Mary HelenMasciarelli, SilviaBarciszewska, Anna-MariaMascitti, MarcoBardag-Gorce, FawziaMashima, RyuichiBarg, EwaMasi, AnnalisaBarger, CarterMasi, GianlucaBarile, AntonioMaslowski, KendleBařinka, CyrilMassa, AnnamariaBaritaki, StavroulaMassa, ChiaraBariuso, JorgeMassade, LilianeBarlier, AnneMassari, FrancescoBarlow, JacquelineMassimi, MaraBarnaba, VincenzoMassimino, MauraBarnea, Eytan RMassion, PierreBarnett, BradMastrangelo, StefanoBarnhill, RaymundMastronuzzi, AngelaBarok, MárkMastropasqua, MauroBarone, RosarioMasucci, MariaBarosi, GiovanniMasuda, JunkoBarr, MartinMasuda, KiyoshiBarra, FabioMasuda, NorikazuBarra, GiusiMasuda, TakeshiBarragán, IsabelMasui, KentaBarreiro, EstherMasui, ToshihikoBarrero, María JoséMasutomi, KenkichiBarresi, ValeriaMasuzaki, RyotaBarresi, VincenzaMatacchione, GiuliaBarret, MaximilienMatarredona, Esperanza R.Barrios-Rodríguez, RocioMatarrese, PaolaBarritt, GregMatei, DanielaBarroso, MargaridaMateos, María VictoriaBarrott, JaredMaterin, Miguel A.Barrow, AlexanderMatesic, Lydia E.Barrow, Alexander DavidMathew, StephenBarry, Kathryn HughesMathieu, Marie-ChristineBarsyte-Lovejoy, DaliaMathonnet, MurielBartee, EricMatias-Guiu, XavierBartel, KarinMatin, AbdulBartels, StephenMatlawska-Wasowska, KseniaBarth, BrianMato, EugeniaBartholomä, Mark DanielMatos, PauloBartkuhn, MarekMatosevic, SandroBartollino, SilviaMatozaki, TakashiBartsch, Jörg W.Matrana, Marc RyanBartsch, RupertMatrisian, Lynn M.Bartzsch, StefanMatrone, AntonioBarutello, GiuseppinaMatsuba, TakashiBasadonna, GiacomoMatsuda, AtsushiBasak, DebasishMatsuda, YokoBaschant, UlrikeMatsui, YoshiyukiBasilico, CristinaMatsuki, TakahiroBaskaran, SaradhaMatsumoto, IppeiBaskin, David S.Matsumoto, MisakoBasolo, FulvioMatsumoto, NaokiBasrai, MuniraMatsumura, NoriomiBassan, RenatoMatsusaki, TakashiBasu, AlakanandaMatsushita, KazuyukiBasu, ArnabMatsushita, MichikoBasu, DevrajMatsuzaki, ShinyaBasu, ReetobrataMattei, FabrizioBatchu, RameshMatter, Michelle L.Bateman, AndrewMatters, Gail L.Bathula, Chandra SekharMatthew, BeneschBatie, MichaelMatthiesen, RuneBatish, MonaMattingly, Raymond R.Batra, SachinMattiucci, Gian CarloBatrakova, Elena V.Mattogno, Pier PaoloBatsché, EricMattoscio, DomenicoBatsios, PetrosMatulić, MajaBattistelli, CeciliaMatuschek, ChristianeBattistelli, MichelaMatúšková, MiroslavaBauchet, LucMaugeri, AndreaBauckneht, MatteoMaugeri, Andrea GiuseppeBauer, GeorgMaur, MichelaBauer, JessicaMaura, CalvaniBauer, JohannMaurea, SimoneBauer, Ursula E.Maurel, JoanBauman, GlennMaurer, JochenBaumgarten, PeterMaurer, StefanieBaumgartner, MartinMauri, GianlucaBaumgartner, UlrichMauri, GiovanniBaxevanis, Constantin N.Mauri, MarioBaxter, EvaMaurizio, MartiniBay, Boon-HuatMauro, ClaudioBay, PeterMauro, Francesca R.Bayda, SamerMaurya, ShailendraBazan, VivianaMavila, NirmalaBazhin, Alexandr V.Mavrogonatou, EleniBazzichetto, ChiaraMavroudis, DimitriosBeal, Eliza W.Mawhinney, ThomasBeato, MiguelMawrin, ChristianBeatson, Richard E.Maxim, Peter GBeaulieu, Jean-FrancoisMaycotte, PaolaBeauregard, Jean-MathieuMayerhoefer, MariusBeaven, Anne W.Mazan-Mamczarz, KrystynaBeberok, ArturMazor, OhadBechmann, NicoleMazroui, RachidBechter, Oliver E.Mazur-Bialy, AgnieszkaBeck, BenjaminMazzetti, SimoneBecker, Hubert DominiqueMazzoccoli, GianluigiBecker, JürgenMazzolini, Guillermo D.Becker, ThereseMazzoni, MaraBeckermann, Kathryn EbyMazzoni, MariaBeckner, Marie EMbulaiteye, SamBedi, DeepaMcAllister, Sean D.Bednarsch, JanMcBride, WilliamBednarska-Szczepaniak, KatarzynaMcBryan, JeanBednarski, Patrick J.McCarthy, DennisBeebe, StephenMcCarthy, RonanBeer, Ambros J.McClurg, UrszulaBeharry, Kay D.McComb, ScottBehling, FelixMcCormack, AnnBehrens, BiancaMcCormack, EmmetBehrens, JürgenMcCoy, MelanieBeier, Justus PatrickMcCready, JessicaBeig, NihaMccullough, MichaelBekeschus, SanderMcDonald, PatrickBekiaris, VasileiosMcDonnell, AlisonBekkers, Ruud L. M.McEwan, IainBel’skaya, LyudmilaMcFadyen, JamesBelardinilli, FrancescaMcGovern, JacquiBelfiore, AntoninoMcGowan, EileenBelka, ClausMcIntosh, Stuart A.Bell, DianaMcKelvey, Kelly J.Bellelli, RobertoMcKenna, SharonBellevicine, ClaudioMcKinney, Matthew StuartBellini, Maria IreneMcLean, KarenBeltran, ChrisMcMahon, StephenBenada, JanMcManus, KirkBenavente, Claudia A.Mcmorrow, TaraBenbrook, Doris MangiaracinaMcMullin, Mary FrancesBender, DirkMcReynolds, Lisa J.Benesch, Matthew G. K.McSorley, Stephen T.Benevolenskaya, ElizavetaMd Kamal, HossainBenfeitas, RuiMeazza, RaffaellaBengtsson, EwertMedema, Jan PaulBenihoud, KarimMedina, PilarBenini, StefaniaMedina, Reinhold J.Benjamin, DonMedina-Ramírez, IlianaBenjamin, Robert S.Medinger, MichaelBenna, ClaraMedipally, DineshBennani-Baïti, BarbaraMedová, MichaelaBennewith, Kevin L.Meehan, KatieBennewitz, Margaret F.Meer, MargaritaBensussan, ArmandMego, MichalBentel, Jacqueline M.Mehedi, MasfiqueBenusiglio, PatrickMehra, NivenBenzaquen, JonathanMehta, AmitkumarBen-Ze’ev, AvriMehta, Gaurav A.Berardi, Anna ConcettaMehta, MeghnaBerasain, CarmenMehta, Nihar J.Berchner-Pfannschmidt, UttaMehta, SunaliBerdel, Wolfgang E.Mehta-Shah, NehaBerezowska, SabinaMei, MatthewBergamini, ChristianMei, YangBerger, Martin R.Meier, Kathryn E.Berger, MassimoMeierjohann, SvenjaBergers, GabrieleMeignan, MichelBergh, AndersMeijer, HannekeBerlien, H.-PeterMeijerink, Martijn R.Berlow, Noah E.Meinhardt, MatthiasBernardes De Jesus, BrunoMeiser, JohannesBernardes, NunoMejzlik, JanBernardi, SimonaMelcón, GallegoBernardini, GiuliaMeleady, PaulaBernardo, CarinaMelenhorst, Jan JosephBerndt, Michael C.Melin, Beatrice S.Bernemann, ChristofMellai, MartaBernocchi, GraziellaMelman, ArtemBernstein, Kara A.Melo, Sonia A.Berraondo, PedroMencke, RikBerrino, LiberatoMendelsohn, Robin B.Berry, JesseMendogni, PaoloBertacchini, JessikaMendonca-Torres, Maria CeciliaBertero, LucaMendoza, ManuelBerthelet, JeanMendoza-Nuñez, Victor ManuelBertolini, FrancescoMendyk, AleksanderBertoni, FrancescoMenè, PaoloBertozzi, SerenaMenendez, DanielBerwick, MarianneMenendez, Sofia T.Berzaczy, DominikMeneses, Patricio I.Besic, NikolaMénétrier-Caux, ChristineBesse, LenkaMeng, XiangbingBessette, BarbaraMennerich, DanielaBest, JanMenon, Manoj B.Best, Simon R.A.Meraviglia, SerenaBeswick, Ellen J.Mercadante, SebastianoBetés, Maite TeresaMercan, EzgiBettaieb, AliMercatali, LauraBettencourt-Silva, RitaMercedes, DuránBetts, Brian ChristopherMercury, Santo RaffaeleBetz-Stablein, BrigidMerino Peláez, GraciaBeverly, Levi J.Merino, Jaime M.Bewersdorf, Jan PhilippMerino, Jose JoaquinBezdetnaya-Bolotine, LinaMerino, RamónBezombes, ChristineMerola, ElettraBEZU, LucilliaMerolla, FrancescoBhaduri-McIntosh, SumitaMerollini, KatharinaBhagirath, DivyaMeroni, GermanaBhalla, NikhilMerx, MarcBharati, SanjayMeryet-Figuière, MatthieuBhardwaj, RajeshMerz, MaximilianBhardwaj, VikasMesguich, CharlesBhat, KamakotiMessaritakis, IppokratisBhat, SaleemMessiha, HadiBhattacharjee, SonaliMessina, CarloBhattacharya, RajatMessina, SamanthaBhattacharya, SantanuMetere, AlessioBhawal, Ujjal KumarMetrakos, PeterBhowmick, Neil A.Mettler, Lieselotte L.Bi, Andy ChengfengMetzger, Herr PhilippBi, MingjunMetzger, Monika L.Białek, AgnieszkaMetzler, MarkusBiałopiotrowicz, EmiliaMeyer, BernhardBianchi, FabrizioMeyer, Hans-JonasBianchi, FrancescaMeyer, Till JasperBianchi, MarziaMeyer, TimBianchini, FrancescaMeyers, CraigBianchini, LaurenceMezencev, RomanBianco, AndreaMian, HiraBianco, RobertoMiao, LinglingBibault, Jean-EmmanuelMiazek, ArkadiuszBibeau, FrédéricMicale, NicolaBidet, YannickMicalizzi, DouglasBidoli, EttoreMicha, DimitraBiegala, MichalMichaeu, OlivierBielak-Żmijewska, AnnaMichal, FiedorowiczBierhoff, HolgerMichalopoulos, IoannisBiersack, BernhardMichelhaugh, Sharon KayBiggar, KyleMichelle, PalmieriBiglia, NicolettaMichiels, CarineBijlsma, Maarten F.Michiko, IchiiBikas, AthanasiosMichl, JohannaBilancio, AntonioMichlewski, GracjanBilek, RadovanMidde, Krishna K.Bilger, AndreaMiddleton, Ryan J.Bill, MariusMielko, JerzyBille, AndreaMiettinen, MarkkuBillings, StevenMiglio, AriannaBinder, HansMihara, KeichiroBinotto, GianniMikala, GaborBinsthok, AlexanderMikami, MikioBiondi, AndreaMikheev, AndrejBiondi, AntonioMiki, YasuhiroBirch, DavidMikkilineni, LekhaBird, RanjanaMikropoulos, ChristosBirgin, EmrullahMilani, MarioBirkbak, NicolaiMilavetz, BarryBishayee, AnupamMilbourne, AndreaBishnupuri, Kumar S.Milcarek, ChristineBishop, Michael W.Milczarek, MagdalenaBismar, TarekMilczarek, MałgorzataBissonnette, MarcMild, Kjell HanssonBiswas-Fiss, Esther E.Milenkovic, Vladimir M.Bjørge, LineMiles, LindseyBjörkhem-Bergman, LindaMiletic, HrvojeBlache, PhilippeMilgrom, SarahBlack, Esther P.Milisav, IrinaBlack, JenniferMillar, EwanBlackburn, JessicaMiller, Anthony B.Blaheta, RomanMiller, JimBlair, Geoger EricMiller, John H.Blanco-Aparicio, CarmenMiller, PhilipBlanco-Fernandez, BarbaraMiller, VandanaBlandino, GiovanniMills, KenBlank, SusanneMills, KerryBlasiak, JanuszMills, Lauren J.Błaszczyk, JarosławMills, SamanthaBlattner, ChristineMillward, StevenBlay, Jaen-YvesMimche, Patrice NsangouBlenda, AnnaMimura, JunseiBloch, CharlesMina, RobertoBloise, EnrricoMinafra, LuigiBlondel, MarcMinaga, KosukeBlonska, MarzennaMinami, YasunoriBlyth, Benjamin J.Minamikawa, TakeoBoaz, KarenMinasian, Lori M.Bobe, RegisMinelli, RosalbaBobiński, MarcinMinervini, Crescenzio FrancescoBoccardo, FrancescoMinervini, GiovanniBock, NathalieMinguela, AlfredoBodily, JasonMinic, ZoranBødtkjer, EbbeMinichsdorfer, ChristophBoehm, Bernhard O.Miniero, Daniela ValeriaBoehm, DanielaMinna, EmanuelaBoeri, MattiaMino-Kenudson, MariBoers-Sonderen, MaryeMinoo, ParhamBoetto, JulienMinucci, SergioBoffano, PaoloMinutolo, AntonellaBogdanov, MikhailMinutolo, FilippoBoggio, ElenaMio, CatiaBoichuk, SergeiMiotla, PawelBoilève, AliceMiotto, BenoitBoj, Sylvia F.Miquel, AdroverBojarczuk, KamilMir, HinaBol, Kalijn FredrikeMirabelli, PeppinoBolam, Kate A.Mirabile, AuroraBoland, Mary ReginaMiranda, CristobalBoldorini, RenzoMiranda, EnriqueBoldrini, LucaMiranda-Gonçalves, VeraBölke, EdwinMirey, GladysBolle, StephanieMirjolet-Didelot, CélineBolm, LouisaMirshahi, MassoudBommareddy, Praveen K.Mirzaei, SiroosBommireddy, RamireddyMirzayans, RazmikBonanno, LauraMisaka, TomofumiBonavina, LuigiMiserocchi, GiacomoBonavita, EduardoMishra, AvanishBoncela, JoannaMishra, RosalinBond, JonathanMishra, Sandeep KumarBonecchi, RaffaellaMisso, GabriellaBonelli, Mara A.Mistretta, Francesco AlessandroBonenkamp, JohannesMistriotis, PanagiotisBonetti, AlessandroMita, KojiBonetti, MarcoMitamura, TakashiBonetto, AndreaMitani, SeiichiroBongiovanni, AlbertoMitchell, PaulBongiovanni, MassimoMitjans, FrancescBönig, HalvardMitra, RanjanaBONNEFOY, NathalieMitra, SumeghaBonnet, Pierre-AntoineMitsiadis, Thimios A.Bonnin, PhilippeMitsogiannis, IraklisBonomi, PhilipMitsuaki, MoriyamaBonomo, PierluigiMittal, KarunaBonora, ElenaMittal, SonamBonzheim, IrinaMittempergher, LorenzaBooken, NinaMiura, OsamuBoomer, Jonathan S.Miwa, ShinjiBoone, Brian A.Miyabe, KatsuyukiBoonen, KurtMiyake, MakitoBooth, BrianMiyamoto, HiroshiBoppana, Nithin B.Miyamoto, IkuyaBorcherding, NicholasMiyamura, TakakoBordonaro, MichaelMiyanaga, AkihikoBorger, JessicaMiyazaki, MasaruBorgia, Jeffrey A.Miyazawa, KeijiBorgmann, KerstinMiyazawa, MasaakiBorgo, ChristianMiyoshi, EijiBorkowska, Edyta MartaMiyoshi, HiroakiBornfeld, NorbertMiyoshi, HiroyukiBornhorst, MiriamMiziak, BarbaraBorovac, Josip A.Mizumoto, MasashiBorrebaeck, Carl A.K.Mizuno, ShugoBorrello, Maria GraziaMladenov, EmilBorsani, OscarMlynarczyk, DariuszBorse, VikrantMo, FeiBørset, MagneMoaven, OmeedBorstnar, SimonaMocellin, SimoneBos, GerardMoch, HolgerBose, TanimaMochizuki, SatoshiBosi, AlbertoMócikov́a, HeidiBosman, FredMöckelmann, NikolausBosnjak, MasaModak, ShakeelBosserhoff, Anja KatrinModerau, NinaBossi, PaoloModjtahedi, HelmoutBost, FrédéricMódos, DezsőBostjancic, EmanuelaModrowski, DominiqueBotella, Luisa MaríaMoen, ErikaBöttcher, ArneMoertel, ChristopherBotti, GerardoMohamed, BashirBottino, CristinaMohammed, AltafBotton, ThomasMohanty, Smruti R.Boublikova, LudmilaMohapatra, PurusottamBouchal, JanMohr, ThomasBouche, GauthierMoia, RiccardoBouchier-Hayes, LisaMoik, FlorianBouffet, EricMoineddin, RahimBoufraqech, MyriemMoiola, CristianBougen-Zhukov, NicolaMolenaar, Remco J.Bouhnik, Anne-DeborahMoletta, LuciaBoulaiz, HouriaMolfetta, RosaBoulter, LukeMolica, StefanoBoumber, YanisMolinari, ChiaraBourdeaut, FranckMolina-Vila, Miguel ÁngelBourdon, Jean-ChristopheMolinier-Frenkel, ValérieBousbaa, HassanMoll, HerwigBousquet, CorinneMøller, PålBoussios, StergiosMolloy, MarkBoutros, JacquesMolnar, Béla ÁkosBouvet, MichaelMolon, BarbaraBouvier, Anne-MarieMoloney, Gerard M.Bovée, Judith V.M.G.Momeny, MajidBowen, Thomas ScottMoncharmont, BrunoBowman, CaitlynMondal, GoutamBown, StephenMondal, SomritaBoyd, Kevin D.Mondal, Sujan KBoyle, Glen MMondal, Utpal KumarBozec, AlexandreMondelli, Mario U.Bożena, Leszczyńska-GorzelakMondello, ChiaraBozic, JoskoMondet, JulieBraat, AndriesMonfardini, LorenzoBrabek, JanMonga, VarunBracarda, SergioMongin, Alexander A.Bracci, MassimoMönig, StefanBracco, EnricoMonk, PeterBracken, Cameron P.Monni, OutiBradbury, AngelaMonreal, ManuelBradley, JulieMontalbano, MauroBrady-Kalnay, Susann M.Montanes, AntonioBragazzi, Nicola LuigiMontecucco, AlessandraBraicu, CorneliaMonteiro, CarlosBrainson, ChristineMonteleone, GiovanniBraithwaite, AntonyMontemagno, ChristopherBranca, Jacopo Junio ValerioMontemurro, NicolaBrancati, NadiaMontero, ÁngelBranco, CristinaMontesarchio, DanielaBrancolini, ClaudioMontfort, AnneBrandau, SvenMontgomery, McKaleBrandi, GiovanniMonti, SerenaBrandl, AndreasMontiel-Duarte, CristinaBrandsma, DietaMontironi, RodolfoBrandt, BurkhardMontone, Kathleen T.Branford, SusanMontopoli, MonicaBrantley, EileenMontrone, MicheleBrasso, KlausMoore-Carrasco, RodrigoBratt, OlaMora, JaumeBraud, Veronique M.Morabito, AlessandroBraun, RalfMorales, AlbertBraun, ThorstenMorales, Jorge A.Braunschweig, TillMorales, JulioBravatà, ValentinaMoramarco, AntoniettaBray, Laura JaneMorandi, AndreaBreccia, MassimoMorandi, LucaBregni, GiacomoMorbidelli, LuciaBreier, GeorgMoreaux, JeromeBreit, Samuel N.Moreira, AndreBrekken, Rolf A.Morelli, EugenioBrendel, JohannesMorelli, Maria BeatriceBrenet, FabienneMoreno, Carlos S.Brennan, DonalMoresco, RosaMariaBrennan, PaulMoret, FrancescaBrenner, Annette K.Moretti, FabiolaBrenner, J. ChadMorgagni, PaoloBresson-Bepoldin, LaurenceMorgan, AngelaBrewer, Alison C.Morgan, Ethan L.Brewer, Jerry D.Morgan, Joshua TBria, EmilioMorgan, MichaelBridle, KimMorgan, RichardBrieger, AngelaMorganti, Alessio G.Brigante, GiuliaMorganti, Alessio GiuseppeBrigelius-Flohe, ReginaMorgenstern, DanielBrindell, MałgorzataMori, MatteoBrindisi, MatteoMoriguchi, MichihisaBrindley, DavidMorikawa, TakamichiBrioli, AnnamariaMorini, MartinaBrix, KlaudiaMoris, Demetrios N.Briz, OscarMorita, YoshihiroBroekgaarden, MansMorizane, ChigusaBroering, RuthMorjani, HamidBroggini, MassimoMorland, BruceBronner, ChristianMoro, LoredanaBronte, EnricoMoro, MassimoBronte, FabrizioMoroni, DavideBrooke, GregMoros, EduardoBrooks, AllenMorotti, AlessandroBrooks, JenniferMorra, FrancescoBros, MatthiasMorreau, HansBrosens, ErwinMorrione, AndreaBroude, EugeniaMorris, AndrewBrounais-Le-Royer, BénédicteMorris, DavidBrousset, PierreMorris, DonBrouwer, Rutger W.W.Morris, Donald G.Brown, MichaelMorris, MelanieBrown, NelsonMorris, MhairiBrozovic, AnamariaMorrone, AldoBrozyna, AnnaMortara, LorenzoBruce, Jason I. E.Mortimer, PeterBrugarolas, PedroMoscetti, LucaBruggeman, Sophia W.M.Moschetta, MicheleBruix, JordiMoschini, MarcoBrunelli, MatteoMoschovi, MariaBrunet-Possenti, FlorenceMosgöller, WilhelmBrunetti, OronzoMosialos, GeorgeBrunetti-Pierri, NicolaMoss, Esther LouiseBruni, PaolaMoss, WalterBrunne, MaximilianMostofa, Agm G.M.Brunner, CorneliaMotamed, CyrusBrunner, MarkusMotegi, AtsushiBrünnert, DanielaMotiwala, TasneemBruno, AnnalisaMotohashi, HozumiBruno, AntoninoMotoi, FuyuhikoBruno, EleonoraMotterlini, RobertoBruno, RobertMou, YongchaoBruno, RossellaMouillet-Richard, SophieBruno, WilliamMoulière, FlorentBruns, ChristianeMoulin, AlexandreBruserud, ØysteinMountzios, GiannisBruzzese, FrancescaMourtada-Maarabouni, MirnaBrzóska, KamilMouw, Kent W.Bubendorf, LukasMouzaki, AthanasiaBuccisano, FrancescoMravec, BorisBuchan, RossMT, SkowronskiBuchbinder, ElizabethMu, PingBüchler, TomášMuallem, Mustafa ZelalBuckanovich, RonaldMucke, Hermann A.M.Buckingham, LelaMuders, MichaelBuckley, LizMudryj, MariaBuckley, Shannon M.Muehlebach, MichaelBuda, Valentina OanaMuether, MichaelBuderath, PaulMuhammad, NaoshadBudzevich, Mikalai M.Mühlebner, AngelikaBuechler, ChristaMuhlethaler-Mottet, AnnickBueno-Martínez, ElenaMu-Hsin, ChangBuffi, Nicolò MariaMuinelo-Romay, LauraBugajski, MarekMuir, AlexanderBui, Duc-CuongMukaida, NaofumiBui, Nam QuocMukherjee, AbhikBujanda, LuisMukherjee, AbirBujko, MateuszMukherjee, NabanitaBulatov, EmilMukherjee, PriyabrataBulboacă, Adriana ElenaMukherjee, SarbajitBulic-Jakus, FlorianaMukherjee, SudipBulin, Anne-LaureMukhopadhyay, SumanBulk, EtmarMulak, AgataBunce, Chris M.Mulder, Frits I.Bundschuh, Ralph A.Mulholland, DavidBunevičius, AdomasMüller, Gerd A.Bungau, SimonaMuller, Jean-MarcBunin, Greta R.Müller, Klaus GottlobBunse, LukasMüller, Kristian MarkBuonaguro, LuigiMuller, LaurentBurdette, Joanna E.Muller, Patricia A. J.Burek, MalgorzataMüller, VolkmarBurga, Laura N.Multhoff, GabrieleBurga, RachelMumoli, NicolaBurger, Michael C.Munger, KarlBurgess, AndrewMuniraj, NethajiBurman, PiaMuniyan, SakthivelBurnier, JuliaMunkarah, AdnanBurow, Matthew EMuñoz-Galván, SandraBurtea, CarmenMunshi, AnupamaBurtness, Barbara AnnMunshi, Hidayatullah G.Burton, Eric C.Münster, TinoBuscail, LouisMuntean, Andrew G.Bušek, PetrMunusamy, ElangoBuske, ChristianMupo, AnnalisaBusse, AntoniaMuraca, MaurizioBussmann, Rainer W.Murai, JunkoBussolati, OvidioMurakami, NaoyaButturini, ElenaMurakami, YoshikiBuzas, KrisztinaMurata, KazutoshiBužgová, RadkaMurata, MarikoBychkov, AndreyMurata, SoichiroByrd, Alicia K.Murata, TakayukiByron, SaraMurín, RadoslavByun, SanguineMuro, KeiCaba, OctavioMurovska, ModraCaballero, PabloMurovska, Modra F.Cabeza, LauraMurphy, AndrewCabibbo, GiuseppeMurphy, William J.Cacchione, StefanoMurray, KatieCaceres, SaraMurray, PaulCackowski, FrankMurtaza, MuhammedCadamuro, MassimilianoMusani, VesnaCaderni, GiovannaMuscarella, Lucia AnnaCaers, JoMuscari, FabriceCaffo, OrazioMuscolini, MichelaCagnin, StefanoMusi, GennaroCai, BishuangMusquera, MireiaCai, ChunyuMussolin, LaraCai, HoujianMustacchi, GiorgioCai, LishengMustachio, LisaCai, LuMusteanu, MonicaCai, QiuyinMustjoki, SatuCai, WenliMutti, LucianoCai, YingyunMuylle, KristoffCairo, StefanoMuz, BarbaraCaja, LaiaMuzii, Vitaliano FrancescoCalabrese, GianpieroMuzio, MartaCalabrese, GiovannaMuzza, MarinaCalabrese, VittorioMyers, DouglasCalabrò, ViolaMyers, Jamie S.Calderaro, AdrianaMymryk, JoeCalderon Larranaga, AmaiaMythreye, KarthikeyanCaliò, AnnaMytych, JenniferCalitz, CarlemiNabissi, MassimoCalò, Pietro GiorgioNaccarati, AlessioCalò, ValentinaNachmias, BoazCalogero, AntonellaNadal-Serrano, MercedesCalorini, LidoNadanaka, SatomiCaltabiano, RosarioNadiminty, NagalakshmiCalvaruso, MarcoNagano, HiroakiCalvez-Kelm, Florence LeNagarajah, JamesCalvi, BrianNagarajan, PrabakaranCalvisi, Diego F.Nagarajan, PriyadharsiniCalzado, Marco A.Nagasaka, KazunoriCama, AlessandroNagashima, HiroakiCamacho Páez, JoséNagel, StefanCamacho-Arroyo, IgnacioNagelkerke, AnikaCameron, DonaldNaghavi, Arash O.Cameron, DonnieNaghibalhossaini, FakhraddinCameselle-Teijeiro, Josè ManuelNagore, EduardoCamiolo, GiuseppinaNagulapalli Venkata, KalyanCamorani, SimonaNagy, Előd ErnőCampa, Carlo CosimoNagy, NoemiCampana, StefaniaNahi, HarethCampanella, AlessandroNahm, ChrisCampbell, BelindaNahmias, ClaraCampbell, KerryNaik, R. AkshataCamphausen, Kevin A.Naik, ShruthiCampione, ElenaNaillat, FlorenceCanale, MatteoNair, Asha A.Cananzi, Ferdinando Carlo MariaNair, GopikaCANBERK, SuleNair, Praful R.Cancemi, PatriziaNair, SreenathCandeias, MarcoNaito, YutakaCândido Carvalho, KátiaNajimi, MustaphaCandiota, AnaNajjar, Yana G.Canela, Enric I.Nakagawa, MasatoshiCanesin, GiacomoNakahara, TomomiCanevari, SilvanaNakai, YousukeCani, AndiNakajima, AtsushiCannon, MartinNakajima, TakahitoCanter, Robert J.Nakajima, YasuakiCantile, MonicaNakamaki, TsuyoshiCao, BoNakamoto, AtsushiCao, DeliangNakamura, AsakoCao, YihaiNakamura, KoheiCaparros-Martin, JoséNakamura, KyoheiCapasso, RaffaeleNakamura, MasatoCapella, CarloNakamura, TakuroCapelli, LauraNakamura, TomokiCapillo, GioeleNakamura, YasuhiroCapitini, Christian M.Nakamura, YoshiyukiCaplen, Natasha J.Nakamura, YusukeCapobianco, EnricoNakanishi, TakeoCapodiferro, SaverioNakano, KenjiCapoluongo, EttoreNakano, MasahitoCappabianca, SalvatoreNakarmi, UkashCappariello, AlfredoNakashima, YoshikiCappellesso, RoccoNakata, WataruCappuzzo, FedericoNakato, GakuCapuano, AlessandraNakatomi, HirofumiCapuano, CristinaNakayama, JunCapurso, GabrieleNakayama, KohCaramalho, IrisNakayama, MizuhoCarante, Mario PietroNakayama, NaomiCarbognin, LuisaNakazawa, TsutomuCarbone, AntoninoNalewajko, Joanna SekulskaCarbone, CarmineNalvarte, IvanCardillo, GiuseppeNam, Eun JiCardobi, NicolòNam, SeungyoonCardonick, Elyce HNambiar, Dhanya K.Cardoso, Ana Maria S.Namdar, AfshinCardoso, Bruno AntónioNami, BabakCare, AndrewNanba, KazutakaCaretti, GiuseppinaNandana, SrinivasCarew, Jennifer S.Nandy, Sushmita BCaria, PaolaNannini, MargheritaCarina, ValeriaNanoff, ChristianCariou, KevinNanou, AfroditiCarlino, MatteoNapoletano, ChiaraCarlomagno, ChiaraNapoli, SaraCarmichael, Gordon G.Napolitano, MariasantaCarneiro, PatríciaNapolitano, RobertaCarnero, AmancioNappi, LuciaCarnesecchi, JulieNarayan, PrakashCaron, Joan McIntyreNarayanan, BarathCarpenter, RichardNarayanan, RameshCarpentier, DavidNardi, ValentinaCarpi, SaraNardone, ValerioCarrà, GiovannaNarni-manchinelli, EmilieCarrer, AlessandroNarod, Steven A.Carreras, JoaquimNasarre, PatrickCarrier, FranceNash, SarahCarrier, PaulNass, NorbertCarriero, Maria VincenzaNassa, GiovanniCarter, Carol A.Nassar, NicolasCarter, WayneNasser, Nicola J.Carullo, GabrieleNassiri, MehdiCaruntu, ConstantinNassour, IbrahimCarvalho, Ana Sofia LeitãoNastuta, Andrei VasileCarvalho, EdmundNatarajan, Sathish KumarCarvalho, JoanaNatarajan, SivaramanCarvalho, LinaNateri, Abdolrahman ShamsCasaburi, IvanNath, NiharikaCasalou, CristinaNath, ShubhankarCasanova, EmilioNatkunam, YasodhaCasanova, LlanosNatsumeda, ManabuCasares, NoeliaNaumann, PatrickCasari, AliceNaumann, UlrikeCasciati, AriannaNavarra, MicheleCascini, Giuseppe LucioNavarria, PierinaCascione, LucianoNaves, ThomasCaserta, DonatellaNavran, ArashCasey, JenniferNawaz, MuhammadCasimiro, SandraNawrocki, Steffan T.Caspari, ThomasNawroth, RomanCassano, EnricoNayak, TapanCassidy, Pamela BondNazaruk, EwaCassim, ShamirNazzaro, GianlucaCassinat, BrunoNdieyira, JosephCassinelli, GiulianaNdika, JosephCassoni, PaolaNeagoe, OctavianCassoux, NathalieNebreda, Angel R.Castanheira, Elisabete M.S.Nechaev, SergeiCastañón, EduardoNedeljković, MilicaCastelli, VanessaNeeb, AntjeCastiglia, MartaNeergheen-Bhujun, Vidushi S.Castillo-Tong, Dan CacsireNegroiu, GabrielaCastoria, GabriellaNelson, GreggCastresana, Javier S.Neophytou, Christiana M.Castriconi, RobertaNephew, Kenneth P.Catalan, MarceloNeri, AntoninoCatalani, ElisabettaNess, ScottCatani, LuciaNettelbeck, DirkCatanzaro, GiuseppinaNeubauer, HansCaterino, MariannaNeubauer, HeidiCato, Andrew C. B.Neubauer, KatarzynaCaudrelier, Jean-MichelNeufert, ClemensCauli, OmarNeuhaus, KlausCaux, ChristopheNeuhöfer, PatrickCava, ClaudiaNeuman, ManuelaCavaliere, GinaNeureiter, DanielCavalieri, StefanoNeven, PatrickCavalletto, LuisaNeves, Rui Pedro LousaCavallin, FrancescoNevi, LorenzoCavalloni, GiulianaNevler, AvinoamCavazzoni, AndreaNeychev, VladimirCaviglia, Gian PaoloNeyns, BartCaziuc, AlexandraNeyroud, DariaCecchetti, SerenaNG, Kevin Tak-PanCecinati, ValerioNg, LuiCeckova, MartinaNg, MatthewCelestino, RicardoNg, Ray KitCeletti, AngelaNgen, Ethel J.Cemazar, MajaNgô, CharlotteCenni, VittoriaNgoi, NatalieCeppi, LorenzoNguyen, Andrew V.Ceraline, JocelynNguyen, FrédériqueCerdán, María-EsperanzaNguyen, Joe TruongCeresa, BrianNguyen-Khac, EricCerretti, RaffaellaNi, XiaoCerrito, Maria GraziaNiarakis, AnnaCerrone, MargheritaNiazi, KhalidCeruti, StefaniaNice, EdouardCervello, MelchiorreNichetti, FedericoCerveny, LukasNickel, BrookeCervera, JoséNickel, FelixCerwenka, HerwigNickerson, Jeffrey A.Cesselli, DanielaNicod-Lalonde, MarieCestmir, AltanerNicolato, AntonioCha, Hyuk-JinNicolay, NilsChaber, RadosławNicolini, AndreaChabot, BenoitNicoll, Amanda J.Chachami, GeorgiaNicolle, RemyChacko, Jenu VargheseNicoloso, MilenaChaddad, AhmadNicosia, AldoChaganty, BharatNiederwieser, DietgerChaix, Carole Nieland, John Dirk VestergaardChakrabarti, JayatiNielsen, Finn CiliusChakrabarti, KausikNielsen, MaartjeChakrabarti, RajarshiNielsen, Patricia S.Chakrabarti, RumelaNieminen, Taina T.Chakraborty, Abhishek A.Niemira, MagdalenaChakraborty, SanjuktaNiessner, HeikeChakraborty, SayanNieveen Van Dijkum, ElisabethChalamalasetty, Ravi B.Nighot, PrashantChalayer, EmilieNigris, Filomena DeChalla, DilipNiikura, MasahiroChallagundla, KishoreNijhof, IngerChaluvally-Raghavan, PradeepNijland, MarcelChamaraux-Tran, Thiên-NgaNijman, H.W. (Hans)Chamberlain, Casey ANikander, Riku W.Chan, AdenNikfarjam, MehrdadChan, AlexanderNikitovic, DraganaChan, Anthony W.H.Nikolaev, ViacheslavChan, GordonNikolaos, MachairasChan, Shih-HsuanNiland, StephanChan, WanNilsson, Inga-LenaChandhok, NamrataNilsson, JonasChandler, RonaldNilubol, NarisChandra, Partha KNirala, Niraj K.Chandra, VishalNishida, HarutoChandrakesan, ParthasarathyNishida, HirokoChandran, UmaNishida, ShozoChandrasekaran, AkshayaNishida, ToshiroChang, Audrey N.Nishikawa, HirokiChang, Cheng-ChangNishikawa, JunChang, Chien-ChungNishimura, NoriyukiChang, Chih LongNishio, MizuhoChang, HongNishio, ShinChang, Hsueh-WeiNishioka, HiroshiChang, HyunNishizawa, ToshihiroChang, Jer-HaoNitta, TakayukiChang, Jia-FengNitulescu, George MihaiChang, Jinghua TsaiNitzsche, BiancaChang, Kai-PingNixon, AndrewChang, Kee-LungNiyongere, SandrineChang, Kung-ChaoNjar, Vincent C.O.Chang, Kuo-WeiNobis, MaxChang, Li-ChingNoble, SimonChang, Ling-ChuNoda, MasaruChang, Long-SenNoël, GeorgesChang, Seon HeeNogova, LuciaChang, Shu-ChunNoguchi, ShunsukeChang, Wei-MinNogueira, PauloChang, Wen-WeiNoguera, Nélida InésChang, XiaoNoguera, RosaChang, Ying-ChihNoguera-Julian, MarcChao, K.S. CliffordNoguera-Ortega, EstelaChao, Wei-TingNoh, Jae MyoungChapple, SarahNohata, NijiroChaput, NathalieNojima, HiroyukiCharles, KellieNolan, EmmaChastre, ÉricNollmann, FriederikeChatain, NicolasNonami, AtsushiChatelut, ÉtienneNones, KatiaChatterjee, IshitaNonnekens, JulieChatterjee, NimratNoonan, DouglasChatterjee, SampurnaNoonepalle, SatishChauchereau, AnneNoonepalle, Satish KumarChaudhari, KiranNoorani, ImranChaudhary, PankajNoordermeer, SylvieChauhan, MunishNorcic, GregorChauhan, NeerajNordström, AndersChautard, EmmanuelNorian, LyseChauvie, StephaneNorihiko, NaritaChavan, HemantkumarNoritaka, YamaguchiChaveroux, CedricNormanno, NicolaChávez, SebastiánNorton, JeffreyChavez-Calvillo, GabrielaNotni, JohannesChayama, KazuakiNottegar, AlessiaCheedipudi, SirishaNotter, MarkusChekenya, MarthaNoubissi, Felicite KamdemChemello, LilianaNovakova, ZoraChen, Bang-BinNovakovic, DanielChen, Chang-HanNovelli, FrancoChen, Chao-JungNovick, DanielaChen, Cheng YiNovickij, VitalijChen, Chien-ChinNovío, SilviaChen, Chi-LongNowak, MarekChen, Ching-ChowNowakowska-Zajdel, EwaChen, Chun-HanNowak-Sliwinska, PatrycjaChen, Chun-JungNowotarski, Shannon L.Chen, Chun-LiangNozaki, MasamiChen, DechanNtafou, DimitraChen, FeiNtanasis-Stathopoulos, IoannisChen, GuanNucci, DanieleChen, GuangpingNuciforo, PaoloChen, HailanNüesch, JürgChen, HaoNunes, AlexandraChen, HongwuNúñez-Iglesias, María JesúsChen, HuNurwidya, FarizChen, James L.Nuyts, SandraChen, JinbiaoNwosu, Zeribe ChikeChen, Jung-ChihNy, LarsChen, Ke-ChengNyayapathi, NikhilaChen, Lih-ChyangNylander, KarinChen, LongwenO. Othman, MohamedChen, MaoshanO’Byrne, KenChen, Mei-RuO’Cearbhaill, Roisin E.Chen, MingshengO’Connell, CatherineChen, Mu-KuanO’Connor, JamesChen, Pai-ShengO’Hagan, HeatherChen, Pei-NiO’Kane, Grainne M.Chen, Pin-WenO’Loughlin, DeclanChen, QiO’Neill, EricChen, Rong-JaneO’Rourke, ColmChen, Sean Chun ChangO’Shaughnessy, RyanChen, Sheng-HungO’Sullivan, MaureenChen, ShukunO’Toole, Martin G.Chen, ShuyangO’Toole, Sharon A.Chen, SuzieOancea, S. CristinaChen, TianjiObacz, JoannaChen, Ting-WenObara-Michlewska, MartaChen, Wei-ShengObermayr, EvaChen, Wei-YuObermeier, BirgitChen, WenchunObinata, DaisukeChen, XinOboki, KeisukeChen, XingchengObrador-Hevia, AntoniaChen, XingjuanOcaña, AlbertoChen, XinjianOchieng, JosiahChen, XuOddone, EnricoChen, Yann-JangOdenthal, MargareteChen, Yi CharlieOdland, JonChen, YingOehler, RudolfChen, YiyiOei, Arlene L.Chen, YuanOestergaard, Vibe HallundbækChen, Yu-ChihOestrup, OlgaChen, Yu-JenOffermann, AnneChen, Yung-ChihOfir, RivkaChen, Zhe-shengOgawa, TetsuyaChen, ZhitongOgino, TakashiChen, ZhongOh, Eok-SooChen, ZiqingOh, Eun-TaexCheng, DeOh, In-JaeCheng, HailiOh, SangphilCheng, HaiziOh, Seung HyunCheng, Hong ShengOh, YohanCheng, Jason Chia HsienOhama, TakashiCheng, JiongjiaOhashi, ManabuCheng, Kuang-HungOhashi, RiukoCheng, KunOhba, KojiroCheng, Shih-PingOhba, ShigeoCheng, Tsu-YaoOhga, NoritakaCheng, XiOhgami, Robert S.Cheng, Ya-YunOh-Hohenhorst, Su JungCheng, Yuen YeeOhkawa, KazuyoshiCheok, MeylingOhkuri, TakayukiCheon, Dong-JooOhlund, DanielCheon, Yong-PilOhno, HitoshiChern, EdwardOhnuki, HidetakaChernoff, JonathanOhtani, KiyoshiChernyshev, VasiliyOhtsuka, MasayukiChervoneva, InnaOhya, SusumuCheung, Annie Nga-YinOikawa, KosukeCheung, Kwok-LeungOing, ChristophChevalier, FrançoisOkada, TomoyoChevret, EdithOkafor, FlorenceChew, Valerie Suk PengOkamoto, KoichiChhabra, GaganOkamura, RyosukeChi, Kwan HwaOkegawa, TakatsuguChia, GlorynOklu, RahmiChiam, KarenOkosun, JessicaChiang, Chih-ShengOksenych, ValentynChiang, Chi-ShiunOktay, Maja H.Chiang, I-TsangOkubo, KazukiChiang, J.M.Okuda-Ashitaka, EmikoChiang, Ming-KoOkuma, YusukeChiang, Po-ChangOkumura, ToshiyukiChiang, Tung-chinOlbryt, MagdalenaChiang, WeichungOldenborg, SabineChiang, Ying-ChengOle Schmidt, NilsChiara, María-DoloresOliva, StefaniaChiaradonna, FerdinandoOliveira, PatríciaChiarion-Sileni, VannaOliveira, Paula A.Chiba, HideyukiOliveira-Ferrer, LeticiaChibon, FredericOliver, LisaChicas-Sett, RodolfoOliviero, BarbaraChieffi, PaoloOlnes, Matthew J.Chieh, Jen-JieOlofsson Bagge, RogerChien, Chih-YenOlsen, BirgitteChien, Chun-RuOlsen, MajaChien, JeremyOlsen, MarkChihara, DaiOlsson, Anna-KarinChina, ArnabOltolina, FrancescaChinegwundoh, FrancisOmar, Syed HarisChinello, CliziaOmene, Coral O.Chinen, Ludmilla Thomé DomingosOmodei, DanielaChinnappan, MahendranOng, QunxiangChiou, Tzeon-JyeOnken, MichaelChirgwin, John M.Ono, TakashiChistolini, AntonioOnoda, NaoyoshiChitale, ShalakaOo, Thein HlaingChitcholtan, KennyOosten, Astrid W.Chittori, SagarOosterwijk, EgbertChiu, Chien-ChihOosting, JanChiu, Ching-FengOpferman, JosephChiu, Hua-ShengOratz, RuthChiu, Ing-MingOrecchia, PaolaChiu, Ka Fung PeterOrekhov, Alexander N.Chiu, Yi-HanOrenes Piñero, EstebanChiuri, Vincenzo EmanueleOrgel, JosephChivero, ErnestOrimo, AkiraChmielowski, BartoszOriola, JosepCho, JeongheeOrlacchio, ArturoCho, Je-YoelOrlandi, RosariaCho, Joo-YounOrlov, Yuriy L.Cho, Jung SookOrlova, AnnaCho, Kang SuOrozco, JahirCho, Kwang-JinOrr, BernardoCho, NamhoonOrriss, IsabelCho, SungyupOrsburn, BenjaminCho, Sun-wookOrsi, NicCho, WilliamOrtega Sarmiento, SamuelCho, YongyeonOrth, James D.Chockley, PeterOrtiz, CarmenChocry, MathieuOrtiz, Michael V.Chodick, GabrielOry, StéphaneChoi, Chang-minOsaki, MitsuhikoChoi, HaesunOsaki, TomohiroChoi, Hyun-KyungOsawa, TakahiroChoi, June YoungOsborne, NickChoi, Jung-HyeOshi, MasanoriChoi, Kyeong SookOskeritzian, Carole A.Choi, Tae GyuOsterlind, KellChong, Charing Ching-NingOsti, Mattia FalchettoChong, GeoffOstrin, Edwin J.Chong, Gun OhOteri, GiacomoChong, Irene Yu-ShingOtero, CarolinaChong, Parskson Lee GauOthus, MeganChong, YosepOtoshi, TakehiroChorostowska-Wynimko, JoannaOtowa, YasunoriChottanapund, SuthatOtręba, MichałChou, C. JamesOtsuka, IsaoChou, Cheng-YangOtsuki, TakemiChou, Chih-HungOttaiano, AlessandroChou, Feng-ChengOttenhausen, MalteChou, Shih-FengOttewell, Penelope D.Chou, Wen-ChienOtto, TobiasChou, Yu-TingOu, Da-LiangChowdhury, Ezharul HoqueOuaissi, MehdiChowdhury, Wasim H.Ouro, AlbertoChrétien, Anne-SophieOvchinnikov, Lev P.Christensen, Jørn B.Ove, RogerChristian, Sherri L.Ovejero-Benito, María CarmenChristie, ElizabethOw, ThomasChristopher, LaRoccaOwczarczyk, KatarzynaChristopoulos, PetrosOwen, CarolynChu, Ching-LiangOwen, DwightChu, SimonOwen, KatieChu, Yueng-HsiangOwen, RichardChuang, Show-MeiOwens, PhilipChuang, Shuang-EnOyoshi, TakanoriChuang, Shu-ChunOzaki, IwataChuang, Wan-LongOzaki, ShujiChuang, Wen-YuOzbun, MichelleChueh, Anderly C.Özcelik, DennisChulanov, VladmirOzeki, MichioChuma, MakotoOzeki, YasuhiroChung, Byung MinOzer, Hatice GulcinChung, ChaeukOzsvari, BelaChung, Chi-LiÖzvegy-Laczka, CsillaChung, Ching-HuP. Williams, JacquelineChung, Jae HoonPaavonen, Jorma A.Chung, JinsooPabla, NavjotChung, Joon-yongPabst, ThomasChung, JunPace, AndreaChung, SanghyukPacheco, Maria PiresChung, Tae HunPackiriswamy, NandakumarChung, Yeun-JunPadera, TimothyChuntao, ZhaoPadler-Karavani, VeredChurg, AndrewPadmanabhan, JayaChwialkowska, KarolinaPaeschke, KatrinChwistek, MarcinPagano, AlessandraCi, XinpeiPagano, DuilioCiabrelli, FilippoPagano, MicheleCiaglia, ElenaPage, AndrewCianciosi, DanilaPagenstecher, AxelCiasca, GabrielePaggetti, JeromeCiavattini, AndreaPaggi, Marco G.Cicènas, SauliusPagni, FabioCicero, ArrigoPagotto, SaraCicero, GiuseppePaholcsek, MelindaCichorek, MiroslawaPahwa, RomaCidre-Aranaz, FlorenciaPaidi, Santosh KumarCifone, Maria GraziaPaiella, SalvatoreCifra, MichalPaíno, TeresaCifrián, José ManuelPainschab, MatthewÇiftçi, Halil İbrahimPaireder, MatthiasCifuentes-Rius, AnnaPaiva, ArturCildir, GokhanPal Choudhuri, ShreoshiCilloni, DanielaPal, DebjaniCimadamore, AlessiaPal, DhananjayCiminale, VincenzoPal, KrishnenduCimini, DanielePal, SoumitroCimmino, FloraPalacios Calvo, JoseCinar, BekirPalacios, FlorenciaCiorba, Matthew A.Paladino, Nunzia CinziaČipak Gašparović, AnaPalamà, IlariaCipak, LubosPalanisamy, NallasivamCipolloni, LuigiPalazzo, LucaCippitelli, MarcoPaleari, LauraCiribilli, YariPalladini, AriannaCisowsky, JaroslawPallasch, Christian P.Ciszewski, WojciechPallavicini, GianmarcoCiszewski, Wojciech MichałPalle, KomaraiahCitro, SimonaPallocca, MatteoCitterio, DavidePalma, MarziaCives, MauroPalmer, ChristopherCivita, ProsperoPalmer, StephenClaassen, EricPalmieri, DarioClamon, GeraldPalmieri, GiuseppeClanchy, FelixPalmqvist, RichardClaps, MelaniePalmucci, StefanoClark, AlisonPalomba, RobertoClark, Geoffrey J.Palumbo, GiuseppeClark, SusanPalumbo, Giuseppe AlbertoClassen, Carl FriedrichPaluri, RaviClément, BrunoPaluszczak, JarosławCloos, JacquelinePalviainen, MariCloos, PaulPan, Chin-chenCluzeau, ThomasPan, Chun-HsuCobbs, CharlesPan, EdwardCobellis, GildaPan, JiayiCoccaro, NicolettaPan, JingCocco, StefaniaPan, MiaoCochran, SandyPan, Tai-longCochrane, Dawn R.Pan, ZuiCoderch, ClairePancaldi, VeraCodony-Servat, JordiPandey, Dhananjay K.Coelho-Silva, JuanPandey, RituCoffey, David G.Pandya, Pankita H.Coffey, RobertPane, KatiaCoffman, Lan G.Panfil, AmandaCohen, GadiPang, LisaCohen, RomainPanjarian, ShoghagCohn, MartinPankratova, StanislavaCoker, Olabisi OluwabukolaPanneerdoss, SubbarayaluColaço, Bruno JorgePannico, MariannaColangelo, DonatoPantel, KlausCole, David K.Panthu, BaptisteCole, PeterPanuzzo, CristinaColeman, RobertPanza, SalvatoreColetti, DarioPanzuto, FrancescoColgan, NiallPaoli, DonatellaColin, DidierPaolillo, CarmelaCollart, Martine A.Paolillo, MayraCollavin, LicioPaolini, FrancescaColletti, MartaPaolino, GiovanniCollins, Christopher M.Paone, AlessioCollura, AdaPaone, G.Colombo, CarlaPapaccio, FedericaColombo, MichelaPapaccio, GianpaoloColomo, LluísPapachristodoulou, AlexandrosColonna, GiovanniPapadaki, CharaColuccia, MauroPapadaki, MariaColucci-D’Amato, LucaPapadakos, KonstantinosColumbano, AmedeoPapadopoulos, PetrosComan, DanielPapadopoulou, LefkotheaCombet, ChristophePapagiannopoulou, DionysiaCommane, DanielPapamichael, DemetrisConceição, RaquelPapamichail, DimitrisConde, CarlosPapanikolaou, NickolasConde-Estévez, DavidPapatheodoridis, George V.Condelli, ValentinaPapoutsoglou, PanagiotisCondello, MariaPapp, BélaConesa-Zamora, PabloParadiso, AngeloConese, MassimoParagliola, Rosa MariaConfavreux, CyrilleParanjape, AnuragConigliaro, AliceParashar, DeepakConnor, MarkParasido, ErikaConrad, David M.Paraskeva, EfrosyniConradi, LenaParayath, NehaConsalvi, SaraParayath, Neha N.Console, LaraParazzini, FabioConstantinescu, StefanParchem, Ronald J.Contaldo, MariaPardini, BarbaraConte, MariarosariaPardo, Olivier E.Conte, MassimoParent, CaroleConte, NathalieParente, PaolaConti, AlfredoParenti, RosalbaConti, LuciaParet, ClaudiaConvertini, PaoloParida, SheetalCook, GrahamParis, FrancoisCoombs, MelanieParis, JuanCooper, Sara J.Park, Chan YoungCopeland, NikkiPARK, Eun JeongCopik, AlicjaPark, Eun-JooCoppedè, FabioPark, Henry S.Cora’, DavidePark, Hyun WooCorazzi, LanfrancoPark, In JaCorbet, CyrilPark, Jae-BongCorbí, Angel L.Park, Jee SooCorcoran, Niall M.Park, Ji WonCorcos, LaurentPark, JongCordani, MarcoPark, Jong KookCordelier, PierrePark, Jong-InCordenonsi, MichelangeloPark, Jung-HoonCordes, NilsPark, Ki-cheongCorey, SethPark, Kyung-soonCornelis, FrancoisPark, MargaretCornelius, DenisePark, MyungjinCorominas, MontserratPark, Sue K.Corona, GiuseppePark, Tae-JunCorre, JillPark, Thomas In-HyeupCorrenti, MargheritaParker, AmeliaCorsello, Salvatore MariaParks, RuthCorsi, AlessandroParmentier, MarcCorsi, FabioParonetto, Maria PaolaCorsi, LorenzoParra-Herran, CarlosCorsini, MichelaParrington, JohnCorso, GiovanniParrozzani, RaffaeleCortes-Dericks, LourdesParsons, JasonCorvigno, SaraPartanen, MaritaCos, SamuelParvatiyar, MichelleCoscia, MartaPasanisi, PatriziaCosconati, SandroPasca, SergiuCossarizza, AndreaPascale, RosaCosset, ErikaPasche, BorisCosta, ClotildePascut, DevisCosta, João GuilhermePasini, LuigiCosta, José LuisPasmant, EricCosta, Marina C.Pasquali, ClaudioCosta, VivianaPasquali, SandroCostantini, ClaudioPasquet, Jean-MaxCostantini, LaraPasquet, MarleneCostantini, SusanPassera, RobertoCostelli, PaolaPasto, AnnaCostoya, Jose A.Pastushenko, IevgeniiaCote, Gregory M.Patankar, ManishCotella, DiegoPatel, Akash J.Cotelle, PhilippePatel, DevenCotignola, JavierPatel, Dhaval T.Cotsoglou, ChristianPatel, Girijesh KumarCotta, ClaudiuPatel, Jai N.Cottone, FrancescoPatel, NibeditaCougnoux, AntonyPatel, NiketaCoulouarn, CédricPatel, Sagar A.Coupland, SarahPatel, SanjayCourand, Pierre-Yves CourandPatel, Sapna P.Courbon, FredericPater, LukeCourt, Karem A.Pateras, Ioannis S.Coussons, PeterPaterson, AndrewCouttet, PhilippePaterson, Ian CharlesCovacu, RuxandraPaterson, YvonneCoventry, BrendonPatil, AnujaCoward, JermainePatnaik, SanthoshCowden Dahl, KarenPatocs, AttilaCox, AngelaPatra, Hirak K.Cox, DiannePatrícia, BarrosCoyle, BethPatricia, JohanssonCoyle, Krysta MilaPatrussi, LauraCrabb, JohnPatten, DavidCrabtree, JudyPattnaik, BikashCrawford, Bryan D.Paudel, Keshav RajCrawford, HowardPaul, CatherineCrawford, LisaPaulo, SiriCremolini, ChiaraPaulsson, Johan O.Crespo, PieroPausch, Thomas.Crezee, HansPauwels, PatrickCrezee, JohannesPavlidis, EfstathiosCrippa, StefanoPavlik, EdwardCrisafulli, GiovanniPavlik, Edward J.Cristiani, Costanza MariaPavlin, MaticCristina, CampiPavlov, Youri I.Cristofanilli, MassimoPavlova, ElenaCrivii, CarmenPavlova, GalinaCrompton, Joseph G.Pavlovic, NatasaCrona, DanielPawar, Shrikant D.Croset, MartinePaweł, KalińskiCross, NeilPawelek, JohnCsordas, GyorgyPayne, AnnetteCubiella, JoaquinPayne, Karl Frederick BraekkanÇubuk, CankutPayne, KimberlyCuburu, NicolasPazzaglia, LauraCuccarini, ValeriaPearse, Roger N.Cucchetti, AlessandroPearson, Dustin D.Cui, QingbinPeccatori, FedroCui, TiantianPecce, ValeriaCulig, Zoran CuligPecchi, AnnaritaCumbo, CosimoPECHEUR, EveCunningham, RuthPeck, BarrieCuocolo, RenatoPecorari, GianCarloCuppoletti, JohnPecoraro, AngelaCurado, Maria PaulaPecqueur, ClaireCurrie, MargaretPedersen, Inge SøkildeCurtale, GraziellaPeethambaran, BelaCurti, AntonioPegoraro, GianlucaCurtin, NicolaPei, YonggangCuschieri, KatePeinemann, FrankCushen, Samantha J.Peiris, CaseyCutone, AntimoPeitzsch, ClaudiaCybulski, CezaryPeixoto, PaulCyril, SobolewskiPejchal, JaroslavCzapiewski, PiotrPejin, BorisCzarniecka, AgnieszkaPellecchia, SimonaCzubryt, MichaelPellegatta, SerenaCzyz, AnnaPellerino, AlessiaCzyż, JarosławPellicano, RinaldoCzyżewski, KrzysztofPemov, AlexanderD Prestwich, Robin J.Peña, CristinaD’Agnano, IgeaPena, Maria MarjoretteD’Agostino, Vito GiuseppePeña-Llopis, SamuelD’Aguanno, SimonaPenalva, LuizD’Alessandra, YuriPencreach, ErwanD’Alessandria, CalogeroPende, DanielaD’Alò, FrancescoPendino, FredericD’Amico, Agata GraziaPenela, PetronilaD’Andrea, DavidPeng, AiminD’Andrea, VitoPeng, DunfaD’Angelo, EdoardoPeng, GuangyongD’Angelo, MichelePeng, HanD’Anneo, AntonellaPeng, I-ChenD’Arcy, PadraigPeng, QianD’Argenio, ValeriaPennock, NathanD’auria, ValeriaPentimalli, FrancescaD’Avella, DomenicoPepe, StefanoD’Azzo, AlessandraPeperzak, VictorD’Elios, Mario MilcoPeppelenbosch, M. P.D’Incalci, MaurizioPeppelenbosch-Kodach, LiudmilaD’Orazi, GabriellaPeppicelli, SilviaD’Urso, AlessandroPeraldo-Neia, CaterinaDa Pieve, ChiaraPeran, IvanaDa Rocha, Simao TeixeiraPercesepe, AntonioDa Silva, Sabrina DanielaPerdas, EwelinaDabernat, SandrinePerego, MichelaDabo-Niang, SophiePereira, Allan A.Dachineni, RakeshPereira, BrunoDaftarian, PirouzPereira, DavidDahlmann, MathiasPereira, StephenDai, HaimingPerentes, Jean YannisDai, WeiPérès, Elodie A.Daigeler, AdrienPeretti, GiorgioDal Col, JessicaPérez-Campo, Flor MaríaDalamaga, MariaPerez-Hernandez, ConcepciónDalby, Kevin N.Pérez-Lluch, SílviaDalekos, George N.Pérez-Plasencia, CarlosDalla Pozza, ElisaPerez-Ruiz, ElizabethDalla, EmilianoPerez-Stable, CarlosDamato, BertilPérez-Torras, SandraDambrauskas, ZilvinasPergolini, IlariaDamia, GiovannaPerini, GiordanoDamiani, DanielaPeris, KettyDammann, ReinhardPerisetti, AbhilashDamotte, DianePerkins, NeilDandawate, PrasadPerl, AndrasDandekar, AdityaPerła-Kaján, JoannaDandekar, GudrunPero, RaffaelaDangle, PankajPérol, DavidDaniele, AuroraPeron, JulienDaniele, GennaroPerri, FrancescoDanielson, KirstyPerris, RobertoDanila, Daniel C.Perrone, Anna MyriamDanila, EdvardasPersano, StefanoDanilenko, MichaelPersichetti, PaoloDann, EldadPersico, MarcelloDansette, Patrick M.Persson, EmmaDantzer, FrançoisePersson, JennyDar, Mohd SaleemPerucho, ManuelDario, Mandato VincenzoPervaiz, ShazibDarrasse-Jeze, GuillaumePesce, SilviaDas, GokulPessina, FedericoDas, RajdeepPessoa, RodrigoDas, RupaliPeták, IstvánDas, SudipPeters, Godefridus J.Das, SunitPetersen, Ole WilliamDas, UndurtiPetersson, KristofferDasgupta, PiyaliPetiti, JessicaDasgupta, SantanuPetrik, JimDasgupta, SubhamoyPetrillo, AngelicaDash, Ranjeet PrasadPetrillo, SaraDaskalakis, KosmasPetriz, JordiDatta, ArpitaPetrulionis, MariusDaubon, ThomasPetta, IoannaDaudignon, AgnèsPettinato, GiuseppeDaugaard, MadsPeverali, FiorenzoDavalieva, KatarinaPeyrl, AndreasDavalos, AlbertPezone, AntonioDavatzikos, ChristosPezzella, FrancescoDave, SandeepPezzolo, AnnalisaDaverey, AmitaPezzuto, AldoDavid, AlexandrePfeffer, Lawrence M.David, MouraPfeffer, UlrichDavid, MutchPfeifer, AleksandraDavid-Cordonnier, Marie-hélènePfeiffer, PerDavie, JudithPflumio, FrançoiseDavis, JeremyPhan, Thanh KhaDavis, Richard E.Phan, Thi Tuong VyDavra, ViralkumarPhatarpekar, PrasadDawn Qingqing, ChongPhesse, TobyDawra, RajinderPhilipp, AlexanderDawson, ChristopherPiane, MariaDay, Chi-pingPiasecka, AgnieszkaDay, TerryPiazza, CesareDe Andrade, MarizaPicardi, MarcoDe Araujo, ElvinPiccaluga, Pier PaoloDe Benedetti, ArrigoPiccin, AndreaDe Biase, DarioPiccinin, ElenaDe Billy, EmmanuelPiccirillo, SaraDe Blank, Peter MatthewPiccoli, Giorgina BarbaraDe Bortoli, MichelePichi, BarbaraDe Bree, EelcoPichiorri, FlaviaDe Bree, RemcoPichler, MartinDe Cárcer, GuillermoPichon, MaximeDe Carvalho, Litia AlvesPichumani, KumarDe Castro, Gabriela S.Picimbon, Jean-FrançoisDe Cristofaro, TizianaPiciu, DoinaDe Falco, ValentinaPickard, AdamDe Felice, FrancescaPiekiełko-Witkowska, AgnieszkaDe Francesco, Ernestina MariannaPiemontese, SimonaDe Geus-Oei, Lioe-FeePierelli, LucaDe Gregorio, VincenzaPierga, Jean-YvesDe Groof, AdPieroni, LuisaDe Groot, Jan Willem B.Pierorazio, PhillipDe Haan, JaccoPietras, AlexanderDe Jong, Daphne D.Pietraszek-Gremplewicz, KatarzynaDe Keizer, Peter L.J.Pietro, Massimiliano DiDe Kruijff, Robin M.Pietrusiński, MichalDe La Peña, Francisco AyalaPiga, IsabellaDe La Puente, PilarPigazzi, Martinade la Roche, MarcPignatelli, DuarteDe La Rosa, JorgePilarsky, ChristianDe Langen, Adrianus J.Pileri, StefanoDe Lellis, LauraPillarisetty, Venu Gopalde Ligt, KellyPillon, MartaDe Los Reyes-Gavilán, Clara G.Pillozzi, SerenaDe Lourdes Pereira, MariaPilmane, MāraDe Luca, AngelaPiluso, GiulioDe Luca, Anna ChiaraPincus, SethDe Luca, AntonellaPineau, PascalDe Marchi, TommasoPineda, SilviaDe Martino, Maria CristinaPiñeiro, MartaDe Masi, GianlucaPiñeiro, RobertoDe Melo-Diogo, DuartePinker-Domenig, KatjaDe Miglio, Maria RosariaPinter, MatthiasDe Miguel, DiegoPinto, MafaldaDe Miguel-Pérez, DiegoPiovesan, AlessandroDe Miranda, NoelPiperi, ChristinaDe Nonneville, AlexandrePircher, AndreasDe Oliveira, Marcos RobertoPires, Bruno R. B.De Oliveira, Pedro ManuelPirich, ChristianDe Oliveira, SofiaPirola, LucianoDe Paoli, AntoninoPirooznia, MehdiDe Pauw, AntoinePirozzi, Christopher J.De Preter, KatleenPirro, StefanoDe Reijke, T.M.Pirtoli, LuigiDe Reijke, Theo M.Pisani, Carlade Reuver, Philip R.Pisapia, PasqualeDe Rienzo, AssuntaPiscaglia, FabioDe Robertis, MariangelaPiskin, SenolDe Robertis, RiccardoPitingolo, GabrieleDe Rosa, MarinaPittalà, ValeriaDe Rosa, VivianaPitts, Todd M.De Rossi, AnitaPiva, MarcoDe Sanctis, Juan BautistaPiva, RobertoDe Santo, CarmelaPiva, TerrenceDe Sepulveda, PauloPivarcsi, AndorDe Sousa E Melo, FelipePivetta, ElianaDe Sousa-Coelho, Ana LuísaPixley, FionaDe Toni, LucaPizzimenti, StefaniaDe Totero, DanielaPlanque, ChrisDe Veirman, KimPlatella, ChiaraDe Velasco, GuillermoPlatta, HaraldDe Vicente, Juan CarlosPlattner, RinaDe Vincentiis, MarcoPlesa, AdrianaDe Vita, AlessandroPletsch-Borba, LauraDe Wilt, Johannes H.W.Plonka, PrzemyslawDe Wit, RonaldPlotnikov, Egor Y.De, PradipPłuciennik, ElżbietaDeaglio, SilviaPluck, GrahamDean, MichaelPluijm, SaskiaDeandreis, DésiréePlymate, Stephen R.Deb, SubrataPlzak, JanDebeljak, NatašaPo, AgneseDecaestecker, ChristinePocard, MarcDeCaprio, JamesPodar, KlausDecaudin, DidierPodda, Alessandro SebastianDe-Chiara, LorettaPodhorecka, MonikaDeckert, MarcelPoeggeler, BurkhardDecmann, AbelPoggi, AlessandroDecristoforo, ClemensPoggio, FrancescaDe-Cubas, Aguirre A.Poglitsch, MarkoDedhar, ShoukatPohl, ChristianDedieu, StephanePohl, Sebastian Ӧther-GeeDeel, MichaelPoirier, DonaldDeevska, GerganaPoirier, Guy G.Defilippi, PaolaPoirier, YannickDefraene, GillesPoirot, MarcDegoul, FrançoisePojo, MartaDegrassi, FrancescaPóka, RóbertDehzangi, AbdollahPoku, Rosemary A.Dejardin, EmmanuelPolano, MaurizioDel Coco, LauraPolanska, AdrianaDel Prete, AnnalisaPolari, LauriDel Principe, Maria IlariaPolčic, PeterDel Rio, Maria-LuisaPoleszczuk, JanDel Rivero, JaydiraPoletajew, SlawomirDel Rosso, MarioPoli, AlessandroDel Sal, Giannino DelPoli, MauraDelbue, SerenaPoli, ValeriaDéleris, PaulPolimeno, LorenzoDelgado-Goni, TeresaPolishchuk, RomanDelk, Nikkí A.Polkowski, WojciechDell’Amore, AndreaPollack, Seth M.Dell’Aquila, EmanuelaPoller, DavidDella Pepa, Giuseppe MariaPollett, AaronDelle Monache, SimonaPollok, Karen E.Dellis, OlivierPolonis, VictoriaDelorme, StefanPonandai-Srinivasan, SakthiDelyon, JuliePonik, SuzanneDemetriades, Andreas K.Ponnambalam, VasDe-Miguel, ManuelPonnusamy, Thamarai (Suriyan)Demiot, ClairePonraj, PrabakaranDemir, Ihsan EkinPons, SebastianDeneka, AlexanderPontisso, PatriziaDeng, QuPonzetti, MarcoDeng, YoupingPonz-Sarvise, MarianoDenis, GeraldPooler, DarcyDenisov, EvgenyPoon, IvanDenoyelle, ChristophePop, LaurentiuDenoyer, DelphinePope, ChadDent, PaulPoplawski, PiotrDent, SusanPopp, HenningDeNunzio, Nicholas J.Popper, Helmut H.Denzel, Martin S.Pópulo, HelenaDeonarain, MahendraPorat, ZivDePamphilis, Melvin LPorcu, LucaDePaolo, WilliamPorro, ChiaraDepciuch, JoannaPorta, CamilloDepping, ReinhardPortella, GiuseppeDeptuła, MilenaPortman, NeilDer Wekken, Anthonie VanPorubský, StefanDeribe, Yonathan LissanuPosch, FlorianDerkay, CraigPosey, Avery D.Derks, J.L.Poškus, EligijusDerks, SarahPotenza, NicolettaDerwich, KatarzynaPothuraju, RameshDeryugina, Elena I.Potier-Cartereau, MarieDeryusheva, SvetlanaPotočnik, NejkaDesai, NiyatiPotratz, Jenny C.Desantis, VanessaPotschger, UlrikeDeshpande, ShayuPoulaki, VassilikiDesideri, IsaccoPouliakis, AbrahamDesiderio, VincenzoPouliot, FredericDesilets, AntoinePouliquen, Daniel LoïcDessein, Patrick H.Pouponnot, CelioDeuis, JenniferPoupot, MaryDeutsch, AlexanderPourbaghi Masouleh, MiladDeutz, NicolaasPourquier, PhilippeDevi, Gayathri R.Pouvesle, Jean MichelDevillier, RaynierPovero, DavideDevoogdt, NickPowell, ArfonDevred, FrançoisPowell, Simon N.Devy, JeromePowrózek, TomaszDewhirst, MarkPoyet, Jean-LucDey, KamolPozzo, CarmeloDey, ShuvashisPozzo, Eleonora DaDhakal, DipeshPozzo, FedericoDhasarathy, ArchanaPrabhu, Antony HeroldDhasmana, AnupamPradere, BenjaminDhawan, DeepikaPradet-Balade, BerengereDhodapkar, MadhavPradhan, Anjan KumarDhondt, BertPrakash, JaiDhoot, GurtejPramanik, ArindamDhungel, BijayPranjol, ZahidDi Agostino, SilviaPrante, OlafDi Baldassarre, AngelaPrasad, AnkushDi Bartolomeo, SabrinaPrasad, VikasDi Buduo, ChristianPrashar, SanjivDi Cataldo, AndreaPratap, JiteshDi Cesare Mannelli, LorenzoPreca, Bogdan TiberiusDi Cunto, FerdinandoPreda, LorenzoDi Franco, SimonePrehn, Jochen H. M.Di Girolamo, MariaPreibisch, ChristineDi Grezia, GraziellaPrencipe, MariaDi Lorenzo, GiuseppePrentice, PaulDi Maio, MassimoPresneau, NadegeDi Marcotullio, LuciaPreti, MarioDi Martino, EricaPrevet, NellaDi Matteo, SabinaPrévostel, CorinneDi Micco, PierpaoloPrice, StephenDi Micco, RosaPriceman, Saul J.Di Nicola, MassimoPricl, SabrinaDi Pace, Anna LauraPrimig, MichaelDi Paolo, AntonelloPrince, MilesDi Renzo, LiviaPrincipe, Daniel R.Di Rocco, GiulianaPrincipe, Maria Ilaria DelDi Stasi, AntonioPrincipia, Scavo MariaDi Zazzo, ErikaPrinz, FelixDi. Tommaso, LucaPrivette Vinnedge, LisaDiamond, Jennifer R.Probst, PascalDias, FranciscaProcino, GiuseppeDiaz, AbbeyProescholdt, MartinDíaz, JoséProsperi, EnnioDíaz-Carballo, DavidProsperi, JeniferDíaz-Peña, RobertoProsperi, Jenifer R.Díaz-Rodríguez, ElenaProvot, SylvainDickson, Paxton V.Przemek, KrawczykDidier, ChristinePrzybyło, MałgorzataDidilescu, AndreeaPrzygodzka, PatrycjaDiederich, Chris J.Pucino, ValentinaDiederich, MarcPuglisi, FabioDiederichs, SvenPuhka, MaijaDiepstra, ArjanPuig Morón, NoemíDiesch, JeanninePujana, Miquel ÀngelDietrich, CharlesPula, BartoszDietrich, OlafPulford, KarenDietrich, WalshPulito, ClaudioDigiacomo, GrazianaPuliyappadamba, Vinesh ThidilDijk, FrederikePulliero, AlessandraDillman, Robert O.Pulverer, WalterDiMattia, GabrielPurcell, AnthonyDimitrakopoulos, Foteinos-IoannisPuri, SonamDimitrakopoulou-Strauss, AntoniaPusch, StefanDimmock, Jonathan R.Pützer, Brigitte M.Dimri, ManaliPuxeddu, RobertoDing, JianxunPylayeva-Gupta, YuliyaDing, YapingPyronnet, StéphaneDini, LucianaQian, ChunqiDini, ValentinaQian, JinDinic, JelenaQian, WeiDiniz, CarmenQian, YanrongDinulescu, Daniela M.Qiao, HuanyuDiogo, PatriciaQin, QianDionisi, FrancescoQin, ZhiqiangDistel, LuitpoldQiu, XinDistel, MartinQiu, YiDittmar, ThomasQiu, ZhenDittmer, JurgenQu, NingDivella, RosaQu, PengDjerada, ZoubirQuadri, SyedDo, Minh TruongQuagliariello, VincenzoDobos, GaborQuaia, EmilioDobosz, BernadetaQuaini, FedericoDobrescu, AmadeusQuanico, JusalDobrovic, AlexanderQuaquarini, EricaDobrowolski, PiotrQuarta, AlessandraDobruch, JakubQuax, Wim J.Dodd, Rebecca D.Que, JennyDodi, GianinaQuehenberger, FranzDoglietto, FrancescoQueiroga, FelisbinaDoglio, AlainQuemeneur, EricDogra, PranayQuerleu, DenisDogra, PrashantQuero, GiuseppeDogra, SamritaQuesnel-Vallières, MathieuDoh, Kyung-OhQuinn, Gwendolyn P.Dohan, AnthonyQuiros-Gonzalez, IsabelDokudovskaya, SvetlanaR.Van Bockstal, MiekeDoll, Jennifer A.Rabien, AnjaDomagala-Kulawik, JoannaRacanelli, VitoDomen, AndreasRachiglio, AmDomenech, MaribellaRacioppi, MarcoDomenech, MartaRadchenko, Eugene V.Domenichini, AliceRaderer, MarkusDomingo, EnricRades, DirkDomínguez-Valentín, MevRadice, PaoloDonadon, MatteoRadisavljevic, ZivDonati, BenedettaRadogna, FlaviaDonato, Rosario FrancescoRadu, Caius G.Dondi, GiuliaRaduly, Lajos ZsoltDondossola, EleonoraRadvanyi, FrancoisDong, JixinRae, ColinDong, QinglinRafael, DianaDong, ShenRafaelsen, Søren RafaelDonia, MarcoRafei, MoutihDonizetti, AldoRaffa, SalvatoreDonnelly, DeirdreRaffenne, JeromeDonnelly, LouiseRaffetseder, UteDonnelly, OliverRaffone, AntonioDonneyong, Macarius M.Raghavan, ShreyaDonninger, HowardRaghavan, SudhirDorfer, ChristianRagin, CamilleDormond, OlivierRagni, EnricoDörrie, JanRagusa, Maria AntoniettaDos Anjos Pires, MariaRahbari, Nuh N.Dos Santos, Nuno RodriguesRahib, LolaDosquet, ChristineRahikkala, AnttiDosset, MegalieRahman, ArmanDotan, ZoharRahman, RumanDott, Giovanni MarascoRahme, GilbertDougan, Stephanie K.Rai, AlinDouglas, Steven D.Rai, AnkitDoyle, Gerald V.Rai, SumitDragomir, MihneaRai, VikrantDrake, Penelope M.Raikar, Sunil S.Drakes, Maureen L.Raimondi, LaviniaDrakos, EliasRaimondo, DiegoDrews, UlrichRaimondo, FrancescaDrexler, Hans G.Raimondo, StefaniaDrissen, RoyRaingeaud, JoëlDroz, JeanpierreRaj, NitinDu, XingrongRajagopalan, AnugrahaDu, XuexiangRajarathnam, KrishnaDuan, WeiRajasekaran, NarendiranDuarte Campos, Daniela F.Rajendran, PraveenDubash, Taronish DorabRajpert-De Meyts, EwaDubeau, L.Rajpurohit, SurendraDubey, HarishchandraRak, JanuszDubey, RaminRakic, JelenaDubois, ChristopheRakislova, NataliaDubrana, KarineRakus, DariuszDubrova, YuriRamachandiran, IyappanDubrovska, AnnaRamaiahgari, Sreenivasa C.Duchi, SerenaRamakrishnan, VijayDudas, JozsefRamakrishnan-Geethakumari, PraveenDuechler, MarkusRamalingam, NaveenDuez, PierreRamaraj, PanduranganDufresne, ArmelleRamasamy, MohankandhasamyDugo, MatteoRamaswamy, VijayDugué, Pierre-AntoineRambaldi, AlessandroDuhamel, MarieRambow, FlorianDulskas, AudriusRamchandani, DivyaDumas, M. Jean-FrancoisRamella, SaraDumaz, NicolasRamello, Maria CeciliaDumond, HeleneRamić, SnježanaDumont, FrédéricRamírez, ManuelDumontet, CharlesRamirez, Robert A.Dun, Matthew D.Ramirez-Peña, EsmeraldaDuncan, SteveRamon Torrell, Josep MariaDuncombe, AndrewRamont, LaurentDunkel, Ira J.Rampazzo, ElenaDunlop, ElaineRampias, TheodorosDunne, RichardRamsay, AlanDupéré-Richer, DaphnéRan, SophiaDuployez, NicolasRana, AjayDupré, AurélienRana, BasabiDupré, Denis J.Randy, JensenDurand, Jean-OlivierRanganna, KasturiDurand, NishaRanieri, GirolamoDuraturo, FrancescaRanjan, AtulDurocher, FrancineRanjan, KishuDutil, JulieRansac, StéphaneDutreix, MarieRanzato, EliaDutta, ArijitRao, MahadevDutta, PalashRao, SheelaDutta, PranabanandaRao, Srinivasa RaoDutta, SamikshanRao, VidhyaDüzgünes, NejatRaoul, WilliamDwyer, Johanna T.Rapak, AndrzejDwyer-Nield, Lori D.Raphael, JacquesDy, GraceRapic, SaraDye, Danielle E.Rasche, LeoDykhuizen, EmilyRasmussen, LeneDziedziejko, ViolettaRaspadori, DonatellaDziegiel, PiotrRaspagliesi, FrancescoDzięgiel, PiotrRassweiler, Jens J.Dziegielewska, BarbaraRastegar, MojganDziegielewski, Peter T.Ratajewski, MarcinDzobo, KevinRather, GulamE Quelle, DawnRattan, RamandeepEar, JasonRattay, KristinEarl, JulieRatti, StefanoEberhart, CharlesRau, BeateEbihara, LisaRau, Tilman TEble, JohannesRaucher, DrazenEchchannaoui, HakimRaugei, GiovanniEchevarría, EnriqueRauh, MichaelEck, Ruben J.Raundhal, MaheshEckerdt, Frank D.Raup-Konsavage, WesleyEckert, FranziskaRausei, StefanoEckert, Mark A.Rava, MartaEckschlager, TomasRavegnini, GloriaEconomopoulou, PanagiotaRavn-Haren, GitteEdeline, JulienRawicz-Pruszyński, KarolEden, AmirRay, Heather J.Edhi, Muhammad MuzzammilRay, ParthaEdiriweera, Meran KeshawaRay, SutapaEdward Lawler, SeanRayar, MichelEdwards, ClaireRazvan-Cosmin, PetcaEferl, RobertRazzokov, JamoliddinEfimova, TatianaRea, IlariaEfremov, Dimitar GReader, Jocelyn C.Egea, JavierReagan, John L.Egger, GerdaRebelo, SandraEggert, DennisRebmann, VeraEguchi, TakanoriRecasens, AriadnaEhata, ShogoRECHER, ChristianEhlers, MargretReddy, SakamuriEhrsson, Ylva TiblomReddy, Sudhir PuttyEiber, MatthiasRedell, MicheleEichbaum, MichaelRedmer, TorbenEichelmann, Ann-KathrinRedondo-Muñoz, JavierEichenauer, Dennis A.Reduzzi, CarolinaEichhoff, Ossia M.Reece, JeanetteEichhorn, Martin E.Reed, Amy McCartEichhorn, PieterReeh, MatthiasEichler, MartinReems, Jo AnnaEid, Ali HusseinRegenbrecht, ChristianEikesdal, Hans PetterRehfeld, Jens FrederikEisenstat, David D.Reich, OlafEisinger-Mathason, Tzipora S.KarinReimer, ToralfEiz-Vesper, BrittaReinartz, SilkeEkert, PaulReindl, JudithEl Bairi, KhalidReindl, KatieEl Demellawy, DinaReiner, Anne S.El Desoky, Ehab S.Reis, HenningEl Shamieh, SaidReissfelder, ChristophElas, MartynaReissmann, SiegmundEl-Ashry, DorrayaReiter, MichaelElble, Randolph C.Religa, PiotrElezkurtaj, SeferRemaut, KatrienEl-Gewely, Mohamed RaafatRemberger, MatsEl-Heliebi, AminRemick, JillEliçin, OlgunRemmenga, Steven W.Eliseeva, IrinaRen, BinEl-Jawhari, JehanRen, ZhenhuaElkabets, MosheRenard, Pierre-YvesElkhadragy, LobnaRenner, WilfriedEl-Khoury, VictoriaRenò, FilippoEllinger, JörgRenwick, NeilElmouki, IliasRenzulli, MatteoEl-Naggar, AdelRepasky, ElizabethEloranta, SandraRepetto, OmbrettaElsayad, KhaledRequena, DavidElvas, FilipeReshkin, Stephan JoelElyada, ElaRestaino, StefanoEmadali, AnoukReszka, EdytaEmeto, Theophilus I.Retière, ChristelleEmir, Uzay E.Rettig, EleniEmons, GünterRettinger, EvaEmran, Abdullah AlReuben, AlexandreEndersby, RaeleneReunanen, JustusEndo, MakotoReuther, GaryEng, CathyRevuri, VishnuEngedal, Kim NikolaiReymond, MarcEngel, RebekahReyners, Anna K.L.Engeland, Christine E.Rhett, Joshua MatthewEngelhardt, MonikaRho, Jin KyungEngels, YvonneRhodes, Lyndsay V.Engevik, MelindaRi, MasakiEngidawork, EphremRiabowol, KarlEngle, JonathanRiar, Amanjot KaurEnjeti, AnoopRibaldone, Davide GiuseppeEnomoto, AtsushiRibaudo, GiovanniEnomoto, MasaruRibera, JordiEnrico, MaffiniRibera, Jose MaríaEntz-Werle, NatachaRibero, SimoneEoli, MaricaRicardo, SaraEom, Joo BeomRiccardo, FedericaEppard, ElisabethRicchiuto, PieroEppenberger-Castori, SerenellaRicci, AlbertoEpperla, NarendranathRicci, Angela DaliaEppler, ElisabethRicci, FrancescaEpstein, Alan L.Ricci, GiuliaEpstein, OwenRicci, GiuseppeErben, PhilippRicciardelli, CarmelaErber, RamonaRicciardi, Maria RosariaEri, S. SrivatsanRichardson, Debra L.Eric Kast, RichardRiches, John C.Eric Pasmant, EricRichter, Günther H.S.Erkmen, Cherie P.Richter, JoshuaErnani, ViniciusRichter, SusanEROVIC, Boban M.Richtig, GeorgErreni, MarcoRicketts, Christopher J.Erson-Omay, Emine ZeynepRider, CynthiaErtaylan, GokhanRidola, LorenzoEscande, AlexandreRieckmann, ThorstenEscargueil, AlexandreRiediger, ThomasEscors, DavidRieken, StefanEskazan, Ahmet EmreRiekstiņa, UnaEso, YujiRiemann, DagmarEspel, EnricRiesco-Eizaguirre, GarcilasoEspinoza, Jaime A.Riesterer, OliverEsposito, FrancescoRigalli, Juan PabloEsposito, GabriellaRigaud, MichelEsposito, Maria TeresaRigby, Matthew H.Esposito, Maria ValeriaRighi, LuisellaEssani, KarimRiis, Margit L.H.Estelrich, JoanRimassa, LorenzaEsteve-Puig, RosauraRinaldi, AndreaEstévez, Laura GarcíaRinaldi, LucaEsworthy, Robert StevenRingdén, OlleEtrych, TomášRink, AndreasEu, PeterRinner, BeateEufemi, MargheritaRinnerthaler, GabrielÉva, MikóRiobo, NataliaEvangelisti, CamillaRiond, JoëlleEvani, Shankar JaikishanRiquelme, ErickEvans, D. GarethRishi, ArunEvans, MichaelRissiek, BjoernEvans, MyronRitsma, LailaEvanson, Nathan K.Ritter, AndreasEvert, MatthiasRiva, GiuseppeEvrard, Solène M.Rivera, FranciscoEykyn, ThomasRivero, FranciscoEzekiel, UthayashankerRivero-Müller, AdolfoEzzat, ShereenRizos, HelenFabbrini, Maria SerenaRizvi, ImranFaber, Anthony C.Rizvi, Syed A.A.Fabian, Ido DidiRizzi, ManuelaFabien, ForestRizzino, AngieFabijańska, MałgorzataRizzo, AlessandroFabisiewicz, AnnaRizzo, FrancescaFabris, LindaRizzo, StefaniaFabris, LucaRizzolio, FlavioFabrizio, Federico PioRobak, TadeuszFacchiano, AntonioRobaszkiewicz, AgnieszkaFacchinetti, FrancescoRobe, Pierre A.Facoetti, AngelicaRoberg, KarinFaden, DanielRobert, LidiaFaehling, MartinRoberts, David D.Faggioni, LorenzoRoberts, TaraFagioli, FrancaRobila, Valentina V.Fagoonee, SharmilaRobin, Jérôme D.Failla, Cristina M.Robinson, NirmalFaille, DorothéeRobinson, StevenFais, StefanoRobinson, Timothy J.Faísca, PedroRobles, AnaFalchetti, Maria LauraRoby, KatherineFalcioni, RitaRocca, MicheleFalcone, ItaliaRoccaro, AldoFalk, StephenRocco, GaetanoFalzone, LucaRocha, SoniaFan, DapingRochel, NatachaFan, MeiyunRochigneux, PhilippeFanale, DanieleRodat-Despoix, LiseFanciulli, GiuseppeRoderburg, ChristophFandrey, JoachimRodilla, VerónicaFane, MitchellRodin, Andrei S.Fanetti, GiuseppeRodolfo, CarloFanfani, FrancescoRodolfo, MonicaFang, JenniferRodrigo, Juan P.Fang, JunRodrigo, LuisFang, MingzhuRodrigues Teixeira, Manuel AntónioFang, ShengyunRodrigues, António SebastiãoFang, XianjunRodrigues, DarioFanizzi, AnnaritaRodrigues, Manuel JorgeFankhauser, Christian.Daniel.Rodrigues, Pedro MiguelFanourakis, GalinosRodriguez, CristianFantini, Massimo C.Rodriguez, F. DavidFantini, SebastianRodriguez, Gloria MarcelaFanzani, AlessandroRodríguez, JavierFarace, FrançoiseRodriguez, ReneFaraji, FarhoudRodríguez-Antona, CristinaFaraoni, IsabellaRodríguez-Ariza, AntonioFarge, GéraldineRodriguez-Flores, JuanFarina, LorenzoRodríguez-García, CarmenFariña-Sarasqueta, ArantzaRodriguez-Lafrasse, ClaireFarlik, MatthiasRodríguez-Mier, PabloFarolfi, AlbertoRodríguez-Milla, Miguel ÁngelFarr, Christine J.Rodríguez-Perálvarez, ManuelFarrell, JohnRodriguez-Vargas, Jose M.Farrell, RobertRodríguez-Vita, JuanFassl, AnneRodríguez-Yoldi, María JesúsFast, Loren D.Roedel, FranzFatima, IramRoeder, FalkFato, RomanaRoeker, LindseyFattizzo, BrunoRoelofs, JeroenFattore, LuigiRoemer, Margaretha G.M.Faucz, Fabio R.Roex, GilsFaulkner, SamRogatto, SilviaFaundez, VictorRogers, Jane E.Fava, CarmenRogers, JimFava, PaoloRogers, KerryFavia, GianfrancoRogers, Natasha M.Faviana, PinucciaRoghanian, AliFavre, ClaudioRogister, BernardFazal, ZeeshanRognoni, EmanuelFazio, Vito MicheleRoh, Ju-WonFeczko, TivadarRohr-Udilova, NataliyaFedele, MonicaRojiani, Mumtaz V.Feferman, TaliRojo, FedericoFehr, AndréRojo, Maria AngelesFei, PeiwenRokavec, MatjazFeichtinger, Rene GRolla, SimonaFeijs, Karla L.H.Romano, AlessandraFelicetti, FedericaRomano, AndreaFeliu, JaimeRomano, GabrieleFelli, Maria PiaRomanov, VictorFend, FalkoRomão, LuísaFendler, Wojciech M.Roma-Rodrigues, CatarinaFeng, HuiRombouts, KristaFeng, QiangRomei, CristinaFeng, WanjuanRomero, Marta R.Feng, YibinRóna, GergelyFeng, YinnianRonca, RobertoFens, Marcel H.A.M.Ronchetti, DomenicaFenton, TimRonchi, AndreaFeo, ClaudioRoncucci, LucaFeo, FrancescoRondon, AurélieFerbeyre, GerardoRonellenfitsch, UlrichFereidouni, FarzadRong, ChaoFernandes, BrunoRonot, MaximeFernandes, RúbenRonsard, LaranceFernández De Mattos, SilviaRood, Brian R.Fernandez, Agustin F.Rookus, MattiFernandez, Hugo F.Rooney, ClaireFernandez-Botran, RafaelRoos, DanielFernández-Lázaro, DiegoRoos, Wynand P.Fernández-Santander, AnaRoque, AliciaFernández-Silva, PatricioRoque, NataliaFeron, OlivierRosa, DivellaFerracini, RiccardoRosado, JuanFerrand, François-RégisRosanò, LauraFerrantelli, FlaviaRosato, AntonioFerrara, BenedettaRoschke, AnnaFerrari, DarisRose, April A.N.Ferrari, PaolaRose, MichaelFerrari, Silvia MartinaRose-John, StefanFerraro, SimonaRosell, RafaelFerre, FrancescaRoselli, MarioFerreira, FernandoRosen, KirillFerreira, Leonardo M.R.Rosenbluh, Joseph SefiFerreira, PedroRosencrantz, Ruben R.Ferrer, GerardoRosenquist, RichardFerretti, ElisabettaRosenzweig, Steven A.Ferroni, PatriziaRosière, RémiFerrucci, Pier FrancescoRosol, ThomasFesta, FernandaRoss-Adams, HelenFestuccia, ClaudioRossi, ElenaFeugeas, Jean-PaulRossi, GiulioFezza, FilomenaRossi, Noreen F.Fierstra, JornRossi, PaoloFigg, William D.Rossi, RiccardoFigini, MariangelaRossi, Roberta ElisaFigueiredo, JoanaRössler, KarlFigueroa, AngélicaRosso, Mario DelFilho, Julio RicarteRossowska, JoannaFilić, VedranaRoszik, JasonFilipeanu, CatalinRota, RossellaFilippo, AlongiRotblat, BarakFilippova, NataliaRothschild, Sacha I.Finco, IsabellaRotondo, John CahrlesFinetti, FedericaRotrekl, VladimírFinger, Paul T.Rotte, AnandFinkielstein, CarlaROUE, GaelFinocchiaro, GaetanoRouleau, EtienneFiocchetti, MarcoRoulois, DavidFior, RitaRoumiguie, MathieuFiore, DaniloRousseau, AudreyFioretti, DanielaRousseau, CarolineFiori, EnricoRoussel, MikaëlFirczuk, MalgorzataRoviello, GiandomenicoFirestein, RonRovini, AmandineFirlak, MelikeRovirosa, ÁngelesFischer, JesseRovito, Michael J.Fischer, Nicholas W.Rovo, AliciaFischer, NicoleRowther, FarjanaFishbein, LaurenRoy, DhruvajyotiFisher, PaulRual, Jean-FrançoisFitzgerald, DavidRübenthaler, JohannesFitzGerald, LieselRubert, JosepFiume, GiuseppeRubina, KseniyaFiz, FrancescoRubinek, TamiFlavell, RobertRubis, BlazejFlavin, RichardRucci, NadiaFleischman, Angela G.Ruch, RandallFleury, FabriceRuchała, MarekFliedner, StephanieRückert, FelixFlorea, AdrianRudd, SeanFlorea, Ana-MariaRudel, Ruth AnnFlorian, Maria CarolinaRudolf, EmilFlorio, TullioRudolph, ChristianFocosi, DanieleRudra, PratyaydiptaFolini, MarcoRueda, BoFong, ChunRueda, Bo RubenFontenay, MichaelaRuess, Dietrich A.Fonti, RosaRui, LixinForest, FabienRuiz Pérez, José F.Forghieri, FabioRuiz Pérez, María VictoriaForma, EwaRuiz, AnaFormisano, PietroRuiz, Irene RiusFornaguera, CristinaRuiz-Casado, AnaForrester, HelenRuiz-Echevarria, Maria J.Förster, RobertRuiz-Martínez, SantiagoFort, PhilippeRund, DeborahForte, DorianRus, GuillermoForte, Iris MariaRusan, MariaFortunato, OrazioRusanova, IrynaFoschini, M.P.Russ, BrendanFossati, PieroRussell, JamesFossmark, ReidarRussell, StepehenFossum, EvenRusso Krauss, IreneFoster, Jennifer H.Russo, AlessandroFoster, Paul A.Russo, AntonioFotiadis, Dimitrios I.Russo, DanielaFotouhi, OmidRusso, DomenicoFoukakis, TheodorosRusso, GiovannaFountzilas, ChristosRusso, Matteo A.Fournel, LudovicRusso, SuzanneFournier, Marcia V.Russu, WadeFracchiolla, Nicola StefanoRustemi, OrielaFradin, DelphineRuszkowska-Ciastek, BarbaraFragliasso, ValentinaRutegård, MartinFraineau, SylvainRutenberg, Michael S.Fraizer, GailRutherford, MarkFrampton, Adam E.Rutkowsk, PiotrFrancesca, LunardiRuzgys, PauliusFrancesca, OrsoRuzzolini, JessicaFranceschini, GianlucaRwigema, Jean-Claude M.Franchet, CamilleRyan, AideenFranchi, MatteoRychahou, PiotrFranchini, Don-MarcRydén, LisaFrancia, GiulioRyszawy, DamianFrancini, EdoardoRyu (Yoo), Kyung HyunFrancipane, Maria GiovannaRyu, Han SukFrancis, Gary L.Rzymowska, JolantaFrancis, JasmineSaad, FredFranco, LuisSaad, MohamedFranco, Marina SantiagoSaba, Nakhle S.Franco, OmarSabaratnam, RugivanFranco, PierfrancescoSabarimurugan, ShanthiFranco-Barraza, JanuszSabater, SebastiàFrancuz, TomaszSabatier, LaureFrandsen, Stine KrogSabatier, RenaudFrapolli, RobertaSabatino, GiovanniFraser, MichaelSabatino, LauraFrati, AlessandroSabbioneda, SimoneFrazzi, RaffaeleSabol, MajaFredsøe, JacobSaborowski, AnnaFreeman, MichaelSabri, SihamFregnan, FedericaSacchetti, BenedettoFreiberger, Sandra NicoleSaceda, MiguelFreitas, RenataSachpekidis, ChristosFrench, DavidSachs, PatrickFrench, Pim J.Sachsenmeier, KrisFrenkel-Morgenstern, MilanaSack, UlrichFrère, CorinneSadasivam, MohanrajFreund, Jean NoelSadeghi, SaeedFrew, IanSadik, RiadhFrey, Melissa K.Sadri-Ardekani, HoomanFreyer, GillesSaenko, VladimirFrezzato, FedericaSaez, CarmenFribley, AndrewSagar, VinayFrieboes, HermannSagara, AtsunobuFriedmann Angeli, José PedroSaha, AchintoFries, Jochen W.U.Saha, DipongkorFriess, HelmutSaha, NirmalyaFriis-Hansen, LennartSaha, RajibFriocourt, GaelleSahin, OzgurFrisan, TheresaSahni, SumitFrisch, KimSahoo, SubhransuFrisch, Steven M.Sahu, Ravi P.Fritah, SabrinaSaiag, PhilippeFrittitta, LuciaSaid, NeveenFrizelle, FrankSaidu, Nathaniel Edward BennettFröhlich, EleonoreSaini, SharanjotFröhlich, HolgerSaintigny, PierreFrohme, MarcusSaito, HirotakeFromigué, OliviaSaito, RyokoFromm, BastianSaito, RyutaFromont, GaelleSaito, YukiFruci, DorianaSaitoh, ManabuFruscio, RobertSaitoh, MasaoFu, Hua-WenSaitoh, TakayukiFuchs, Florian SiegfriedSakai, MakotoFuchs, HendrikSakai, OsamuFuchs, OtaSakamoto, Kathleen M.Fuegger, ReinholdSakamoto, NaoyaFuentes-Mattei, EnriqueSakamoto, ShinichiFugazzola, LauraSakata, JunkiFujihara, HisakoSaki, NajmaldinFujii, KentaroSakoda, Lori C.Fujii, Shin-ichiroSakuma, KunihiroFujii, TomomiSakurai, HiroyukiFujimoto, JunyaSakurai, YuFujimura, AtsushiSakwe, AmosFujimura, TakuSaladi, Srinivas VinodFujisawa, YasuhiroSałaga, MaciejFujishima, FumiyoshiSalar, AntonioFujita, KaoriSalati, MassimilianoFujita, KazutoshiSalazar, IvanFujita, MasatoshiSalciccia, StefanoFujita, MitsuguSaldanha, SabitaFujita, YukiSaleeb, RolaFujiwara, NaotoSaleh, TareqFujiwara, NatsumiSalehzadeh-Yazdi, AliFukai, JunyaSalem, AbdelhakimFukayama, MasashiSalerno, SergioFukuda, MinoruSalgado, RobertoFukuoka, HidenoriSalih, Helmut RainerFukusato, ToshioSalit, Irving E.Fukushima, HiroshiSalmasi, AmiraliFukushima, SatoshiSalomaa, SiskoFukushima, ToshiakiSaltarella, IlariaFukushima, TsuyoshiSalvati, EricaFulci, ValerioSalvatore, FrancescoFulciniti, FrancoSalvatore, LisaFuller, PeterSalvatori, PietroFumagalli, CaterinaSalvi, MauroFumoto, ShintaroSalvia, RobertoFunahashi, YasuhiroSalvucci, ManuelaFunai, KazuhitoSamaja, MicheleFunamizu, NaotakeSamaniego, RafaelFunaro, AdaSamara, PinelopiFunel, NiccolaSambandam, VaishnaviFüredi, AndrásSambri, AndreaFurlanetto, JennySamimi, MAHTABFurler, Robert L.Sammartino, PaoloFurman, Wayne L.Samnick, SamuelFurney, Simon J.Sampaolo, SimoneFurtner-Srajer, JuliaSampetrean, OlteaFurukawa, TatsuhikoSamson, AdelFurusawa, YukihiroSamuel, TemesgenFuruta, SaoriSanavia, TizianaFuruta, TakuyaSanche, LeonFusco, AlfredoSánchez, María-JoséFusco, NicolaSánchez-Bueno, FranciscoFusetti, StefanoSánchez-Quesada, CristinaFushida, SachioSancisi, ValentinaFutakuchi, MitsuruSanda, MiloslavGaballo, AntonioSander, VeronikaGabriel, EmmanuelSanderson, ChrisGabrielli, BrianSanderson, PriscillaGabriely, GalinaSandhu, Shahneen K.Gackowski, DanielSandini, MartaGaczynska, MariaSandomenico, FabioGad, Annica K. B.Sandulache, VladGadad, ShrikanthSanduleanu, SilviaGaddameedhi, ShobhanSanford, Nina N.Gagat, MaciejSanjeevaiah, AravindGage, JuliaSano, DaisukeGagliano, TeresaSanseviero, EmilioGagos, SarantisSansone, PasqualeGaida, MatthiasSantamaria, DavidGaidano, ValentinaSantamaria, RitaGaipl, Udo S.Santamaria-Martínez, AlbertGaiser, TimoSantambrogio, PaoloGaitskell, KeziaSantaolalla, AidaGajewska, A.Santarelli, AndreaGajos-Michniewicz, AnnaSanti, MariaritaGalandrini, RicciardaSantolla, Maria FrancescaGalardi, SilviaSantoni, GiorgioGalardy, Paul J.Santos-Rocha, Rita AlexandraGalasso, GiovanniSantulli, GaetanoGalateau Salle, FrançoiseSanz, Miguel A.Galbiati, SilviaSanz, RebecaGalea, MarySaoudi, AbdelhadiGaletta, DomenicoSapalidis, KonstantinosGaletti, MariclaSapino, AnnaGalgani, MarioSapino, SimonaGali, HimabinduSaponaro, ConcettaGalileo, Deni S.Sapozhnikov, Alexander M.Gall Troselj, KoraljkaSaretzki, GabrieleGallardo, EnriqueSarhan, DhifafGalle, JoergSarkar, ChandraniGallego, Óscar S.Sarkar, Navonil DeGallerani, GiuliaSarkar, SibajiGalli, AlavaroSarkar, SusobhanGalli, RossellaSarkaria, JannGallinella, GiorgioSarkozy, ClémentineGallipoli, PaoloSarma, PranjalGallo, AlessiaSarma, SauravGallo, DanielaSarmiento Soto, ManuelGallo, OresteSarna, MichalGallotta, ValerioSartini, DavideGallyas, FerencSartore-Bianchi, AndreaGalvano, AntonioSasabe, EriGaly-Fauroux, IsabelleSasada, TetsuroGambacorta, Maria AntoniettaSasaki, HirokiGAMBART, MarionSasaki, MakotoGamberi, TaniaSasaki, MotokoGanci, FedericaSasaki, RyuGandhi, Maher K.Sasaki, TakeshiGandhi, Om P.Sasaki, YasushiGandhirajan, Rajesh KumarSase, KazuhiroGandini, SaraSastre, LeandroGanesh Rai, BantukalluSastre-Serra, JorgeGaneshan, DhakshinamoorthySato, AkinoriGanguly, AnutoshSato, FuyukiGanguly, AvishekSato, HiromiGanini, CarloSato, KazuhideGao, DianSato, ShuichiGao, JieSato, TakamiGao, Rebecca W.Sato, YasutoGao, ShuaiSato, YukiyasuGarattini, EnricoSatoh, TarohGaravaglia, SilviaSatoi, SoheiGaravelli, SilviaSatoshi Kashiwagi, SatoshiGarbar, ChristianSaturnino, CarmelaGarbers, ChristophSaule, SimonGarbis, SpirosSaulnier, RonGarcía Gonzalo, FrancescSaunders, PhilippaGarcía Gutiérrez, José ValentínSaurin, AdrianGarcia, Jose MSaus, EsterGarcía-Aranda, MarilinaSavaskan, Nicolai E.Garcia-Borron, Jose CarlosSavino, BenedettaGarcía-Castro, JavierSavion, NaphtaliGarcía-Escudero, RamónSavoia, PaolaGarcía-Otín, Ángel LuisSawyer, Elinor J.Garcia-Pardo, JavierSaxena, SugandhaGarcia-Rico, EduardoSayans, Mario PerezGarcía-Silva, SusanaSaydam, OkayGarí, EloiSayner, Sarah L.Gariboldi, ManuelaScafoglio, ClaudioGariglio, MarisaScala, StefaniaGarje, RohanScanu, Antonio MarioGarlipp, BenjaminScarlett, GarryGarmy-Susini, BarbaraScarpa, Emanuele-SalvatoreGarofalo, CeciliaScarpa, MelaniaGarofalo, MariangelaScarpa, SusannaGarrastachu, PuyScatena, RobertoGartner, FátimaSchaefer, BenediktGartrell, Robyn D.Schäfer, ReinholdGarty, GuySchaft, NielsGarvalov, Boyan K.Schaschke, NorbertGarzon, SimoneSchatz, Jonathan H.Gasana, JanvierScheijen, BlancaGasche, ChristophScheinberg, David A.Gąsior-Perczak, DanutaScheiner, BernhardGaspari, MarcoScheithauer, WernerGasparian, MarineSchell, MichaelGaspar-Maia, AlexandreSchenk, MirjamGasparri, RobertoSchepisi, GiuseppeGaston, KevinScherbakov, Alexander M.Gately, KathyScherer, DominiqueGatselis, NikolaosSchettini, FrancescoGatta, ValentinaSchettino, GiuseppeGatti, LauraSchewe, DenisGatto, LidiaSchiavoni, GiovannaGaur, NishthaSchicho, RudolfGautam, PrsonSchick, Joel A.Gautier, MathieuSchiergens, Tobias S.Gavert, NancySchildgen, VerenaGavriilaki, EleniSchilling, BastianGawel, DamianSchindl, RainerGay, FrancescaSchininà, VincenzoGaze, Mark N.Schipmann, StephanieGazeli, KristaqSchirren, RebekkaGazouli, MariaSchirrmacher, RalfGbolahan, OlumideSchjesvold, FredrikGe, YubinSchlaepfer, IsabelGe, ZongYuanSchlaepfer, Isabel R.Geara, Fady B.Schleicher, Sabine B.Gebauer, FlorianSchlesinger, MartinGebauer, NiklasSchlisio, SusanneGee, KatrinaSchlumbrecht, MatthewGeha, SamehSchmelz, Eva M.Gehl, Karen JulieSchmetzer, Helga MariaGeisberger, RolandSchmid, AndreasGeissler, KlausSchmid, RalphGélébart, PascalSchmidt, BarbaraGeletneky, KarstenSchmidt, ManfredGeller, MelissaSchmidt, MarcinGELMINI, RobertaSchmidt, Mirko H.H.Gelsomino, FabioSchmidt-Hegemann, Nina-SophieGelsomino, FrancescoSchmidtke, GunterGener, PetraSchmidt-Wolf, Ingo Gh H.Geng, FengSchmitt, FernandoGennari, AlessandraSchmitt, MichaelGentile, FrancescoSchmitz, John C.Gentile, SaverioSchneider, BarbaraGentilini, Oreste D.Schneider, BjoernGenuardi, ElisaSchneider, Marc A.Geoffrois, LionnelSchneider-Stock, RegineGeorg, DietmarSchnütgen, FrankGeorgakilas, AlexandrosSchoenhagen, PaulGeorge, SophiaSchoffer, OlafGEORGES, StevenSchölch, SebastianGeorgopoulos, NikolaosSchönberger, StefanGeraldo Yoneyama, Kelly AparecidaSchönthal, Axel H.Gerbes, AlexanderSchrader, JörgGerke, OkeSchraml, PeterGerloff, DennisSchrank, Travis P.Gerrard, GarethSchreck, Karisa C.Gerratana, LorenzoSchreiber, MartinGershon, TimothySchröder, ReinhardGessani, SandraSchueler, JuliaGessi, StefaniaSchuetze, Scott M.Gessler, FlorianSchuler, PatrickGessner, GuidoSchulman, SamGeuna, MassimoSchulz, Wolfgang A.Geurts, MarjoleinSchulze, JohannesGevertz, JanaSchumacher, NeeleGhadjar, PirusSchumacher, UdoGhai, VikasSchuster, IonaGhanim, BahilSchwaab, JulianaGhantous, YasmineSchwahn, Denise J.Gharahkhani, PuyaSchwarz, ChristophGhavami, SaeidSchwind, SebastianGhayee, HansScialdone, AntonioGhelli Luserna Di Rorá, AndreaScilimati, AntonioGhersi, GiulioScimeca, ManuelGhidini, MicheleSconocchia, GiuseppeGhidoni, RiccardoScott, RodneyGhilli, MatteoScuoppo, ClaudioGhione, PaolaSeano, GiorgioGhiringhelli, FrançoisSebastian, CarlosGhiroldi, AndreaSedda, GiuliaGhislat, GhitaSedgwick, Alanna E.Ghobadi, GhazalehSedic, MirelaGhobadloo, Shahrokh MohammadzadehSee, ViolaineGhosh, SantoshSeeber, AndreasGhosh, SouravSeetharam, MaheshGhoshal, KalpanaSeewaldt, Victoria L.Giaccone, LuisaSegatto, MarcoGiacomelli, ChiaraSeger, RonyGiacomelli, LucaSegura, Miguel F.Giacomini, AriannaSehgal, LalitGiacomini, PatrizioSeidl, MaximilianGiaj Levra, MatteoSeiffert, MartinaGiakountis, AntonisSeipel, KatjaGiallongo, AgataSeitz, Anna KatharinaGiallongo, CesarinaSeki, NaohikoGiammanco, MarcoSekino, YoheiGianferrara, TeresaSelbo, Pål KristianGianfredi, VincenzaSeliger, BarbaraGiangregorio, FrancescoSeliger, CorinnaGiannakouros, ThomasSellner, LeopoldGiannella, LucaSelmin, OrnellaGiannini, ValentinaSeluanov, AndreiGiansanti, FrancescoSemczuk, AndrzejGibbs, PeterSempere, LorenzoGibert, YannSen, MalabikaGieseler, FrankSenbonmatsu, TakaakiGiger, RolandSeppälä, Toni T.Giglio, FabioSepúlveda Sánchez, Juan ManuelGiglio, PierreSeracchioli, RenatoGil, JeovanisSerafin, ValentinaGil, JustynaSerganova, InnaGilbert, Duncan C.Serin-Harmanci, AkdesGill, HarinderSerio, GabriellaGill, Martin R.Sério, SusanaGilles, ChristineSerrador Peiró, Juan M.Gillespie, James W.Serralheiro, PedroGillet, Jean-PierreSerrano, Maria JoseGillies, RobertSerre-Beinier, VéroniqueGimenez-Roqueplo, Anne-PauleSersa, GregorGiordano, AntonioSertorio, MathieuGiordano, CinziaSeruca, RaquelGiordano, FrancescaSeshadri, MukundGiordano, GuidoSessa, CristianaGiordano, SilviaSetaluri, VijayasaradhiGiorgi, Ugo DeSethi, GautamGiovannelli, PiaSeto, EdwardGiovannetti, ElisaSeto, TiffanyGiovannoni, RobertoSetty, BhuvanaGiovannucci, David R.Ševčík, JanGiovarelli, MatteoSevcikova, SabinaGiovarelli, MirellaSevenich, LisaGirard, Pierre-MarieSeveri, StefanoGisbert-Garzarán, MiguelSeverino, PatríciaGisselbrecht, ChristianSevilla, MichaelGiudetti, AnnaSfacteria, AlessandraGiuffrida, PaoloSforna, LuigiGiuliani, Anna LisaSgambato, AlessandroGiuliani, NicolaSgouros, JosephGiuliano, MichelaShabanzadeh, Daniel MønstedGiunchi, FrancescaShabason, JacobGiuriato, SylvieShackelford, JuliaGiurisato, EmanueleShah, JainilGiustacchini, AliceShah, NilayGiusti, IlariaShah, Rashmi R.Gjerdrum, Lise Mette RahbekShahbaaz, MohdGjertsen, Bjørn ToreShajahan-Haq, Ayesha N.Gjörloff Wingren, AnetteShakya, AkhileshGkika, EleniSham, Yuk YGkretsi, VasilikiShamay, YosefGlabska, DominikaShamseddine, AliGlass, JonShang, QingsenGlaß, MarkusShankar, EswarGlatz, TorbenShanmugam, MalaGleber-Netto, Frederico OmarShanti, Rabie M.Gleeson, NoreenShao, HaipengGlimelius, BengtShao, Yu-Hsuan JoniGlimelius, IngridShao, Yu-YunGloor, BeatShapiro, Charles L.Glover, Anthony R.Sharaf, KariemGlubb, DylanSharker, Shazid Md.Glubb, Dylan M.Sharma, AditiGmeiner, William H.Sharma, AnuragGnani, DanielaSharma, ArishyaGniazdowska, EwaSharma, GauravGnoni, AntonioSharma, GayatriGobert, Alain P.Sharma, GeetanjaliGobessi, StefaniaSharma, MeghaGodet, YannSharma, PrashantGodfraind, CatherineSharma, RitinGoding, ColinSharova, NataliaGodkin, AndrewSharp, TysonGodlewska, MarlenaShaul, Yoav D.Godlewski, JanuszShav-Tal, YaronGoel, ShreyaShaw, LisaGoffart, SteffiShayakhmetov, Dmitry M.Goffin, KarolienShcharbin, DzmitryGögenur, IsmailShe, Qing-BaiGogulamudi, Venkateswara ReddySheldrake, HelenGogvadze, VladimirShellman, YiqunGohla, AntjeShemesh, JosephGold, LarryShen, Cheng-HuangGoldberg, YaelShen, HaihongGolding, Jon P.Shen, Jia ZackGoldsberry, Whitney N.Shen, Tang-LongGoldsmith, Jason RShen, XiulongGoldstein, DavidSheng, KuanweiGoloubeva, OlgaSheng, YiGolovleva, IrinaSheng, YueGolubnitschaja, OlgaShepherd, TrevorGolubovskaya, VitaSher, Yuh-PyngGomes, Bruno CostaSherbet, Gajanan V.Gomes, CatarinaSherman, JonathanGomes-da-Silva, Lígia C.Sherriff, JennyGómez De Cedrón, MartaSheshadri, NamrathaGomez Gomez, EnriqueSheth, RahulGómez, Pilar SánchezSheu, Bor-ChingGomez-Gutierrez, JorgeShi, JianGonçalves, AnthonyShi, JunchaoGoncalves, Luís GafeiraShi, LeiGondi, Christopher SShi, WenyinGonfloni, StefaniaShiah, Shine-GwoGongora, CelineShibamoto, YutaGonsalves, Wilson I.Shibata, MahoGonzalez, AntonioShibata, YasushiGónzalez, EsperanzaShibuya, HiroshiGonzalez, Martin VillalbaShidal, ChrisGonzalez, MichelShidoji, YoshihiroGonzalez, Raul S.Shieh, Dar-BinGonzalez, RogelioShields, Mario A.González, SegundoShien, TadahikoGonzález-Bayón, LuisShiga, KiyotoGonzález-Granado, José MaríaShih, DavidGonzález-Minero, Francisco JoséShih, Jing-WenGonzález-Moles, Miguel ÁngelShih, Yin-HwaGonzalez-Sanchez, EsterShih, Yu-LuengGonzález-Sarmiento, RogelioShim, JaejunGonzález-Suárez, BegoñaShim, Joon W.Goos, Jeroen A.C.M.Shimada, ShingoGopal, KeshavShimamura, TakeshiGopalakrishnan, VidyaShimaoka, MotomuGordeeva, OlgaShimizu, KazuhideGordon-Weeks, AlexShimizu, KazuoGorgan, LucianShimizu, ToshiyukiGorini, FrancescaShimoda, MitsugiGorji, AliShimodaira, ShigetakaGorniak, PatrykShimojo, MasahitoGoroncy, AlexanderShimomura, HirofumiGorska-Ponikowska, MagdalenaShimono, YoheiGorvel, LaurentShimose, ShigeoGośliński, TomaszShin, Jae-HoGoto, AkiteruShin, Sang HyunGoto, HiroakiShin, Vivian YvonneGoto, TaichiroShinde, AparnaGoto, YusukeShingu, TakashiGotte, GiovanniShinji, TanakaGottesman, Michael M.Shinmura, KazuyaGotthardt, MartinShinohara, ShogoGottwald, EricShintani, YasushiGougelet, AngeliqueShiota, SeijiGouilleux, FabriceShioyama, YoshiyukiGowda, ChandrikaShipunova, Victoria OGoyal, RamanShiraishi, KenshirouGoyvaerts, CleoShiraz, ParveenGozzini, AntonellaShirokikh, Nikolay E.Grabiec, UrszulaShirole, NitinGracia, Jose Luis PerezShirotake, SuguruGracia-Sancho, JordiShivakumar, PranavGraciotti, MicheleShiver, LembitGraczyk-Jarzynka, AgnieszkaShnyder, SteveGradalski, TomaszShojaei, ShahlaGrade, MarianShoji, IkuoGradia, DanielaShoji, SunaoGräf, RalphSholl, Lynette M.Graf, Solomon A.Short, Nicholas JamesGraham, Nicholas A.Short, Susan C.Graillon, ThomasShrestha, RaunakGralow, JulieShridhar, VijiGramolelli, SilviaShu, SherryGranadeiro, Luiza BreitenfeldShukla, ArvindGranados-Principal, Sergio M.Shukla, GirishGrand, RogerShukla, KirtikarGrandgenett, PaulShukla, RuchiGrandi, VieriShukla, SiddharthGranito, AlessandroShukla, Surendra KumarGranja, Pedro L.Shukla, VivekGrannis, Frederic W.Sibilia, MariaGrant, IreneSibson, David RossGraslund, TorbjornSica, AntonelloGrassi, Elisa StellariaSiddharth, SumitGrassi, IlariaSiddique, Abu BakarGrassilli, EmanuellaSidhu, Stan B.Grąt, KarolinaSidles, Sara J.Gratchev, AlexeiSieber, OliverGray, Douglas A.Siegelin, MarkusGray, Janet M.Sieni, ElisabettaGraziani, GraziaSier, CornelisGrebien, FlorianSierko, EwaGreen, Alanna C.Sierżęga, MarekGreen, Mark A.Sifrer, RobertGreenberg, SamanthaSigala, SandraGreenberger, Joel S.Signore, AlbertoGreene, Mark I.Signore, GiovanniGreif, Philipp A.Signorelli, FrancescoGreinert, RüdigerSignori, EmanuelaGreinix, Hildegard T.Sihver, LembitGreither, ThomasSikic, DanijelGrenda, AnnaSikora, EwaGrendár, MarianSikora, MariuszGresele, PaoloSikorski, AleksanderGreten, TimSikov, William M.Grieb, PawelSill, HeinzGrieco, DomenicoSillerud, Laurel O.Griffin, Robert J.Silva, Ana LuísaGriffith, Thomas S.Silva, CatarinaGriffiths, L. R.Silva, Susana NunesGriffiths, RianSilva, WalterGriggs, Jennifer J.Silvennoinen, Olli JuhaniGrignani, GiovanniSilver, Daniel J.Grigoriadis, GeorgeSilvestri, GiovanninoGriguer, Corinne E.Silvestri, IdaGrillone, KatiaSilvestri, MarcoGrimaldi, Anna MariaSilvestris, NicolaGrimaldi, GiovannaSim, Sung HoonGrimm, ChristianSimão, André L.Grimm, ChristinaSimao, TatianaGrinband, JackSimiczyjew, AleksandraGrindedal, Eli MarieSimioni, CarolinaGrippo, Paul JSimmen, Frank A.Gritli-Linde, AmelSimon, ChristianGrobmyer, Stephen R.Simon, JorgeGrolla, AmbraSimona, KranjcGronnier, CarolineSimona, SivoriGronwald, WoframSimonato, AlchiedeGronwald, WolframSimone, CristianoGros, LaurentSimonetti, GiorgiaGros, Stephanie J.Simonetto, CristoforoGrose, DerekSimon-Keller, KatjaGross, Julia ChristinaSimons, Brian WesleyGross, Stephane R.Simpson, Benjamin S.Grosser, OliverSimpson, PeterGrossi, ValentinaSingh, AbhishekGrossman, Ashley BarrySingh, Ajay PratapGrotz, Travis E.Singh, AmritaGrötzinger, CarstenSingh, AnandGrover, NatalieSingh, AnilGrube, SusanneSingh, AnupGrumolato, LucaSingh, AnuragGrünewald, ThomasSingh, Anurag K.Grüngreiff, KurtSingh, BhagirathGruszczynska-Biegala, JoannaSingh, Brijesh KumarGruszka, Alicja MSingh, DeoGruters, Rob A.Singh, GagandeepGrützner, FrankSingh, ManishaGrywalska, EwelinaSingh, NaveenGu, JianSingh, NeenuGu, WenyiSingh, ParamGu, XinbinSingh, Rakesh K.Gu, YianSingh, RatnakarGu, YuchaoSingh, Santosh KumarGuadagni, FiorellaSingh, SarbjitGuan, FadaSingh, ShaileshGuan, JikuiSingh, Sharda P.Guan, QingdongSinghal, Sandeep K.Guan, YihongSinha, Birandra K.Gubbi, SriramSinha, DebottamGudbergsson, Johann MarSinha, NiharikaGudey, Shyam KumarSinha, PratikGuduru, ShivaSinha-Ray, SumanGuerini Rocco, ElenaSinkovic, AndrejaGuerrini, LucaSinnberg, Tobias W.Gugel, IsabelSiracusano, SalvatoreGugnoni, MilaSiravegna, GiuliaGuha, ChandanSirinian, ChaidoGuha, Prajna P.Šírová, MiladaGuida, AgostinoSirvent, AudreyGuidolin, DiegoSisti, AndreaGuièze, RomainSisu, CristinaGuilford, ParrySitarz, RobertGuilhot, FrançoisSitcheran, RaquelGuillaud, MartialSiva, ShankarGuillaume, Thierry A.Sivakumar, SushamaGuillén-Ponce, CarmenSivalingam, Vanitha N.Guillerey, CamilleSizochenko, NataliaGuillot, AdrienSjöberg, KlasGuinn, Barbara-AnnSjödahl, GottfridGuiu, BorisSkandalis, SpyrosGujar, Amit D.Skeie, Bente SandveiGulic, TamaraSkerjanc, AlenkaGulino, RosarioSkoda, JanGuller, AnnaSkogen, KarolineGulley, MargaretSkoko, John J.Gumulec, JaromirSkordalakes, EmmanuelGunaje, JayaramaSkorska, AnnaGunasekharan, Vignesh KumarSkrap, MiranGunda, VenugopalSkroza, NevenaGundamaraju, RohitSkrzypek, KlaudiaGundara, JustinSkuja, SandraGunn, TeresaSkvortsova, Ira-IdaGunnarsson, UlfSlade, NedaGunter, Jennifer H.Slawson, ChadGünzel, DorotheeSlim, NajlaGuo, BinSlominski, AndrzejGuo, Charles ChuanhaiSmaglo, Brandon G.Guo, DeliangSmahel, MichalGuo, KaiSmall, AlexGuo, SiqiSmedby, Karin EkströmGuo, TingSMEENK, Robert-JanGuo, WeiSmetana, KarelGuo, WeiminŚmieszek, AgnieszkaGuo, YanSminia, PeterGuo, Zong ShengSmit, LindaGupta, Gagan D.Smith, David F.Gupta, MohitSmith, Deanna S.Gupta, NirzariSmith, JeffGupta, Ramesh C.Smith, JulianneGupta, SanjaySmith, LoreyGupta, SupritSmith, Maree T.Gupta, VijayalaxmiSmith, Miriam J.Gurrapu, SreeharshaSmith, Steven S.Gust, Kilian MartinSmith, Stuart J.Gustafson, Michael P.Smits, EvelienGustav, StalhammarSmits, RonGütgemann, InesSmitt, Peter A.E. SillevisGüthlin, CorinaSmolkova, BozenaGutierrez-Camino, AngelaSmolková, KatarinaGutschner, TonySmolle, JosefGuttery, David S.Smolle, Maria AnnaGutzmer, RalfSnead, David R.J.Guyot, BorisSniadecki, MarcinGuyot, ErwanSnijders, Tom J.Guzhova, Irina V.Snowsill, TristanGwin, KatjaSo, JonathanGyorffy, BalazsSo, Samuel KSHa, Ki-TaeSoares, HelenaHa, Yun-SokSoares, PaulaHaarmann-Stemmann, ThomasSobacchi, CristinaHaas, OskarSobhani, NavidHaas, RickSobinoff, AlexanderHaass, Nikolas K.Sobotta, ŁukaszHabib, NagySocea, BogdanHacker, NevilleSocorro, SilviaHackert, ThiloSoda, PaoloHackl, HubertSoejima, ToshinoriHackner, KlausSoffietti, RiccardoHaderlein, MarlenSokilde, RolfHadid, TarikSokol, ElizabethHadziselimovic, FarukSolanki, SumeetHaendler, BernardSolano, AngelaHafeez, BilalSolé Cañadas, CarlaHafizi, SassanSolich, JoannaHafner, ChristineSolimando, Antonio GiovanniHäfner, NormanSolinas, AntonioHagemann, CarstenSolinas, CinziaHaglund, FelixSolingapuram Sai, Kiran KumarHahn, OliverSolli, PiergiorgioHahne, JensSollini, MartinaHahne, MichaelSoloff, Adam C.Hai, HoangSoltész, BeátaHaier, JörgSomasundarama, Dinesh BabuHaines, JefferySomlyai, GáborHaines, Julie ReiszSommariva, MicheleHaining, Robert L.Somoza, ÁlvaroHait, Nitai C.Somoza, RodrigoHajeri, PraveensinghSon, Deok-sooHalaban, RuthSon, JaekyoungHalabi, CarmenSonbol, Mohamad B.Halachmi, SarelSønderkær, MadsHałas-Wiśniewska, MartaSone, HidekoHalberg, NilsSonego, MauraHalbrook, ChristopherSonenberg, NahumHaley, John D.Song, Chang MyeonHalkia, Evgenia A.Song, Chang W.Hall, Carolyn SSong, JaewhanHall, MarciaSong, JianxunHallberg, BengtSong, MinjungHaller, FlorianSong, XinhuaHalmos, GáborSong, YanHam, Won SikSonnen, AndreasHamada, MasakazuSonowal, HimangshuHamaguchi, MasahideSoond, Surinder M.Hamamura, KazunoriSorace, Anna G.Hamanishi, JunzoSorahan, TomHamburger, Anne W.Sorbye, Sveinung WergelandHamel, LaurenSørensen, Anne VestHamidi, OksanaSorensen, Poul H.Hamieh, MohamadSorokin, Maxim I.Hammer, AnneSorolla, Maria AlbaHammond, EsterSorrenti, ValeriaHan, AiguoSossey-Alaoui, KhalidHan, ChialiSottnik, JosephHan, DohyunSouazé, FrederiqueHan, DongSoubeyran, PhilippeHan, IhnSouček, PavelHan, Jae YongSouglakos, JohnHan, MingzhiSoulières, DenisHan, SongSoumelis, VassiliHan, Sun-YoungSounni, Nor EddineHan, YanhuiSourbier, CaroleHan, ZhiSousa, AngelaHanash, Samir M.Sousa, Sérgio FilipeHancock, Wayne W.Sousa, SofiaHanda, HiroshiSoutham, AndrewHandzlik, JadwigaSouweidane, Mark M.Hanemann, OliverSozzi, DavideHanke, TomasSpaargaren, MarcelHanley, ChirstopherSpagnol, GaëlleHann, Hie-WonSpagnolo, FrancescoHannafon, Bethany NoelleSpagnuolo, CarmelaHannah-Shmouni, FadySpan, PaulHansen, Adam E.Spanu, AngelaHansen, Jakob WernerSpartalis, EleftheriosHansen, Marc D. H.Spatz, AlanHansen, Torben FrøstrupSpears, MelanieHans-Robert, MetelmannSpennato, PietroHantschel, OliverSperduti, IsabellaHao, MingangSpernyak, JosephHao, YuhanSperti, CosimoHaque, ReinaSpiegelman, Vladimir S.Haque, ShabirulSpinella, FrancescaHara, ToshifumiSpinella, MichaelHarada, Bryan T.Spinelli, LauraHarada, HisashiSpinelli, OriettaHarada, MasakoSpirina, Liudmila V.Harashima, NanaeSpitzer, DirkHaratani, KojiSpoletini, GabrieleHardcastle, IanSportoletti, PaoloHardell, LennartSprave, TanjaHardiman, GarySpring, KevinHardingham, Jennifer E.Sridurga, MithraprabhuHardisson, DavidSrimathveeravalli, GovindHardt, JuliaSrivastava, AkhilHareendran, SangeethaSrivastava, RiteshHargreaves, IainSrivastava, Swayam PrakashHarkness, Elaine F.Sroka, RonaldHärkönen, PirkkoStaaf, JohanHarky, AmerStaber, Philipp BernhardHarrell, J. ChuckStachowicz-Stencel, TeresaHarrington, BrittneyStack, SharonHarris, MitchelStacker, StevenHarrison, Mark K.Stadlbauer, AndreasHarrop, RichardStadler, KrisztianHart, GeoffreyStaege, Martin S.Hart, Peter C.Stafford, Francis WilliamHartman, MariuszStagni, VenturinaHartsough, EdwardStalpers, LukasHasan, ProttoyStam, Ronald W.Hasan, TayyabaStancampiano, AugustoHasanzadeh Kafshgari, MortezaStanganelli, IgnazioHasegawa, HirotakaStankevičius, VaidotasHasegawa, TakaakiStanta, GiorgioHasegawa, TakumiStarek, MałgorzataHashemian, MaryamStark, Amy L.Hashida, NoriyasuStark, AzadehHaslene-Hox, HanneStark, Felicity C.Hassan, MohamedStark, JeremyHasse, SybilleStarr, Timothy K.Hassel, Jessica CecileStarrett, Gabriel J.Hata, YutakaStasiak, MagdalenaHatachi, YukimasaStathopoulos, GeorgiosHatakeyama, ShingoStauffer, PaulHatakeyama, ShinjiStavraka, CharaHatano, KojiStawski, RobertHatano, YuichiroStearns, Lisa J.Hatley, Mark E.Stec, RafałHato, TaiSteenbakkers, Roel J.H.M.Hattinger, Claudia M.Steenbergen, Renske D.M.Hattori, NaokoStefan, Simona CatalinaHatzl, StefanStefanello, SílvioHau, PeterStefano, AlessandroHaug, AlexanderStefanowicz, JoannaHauser, PeterSteigen, Sonja ErikssonHausser, AngelikaSteiger, KatjaHawkins, MariaSteinbach, JoachimHawkins-Daarud, AndreaSteinbichler, Teresa BernadetteHawse, John R.Stella, Giulia M.Hay, Michael P.Stella, StefaniaHayakawa, YokuStelmes, Jean-JacquesHayasaka, HarukoStemmler, MarcHayashi, KatsuhiroStene, Guro BirgitteHayashi, MakotoStensheim, HanneHayashi, RyujiStenvang, JanHayashi, TomokoStenz, LudwigHayashida, KenjiStenzl, ArnulfHaynes, HarryStepanenko, Aleksei A.Haynes, JenniferStephens, Andrew N.Hazane-Puch, FlorenceStephenson, Sally-AnneHazawa, MasaharuStepp, HerbertHazenberg, Mette D.Sterba, JaroslavHazlehurst, Lori AStergios, KonstantinosHe, ChunboStern, PeterHe, FengSterpetti, Antonio V.He, HongSteven, AndreHe, HongliangStevens, AlexandraHe, JingStevenson-Lerner, HeatherHe, YazhouStewart, JasonHe, YiStewart, Lamonica V.He, YukaiStewart, MurrayHeakal, YasserStewart-Ornstein, JacobHebbard, LionelStintzing, SebastianHecht, MarkusStocco, GabrieleHecker, AndreasStock, ChristianHedglin, MarkStock, PeggyHedwig, Hedwig Sutterlüty-FallStockmann, ChristianHee-chul, ParkStoecklein, NikolasHeeke, SimonStoian, DanaHeery, David M.Stoklosa, TomaszHegedüs, BalazsStope, MatthiasHehlgans, StephanieStorti, PaolaHehlmann, RüdigerStossi, FabioHeiblig, MaëlStoyanova, TanyaHeidel, FlorianStragliotto, GiuseppeHeiland, Dieter HenrikStrano, SabrinaHeindryckx, FemkeStraten, Per ThorHeinrich, StefanStrati, AretiHeitzer, EllenStrati, KaterinaHeldin, ParaskeviStrati, PaoloHelfrich, IrisStraub, BeateHelguero, Luisa A.Straume, OddbjØrnHeller, GerwinStrauss, LauraHeller, RichardStravopodis, Dimitrios J.Hellerbrand, ClausStrbenac, DarioHellevik, TuridStreby, Keri A.Hellman, PerStripay, JenniferHelminen, OlliStrippoli, RaffaeleHendrix, Mary J.C.Strnadel, JanHenen, Morkos A.Ströbel, PhilippHeng, BenjaminStroffekova, KatarinaHenklewska, MartaStrojan, PrimožHennrich, UteStröm, LenaHenrique, RuiStrömblad, StaffanHenry, ClaireStrowitzki, MoritzHensiek, Anke E.Strus, MagdalenaHenson, JeremyStrzelecki, MichalHenson, JohnStubenrauch, FrankHentrich, Marcus U.Stuelten, Christina H.Heo, Tae-HweStühmer, ThorstenHerichova, IvetaStur, ElaineHermanns, ThomasSturiale, Carmelo LucioHermeking, HeikoSturtzel, CaterinaHermouet, SylvieStuschke, MartinHernandez Rivas, Jesus MariaStylianou, AndreasHernández, ÁngelStylli, StanleyHernández-Aguado, IldefonsoSu, Bor-ChyuanHernandez-Ilizaliturri, FranciscoSu, Chien-WeiHernández-Núñez, EmanuelSu, Gloria H.Hernández-Torres, FranciscoSu, Po-HsuanHerndler-Brandstetter, DietmarSu, Ruey-ChyiHerold, NikolasSu, SiyuanHerraez, ElisaSu, Wu-ChouHerrero, Edgar PerezSu, XinmingHerrmann, AnneSuami, HirooHersey, PeterSuárez-Cabrera, CristianHershberger, PamelaSuarez-Carmona, MeggyHershkovitz, DovSuarez-Cunqueiro, María MercedesHertz, Daniel L.Suárez-Ibarrola, RodrigoHervouet, EricSubbannayya, YashwanthHéry-Arnaud, GenevièveSubhi, YousifHes, OndrejSubramaniyan, BoopathiHeske, ChristineSuchorska, WiktoriaHess, AngelaSuciu, Bogdan AndreiHessheimer, Amelia J.Sud’ina, Galina F.Hester, JoannaSuda, GokiHetta, Helal F.Suda, KenichiHeumann, RolfSudhan, DhivyaHeurtault, BéatriceSuenaga, MitsukuniHeymann, DominiqueSugie, TomoharuHeymann, FelixSugimoto, MitsushigeHibi, TaizoSugimura, HaruhikoHickey, Michael J.Suh, Sung-SukHicks, James BSuhail, YasirHickson, IanSujobert, PierreHieda, MikiSuk, Jung SooHier, M.P.Sukawa, YasutakaHiggins, GeoffreySukhorukov, VladimirHiggins, Paul J.Sukocheva, OlgaHiguchi, RyotaSukowati, Caecilia H.C.Higurashi, TakumaSukul, PritamHildebrandt, Malene GrubbeSukumar, PiruthiviHildenbrand, GeorgSukumar, SaraswatiHildreth, Blake E.Sulová, ZdenkaHildreth, EasonSümser, KemalHill, MarkSun, ChenHill, MichelleSun, DuanpingHill, RebeccaSun, GongqinHillmer, Axel M.Sun, JieHiltermann, T. Jeroen N.Sun, JunHindié, ElifSun, KunfengHinds, Philip W.Sun, RamonHines, StellaSun, WenyueHingorani, DinaSun, WinnieHippo, YoshitakaSun, Xiao-FengHiraga, ToruSun, YapingHirano, HidekazuSun, YeHiraoka, NobuyoshiSun, YinHirsch, EmilioSun, ZhixiongHisatsune, TatsuhiroSun, ZhonghuaHiscott, JohnSunakawa, YuHishikawa, YoshioSundaramoorthy, PasupathiHitt, RicardoSundararajan, RajiHo, Cheng-HsunSundín, Anders E.Ho, Cheng-MawSundvall, MariaHo, ChengyingSung, Wen-WeiHo, Jar-YiSupiot, StéphaneHo, VincentSur, SubhayanHo, Xuan-HungSureban, Sripathi M.Hoang, Mai P.Sureda, AnnaHoang, Ngoc HaSurguchov, AndreiHoashi, ToshihikoSurmacki, Jakub MaciejHobbs, Gabriela SorianoSurmacz, EvaHodge, James W.Surov, AlexeyHoehn, KyleSurowka, Artur DHoerner, ChristianSuso, Else Marit InderbergHofauer, BenediktSutton, MargieHofer, SilviaSuwan, KeittisakHoffman, Robert M.Suzui, MasumiHoffmann, Jean-SebastienSuzuki, HideoHoffmann, KatrinSuzuki, HiroetsuHoffmann, MarkusSuzuki, MaikoHofman, JakubSuzuki, MasatakaHofman, PaulSuzuki, ShinichiHofmann, PeterSuzuki, ShugoHofr, CtiradSuzuki, SusumuHohaus, StefanSuzuki, TakafumiHohenberger, PeterSuzuki, ToyofumiHohenegger, MartinSuzuki-Karasaki, YoshihiroHohmann, Urszula GrabiecŠvastová, EliškaHojjat-Farsangi, MohammadSvendsen, Lars BoHojski, AljazSverdlov, AaronHokuto, DaisukeSvirshchevskaya, Elena V.Holley, CristopherSwami, UmangHollis, Robert L.Swanson, Kenneth D.Hollomon, Mario G.Swartling, FredrikHolmstrøm, KimŚwiątek, PiotrHolmström, MortenSwiatkowska, AgataHolodņuka, IrinaSykiotis, Gerasimos P.Holubekova, VeronikaSymer, DavidHölzel, MichaelSyndikus, IsabelHolzmann, KlausSzabo, CsabaHombach, Andreas A.Szanto, MagdolnaHomma, Miwako KatoSzarvas, TiborHomma, TetsuyaSzaryńska, MagdalenaHompland, TordSzász, Attila MarcellHonecker, FriedemannSzatmári, TündeHoneychurch, JamieSzczepanek, JoannaHong, Andrew L.Szebeni, Gábor JánosHong, ChangwanSzeliga, MonikaHong, In-SunSzemes, MariannaHong, Su-HyungSzendrői, AttilaHong, XinSzewczuk, Myron R.Hong, XupengSzkandera, JoannaHong, Yi-RenSznarkowska, AlicjaHonrao, ChandrashekharSzolnoky, GyozoHooda, JagmohanSzoor, ArpadHoogwater, FrederikSztuba-Solinska, JoannaHooper, Douglas C.Szubert, SebastianHooper, John D.Szyc, ŁukaszHöpfner, MichaelSzydlowski, MaciejHopp, KatharinaTaberlay, PhillippaHoras, KonstantinTabernero, ArantxaHorbinski, CraigTacconelli, StefaniaHorejsí, VáclavTachimori, YujiHorger, MariusTada, RuiHori, ToshiyukiTafuto, SalvatoreHorikawa, IzumiTagad, HarichandraHorin, PetrTagliabue, EldaHörmann, GregorTagliafico, EnricoHormuth, DavidTagliamonte, MariaHörner-Rieber, JulianeTaguchi, AyumuHorstmann, MarcusTaguchi, YoshihiroHosaka, KayokoTai, DavidHoshiba, TakashiTaieb, JulienHoskins, ClareTaipaleenmäki, HannaHosmalin, AnneTakabatake, KiyofumiHosmane, NarayanTakada, IchiroHosnijeh, F. SaberiTakagi, KiyoshiHossain, FokhrulTakagi, MotokiHosui, AtsushiTakahari, DaisukeHottinger, Andreas F.Takahashi, ChiakiHou, JueTakahashi, JunkoHou, RuixueTakahashi, YutakaHouben, RolandTakai, AtsushiHoudijk, A. P. J.Takamaru, HiroyukiHour, Tzyh-ChyuanTakamatsu, ShigeyukiHourigan, ChristopherTakano, KenichiHouse, CarrieTakata, KeiichiHouse, ImranTakayama, KanakoHoushmand, MohammadTakeda, HaruhikoHoving, EelcoTakegahara, NorikoHow, JoanTakenaka, YukinoriHowell, ViiveTaketo, Makoto MarkHoy, AndrewTakigawa, NagioHrckulak, DusanTalati, ChetasiHrvat, AntonioTalbot, ChristopherHsaio, MichaelTalesa, Vincenzo NicolaHsiao, Hui-HuaTallet, Agnès V.Hsiao, Kuei-YangTalmadge, JamesHsiao, Sheng-MouTalora, ClaudioHsiao, Yi-HsuanTalukdar, Fazlur RahmanHsieh, Jason Chia-HsunTalukdar, SarmisthaHsieh, Ming-ChingTam, Samantha H.Hsieh, Pei-LingTam, WayneHsieh, Tsung-HanTama, Bayu AdhiHsieh, Yih-ShouTamagnone, LucaHsieh, Yi-HsienTamaian, RaduHsu, Chai-HsienTamaki, NobuharuHsu, Chia-LangTamary, HannahHsu, FeitingTamkovich, SvetlanaHsu, Fei-TingTamminga, MennoHsu, Jun-TeTamrazi, BenitaHsu, Li-HanTamura, HidetoHsu, Ping ChihTan Boon, TohHsu, Ren-JunTan, Aaron C.Hsu, Shih-HsienTan, Aik ChoonHsu, Sung-PoTan, Bertrand Chin-MingHsu, Tsung-I.Tan, David SpHsu, Yi-ChiungTan, KarenHsuan, JustinTan, Shyh HanHu, BaoliTan, Tuan ZeaHu, DingyuanTan, WinstonHu, Dong GuiTan, YutingHu, GuangzhenTanabe, HirokiHu, GuokuTanabe, KenjiHu, JiafenTanabe, ShihoriHu, KebinTanaka, HiroakiHu, Peter C.Tanaka, HiromiHu, ShiminTanaka, JunjiHu, ShuiyingTanaka, KozoHu, TaoboTanaka, NobuyukiHu, YingTanaka, TakemiHu, YinlingTanaka, TakujiHu, YuTanaka, ToshikoHuang, Alexander C.Tanaka, YasuoHuang, Bing-ShenTanaka, YoshimasaHuang, GangTanda, Enrica TeresaHuang, Guan-JhongTang, Amy H.Huang, Hsien-BinTang, DamuHuang, Hsuan-YingTang, Kwan HoHuang, HuachaoTang, Patrick Ming-KuenHuang, Huang ChiaoTang, TaoHuang, Jason H.Taniguchi, Cullen M.Huang, Kai WenTaniguchi, HiroakiHuang, KaiwenTanis, PieterHuang, MarilynTaniwaki, MasafumiHuang, Ruby Yun-JuTaniyama, YusukeHuang, Ruea-YeaTankiewicz-Kwedlo, AnnaHuang, Shiang-FuTanti, Goutam KumarHuang, Shu-PinTantos, ÁgnesHuang, Tse-HungTao, JunyanHuang, Tze-SingTapio, SoileHuang, Weei-YuarnTarallo, RobertaHuang, Wei-ChienTarallo, ValeriaHuang, WeihuaTarantelli, ChiaraHuang, Wen-ChinTaratula, OlenaHuang, XiaohuTarazona Lafarga, RaquelHuang, XuTarkowski, RafalHuang, XudongTarn, Woan-YuhHuang, Yi-HsiangTarnowski, MaciejHuang, YingTartour, EricHuber, PeterTashima, ToshihikoHuber, StephanTasso, RobertaHübner, MartinTate, ShinichiHübner, MaxTateishi, KensukeHuczyński, AdamTátrai, PéterHuda, NazmulTattikota, Sudhir GopalHudlikar, RasikaTaubert, HelgeHughes, KevinTauriello, DanieleHugo, WillyTavares, AnthonyHühns, MajaTavassoly, ImanHuhtinen, KaisaTawara, KenHui, XuanTaylan, FulyaHulin-Curtis, SarahTaylor, AlisonHumbert, MagaliTaylor, Justin W.Hummel, Trent R.Taylor, Paul C.Humphries, BrockTazawa, HiroshiHung, Chao-HungTazzari, MarcellaHung, Chin-ChuanTazzioli, GiovanniHung, Jan-jongTchoghandjian-Auphan, AurélieHung, Jung-TungTe Riele, HeinHung, Ming-SzuTeague, Ryan M.Hung, Yao-ChingTedaldi, GianlucaHuntington, Nicholas DavidTeerds, Katja J.Huo, Teh-IaTeh, Muy-TeckHupe, Marie C.Teishima, JunHuppi, KonradTeixeira, João M. C.Hur, HoonTeixidó, CristinaHur, Man-wookTekpli, XavierHurley, DanielTella, Sri HarshaHurwitz, LaurenTellier, MichaelHurwitz, StephanieTelukutla, Srinivasa ReddyHusak, ZvenyslavaTemblador, ArturoHussain, Ahmad FawziTemraz, SallyHussaini, HaizalTen Dijke, PeterHussein, SarfarazTeng, JianHutchison, Robert E.Teng, NelsonHutson, AlanTeng, ShaoleiHutterer, Georg C.Teng, YongHutvagner, GyorgyTentes, Antonios-Apostolos K.Huvenne, WouterTeply, Benjamin A.Huynh, HungTepper, Clifford G.Hwa Soung, YoungTerai, MizueHwang, Tae-HoTerao, MinekoHwang-Verslues, Wendy W.Terbuch, AngelikaHyde, Ricia KatherineTerenzi, AlessioHyun, Kyung-A.Terenziani, MonicaHyun-jung, ParkTeresa Pereira, RaposoIaccarino, IngramTerhaard, Chris H.J.Iacobucci, IlariaTerlizzi, MichelaIafolla, MarcoTerp, Mikkel GreenIannaccone, AndreaTerracciano, DanielaIannolo, GioacchinTerris, Martha K.Iavarone, FedericaTerry, SamanthaIbusuki, MutsukoTerry, Stephen J.Ichim, GabrielTerzolo, MassimoIchim, ThomasTesauro, CinziaIdbaih, AhmedTesson, MathiasIde, HisamitsuTessoulin, BenoitIdel, ChristianTesta, UgoIdoate, MiguelTestori, Alessandro A.E.Idogawa, MasashiTêtu, PaulineIdowu, TemiloluTeugels, ErikIeni, AntonioTeusch, NicoleIentile, RiccardoTha, Khin KhinIerardi, EnzoThaker, Youg RajIesari, SamueleThakor, Avnesh SIgaz, PeterThakor, NehalIgbaria, AeidThakur, ArchanaIhara, YoshitoThakur, ChitraIjare, Omkar B.Thangaraju, MuthusamyIjichi, HideakiThangavel, ChellappagounderIkematsu, HiroakiThanikachalam, KannanIkezawa, KenjiThekkinkattil, Dinesh K.Ikura, YoshihiroThellung, StefanoIl’yasova, DoraThemistocleous, MariosIlardi, GennaroTheocharis, AchilleasIllei, Peter B.Theodoraki, Marie-NicoleIlluminati, GiulioTheodoropoulos, GeorgeIm, Chang-NimTherese, RiedemannIm, Hyung-JunTheret, NathalieImai, ChihayaTheurich, SebastianImai, ToshioThevenod, FrankImai, YasuoThews, OliverImai, YoichiThiel, KristinaImamura, MasahiroThiele, CarolImamura, ToshihikoThieme, RenéImbert-Fernandez, YoannisThierfelder, KoljaImbriano, CarolThijssen, RachelImparato, GiorgiaThillays, FrançoisIn’t Zandt, ReneThiruvengadam, MuthuInchingolo, FrancescoThomas, AlexandraIncoronato, MariarosariaThomas, Carole D.Incorvaia, LorenaThomas, ClémentIndini, AliceThomas, GarethIndiveri, CesareThomas, ThresiaInfante, PaolaThompson, Henry J.Infante, RodneyThompson, JoyceInfantino, VittoriaThompson, Reid F.Inga, AlbertoThompson, RuthInglada-Pérez, LucíaThomssen, ChristophIngrosso, GianlucaThrasivoulou, ChristopherIngvarsen, Signe ZiirThrower, EdwinInkielewicz-Stepniak, IwonaTiao, Greg M.Inman, GarethTiberio, Guido Alberto MassimoInngjerdingen, MaritTibiletti, Maria GraziaInnominato, PasqualeTie, JeanneInomata, MinehikoTien, An-ChiInoue, KazushiTiffen, Jessamy C.Inoue, SatoshiTigas, SteliosInoue, TakahiroTigyi, GaborInscoe, ChristinaTikhanovich, IrinaInzani, FredianoTikhomirova, IrinaIommelli, FrancescaTímár, JózsefIonescu, Daniela C.Timchenko, NikolaiIrace, CarloTiminszky, GyulaIrato, PaolaTimms, Kirsten M.Iravani, AmirTimoshenko, Alexander V.Iriuchishima, HironoTing, DavidIrshad, SheebaTing, Wen-ChienIrum, KhanTinhofer-Keilholz, IngeborgIrwin, David C.Tinkle, ChristopherIsenberg-Grzeda, ElieTiribelli, ClaudioIshibashi, MasumiTiribelli, MarioIshibashi, OsamuTiriveedhi, VenkataswarupIshida, FumihiroTischler, ArthurIshido, KeinosukeTiso, NatasciaIshii, TetsuroTissier, Paul LeIshikawa, EiichiTittarelli, AndrésIshikawa, HitoshiTitus, MarkIshikawa, ShigeoTixeira, RochelleIshikawa, TakeshiTiziani, StefanoIshikawa, ToruTjan-Heijnen, VivianneIshiyama, HiromichiTobin, Desmond J.Ishizuka, MitsuruTodaro, MatildeIshmael, Jane E.Toden, ShusukeIsidori, AlessandroTodsen, TobiasIsidoro, CiroToffoli, GiuseppeIslam, SharifulTognolatti, PieroIsokpehi, RaphaelTogo, ShinsakuIsola, GaetanoToh, UhiIsola, MiriamToietta, GabrieleItaliano, AntoineToillon, Robert-AlainItami, JunTokas, TheodorosItatani, YoshiroTokumaru, YoshihisaItkonen, HarriTokunaga, FuminoriIto, HidemiTokushige, KatsutoshiIto, HiromichiToland, AmandaIto, Ken-ichiToledano, Michel B.Ito, ShigekiToledo, FranckIto, TakamichiTolmachev, VladimirIto, YasuhiroTolosa, EzequielItoh, SusumuTomao, FedericaItoh, YukihiroTomasetti, MarcoItou, JunjiTomasetto, CatherineIurescia, SandraTomasetto, Catherine-LaureIvanusic, Jason J.Tomasik, BartłomiejIwadate, YasuoTomassetti, AntonellaIwamoto, ShotaroTomita, YusukeIwanicki, MarcinTomiyama, ArataIwano, TomohikoTomiyama, NoriyukiIwasa, TsutomuTomoaki, OkimotoIwata, HiromitsuTomuleasa, CiprianIwata, ShintaroTong, JessicaIwata, TakashiTong, JingshanIyer, JanakiTong, ManIyer, S.P.Tong, Yat-ChingIzasa, HisashiTonini, GiuseppeIzawa, TakeshiToomey, SineadIzbicki, JakobTornillo, GiusyIzonin, IvanTörnquist, KidIzumi, HirokiTorralba-Cabeza, Miguel-ÁngelIzumi, KoujiTorras, JuanIzzo, PaolaTorrens-Mas, MargalidaIzzotti, AlbertoTorrente, LauraJaakkola, Panu M.Torres-Ayuso, PedroJabłonowski, ZbigniewTorres-Cabala, Carlos AntonioJablonska, EwaTorres-Sangiao, EvaJablonska, KarolinaTorrisani, JérômeJacenik, DamianTorsello, BarbaraJacevic, VesnaTortosa, AvelinaJacinto, EstelaToruner, Gokce AltayJacky G., GoetzTorzilli, GuidoJacob, FrancisTosato, GiovannaJacobsen, AndersToscani, DeniseJacomin, Anne-ClaireToschi, ElenaJacot, WilliamToshimitsu, YamaokaJacquelot, NicolasTosi, DiegoJacquot, YvesToth, RekaJadiya, PoojaToth, ZsoltJager, AgnesTotsingan, FilbertJäger, TarkanTotta, PierangelaJaggi, MeenaTotzeck, MatthiasJagiello-Gruszfeld, AgnieszkaTouahri, YacineJahangiri, LeilaTournigand, ChristopheJahns, HanneToussaint, LauraJain, PrashiTouw, Ivo P.Jain, PreeteshTovoli, FrancescoJain, VaibhavToyama-Sorimachi, NorikoJakobi, TobiasToyohara, JunJakopovic, MarkoTrafalis, Dimitrios T.Jakowatz, JamesTran, Anh NhatJakubíková, JanaTran, BenJakubowska, Monika A.Traub, FrankJalving, MathildeTraverso, AlbertoJamieson, Stephen M.F.Treglia, GiorgioJan, Ming-ShiouTremblay, DouglasJanakiraman, HarinarayananTremblay, MichelJanczar, SzymonTrembley, JaneenJandova, JanaTrencsényi, GyörgyJanecki, MarcinTrepanier, AngelaJang, HyoncholTreps, LucasJang, Sung-WukTrevisson, EvaJangampalli Adi, PradeepkiranTribius, SilkeJanic, AnaTrikalinos, NikolaosJanik, StefanTrilling, MirkoJanis, Jeffrey E.Trimboli, PierpaoloJanjetovic, ZoricaTripathi, Lokesh P.Jankovic, MomciloTripathi, MadhulikaJankowska, AnnaTripathi, ManishJanody, FlorenceTripathi, ManishaJanovska, PavlinaTriulzi, TizianaJansen, C.A. (Christine)Trivedi, RachanaJansen, GerritTroch, MarleneJansen, SeppTroiano, GiuseppeJanssen, EmielTrombetta, DomenicoJanssen, StefanTrommer, MaikeJansson, Patric J.Troncone, GiancarloJanuchowski, RadosławTrosko, JamesJao, Tzu-MingTrotta, RossanaJara-Palomares, LuisTruong, Danh D.Jardé, ThierryTsai, Chieh-ChihJardin, IsaacTsai, Chi-NeuJares, PedroTsai, Han-MouJariel-Encontre, IsabelleTsai, Jen-NingJarstfer, Michael BruceTsai, Jen-PiJarzab, BarbaraTsai, Kun-LinJaskula-Sztul, RenataTsai, Ming-HanJászai, JózsefTsai, Ming-ShaoJauberteau, Marie-OdileTsai, Ting FenJaurand, Marie-ClaudeTsai, Wen SyJavaheri, TaherehTsai, Wen-ChiuanJaworek- Korjakowska, JoannaTsai, Yuan-ChenJaworek, JolantaTsakalof, AndreasJayaprakash, PriyamvadaTsang, Allen W.Jazaeri, AmirTsang, Kwong YokJazayeri, Seyed BehzadTsang, Suk-YingJazirehi, Ali R.Tsatsakis, AristidisJean Philippe, MetgèsTsaur, IgorJean, DidierTse, BrianJean, WalterTse, William K.F.Jeanne, TieTse-Dinh, Yuk-ChingJean-Quartier, ClaireTseng, Fan-GangJeck, William R.Tseng, Jen-ChihJędrzejewski, TomaszTseng, Ruo-ChiaJeffree, Rosalind LindyTsiaoussis, JohnJenkins, GarethTsilimigras, Diamantis I.Jenkins, RussellTsirigotis, PanagiotisJenkins, Samir V.Tsochatzidis, LazarosJennings, Jack W.Tsoi, HoJensen, Lars HenrikTsolakis, Apostolos V.Jensen, Lasse DahlTsoli, MariaJensen-Jarolim, ErikaTsoukalas, DimitrisJeon, Yoon KyungTsoukalas, NikolaosJeong, JoonTsuchida, KunihiroJeong, SeriTsuchimochi, MakotoJeong, Seung MinTsuchiya, HiroyukiJeremić, BranislavTsuchiya, KaoruJeremy, Teoh Yuen ChunTsuchiya, TomoshiJernberg-Wiklund, HelenaTsuchiya, YuichiJeroen T, BuijsTsuda, HiroyukiJerónimo, CarmenTsuda, HitoshiJerzak, Malgorzata MariaTsugawa, HitoshiJeschke, UdoTsui, Ke-hungJetten, AntonTsui, Kuan HaoJewett, AnahidTsuji, Atsushi B.Jhaveri, Kenar D.TSUJI, TakahiroJi, PengTsujii, MasayaJia, DongyaTsujimoto, HironoriJiang, ChunjieTsujinaka, ShingoJiang, HongTsukushi, SatoshiJiang, HuaileiTsunedomi, RyouichiJiang, JianxiongTsunematsu, TakaakiJiang, QingfeiTsuneoka, MakotoJiang, WeiTsushima, TakahiroJiang, XiaodongTsuzuki, ToyonoriJiang, XuntianTu, Shih-HsinJiang, YanyanTu, Shi-MingJiantai Timothy, QiuTu, ThomasJiao, Long R.Tucci, PaolaJimenez, CamiloTuech, Jean-JacquesJiménez-Bonilla, Julio FranciscoTulasne, DavidJiménez-González, GemaTulotta, ClaudiaJin, Dong-HoonTuma, Pamela L.Jin, JiefuTuosto, LorettaJin, XinTurashvili, GulisaJin, ZhaohuiTurcan, ŞevinJing, RanTurchi, John J.Jo, Dong HyunTurdo, AliceJo, Jung HyunTuretta, MatteoJo, Young SukTurhan, AliJoão, CristinaTurkowski, KatiJochmanova, IvanaTurksen, KursadJochum, ChristophTurley, Eva A.Joffe, ErelTurner, JonathanJohansson, PegahTurner, SunnaneJohns, Terrance G.Turrini, EleonoraJohnsen, StevenTuyaerts, SandraJohnson, Daniel E.Tuynman, Jurriaan B.Johnson, David G.Tvedt, Tor HenrikJohnson, IanTwarock, SörenJohnson, MiriamTwu, Yuh-ChingJohnson, Nathalie A.Tyagi, PradeepJohnson, NeilTyan, Yu-ChangJohnson, PaulineTycko, BenjaminJohnson, Peter FTyszka-Czochara, MalgorzataJohnson, RachelleTzanakakis, George N.Johnson, RoryTzang, Bor-ShowJolly, Mohit KumarTzankov, AlexandarJonckheere, NicolasTzeng, Shu-LingJones, BleddynUccella, SilviaJones, KyleUchida, SawakoJones, PeterUeno, TakayukiJones, R.L.Uetake, HiroyukiJones, VeronicaUgel, StefanoJones, WillUgga, LorenzoJongmans, MarjolijnUgolini, ClaraJonnalagadda, ShirishaUgun-Klusek, AslihanJono, HirofumiUhl, EberhardJørgensen, Anders PalmstrømUhlig, AnnemarieJoshi, Bharat H.Uhrberg, MarkusJoshi, KamalUjhǎzy, PeterJoshi, ShwetaUlasov, IlyaJoshua, Anthony M.Uldrijan, StjepanJosse, ClaireUlivi, PaolaJost, EdgarUlmert, DavidJoubert, NicolasUlrich, AlexisJour, GeorgeUlz, PeterJover, RodrigoUm, Hong-DuckJóźwicki, WojciechUmansky, ViktorJozwik, MaciejUmapathy, GaneshJue, Joshua S.Umemori, JuzohJuhasz, KataUmemura, AkiraJuhlin, ChristoferUmemura, AtsushiJuif, Pierre EricUmemura, NaokiJulia, AspUngefroren, HendrikJulià-Sapé, MargaridaUngerstedt, Johanna S.Jun, Sun-YoungUniacke, JamesJung, Alain C.UNNO, MichiakiJung, Chan KwonUpadhyay, GeetaJung, Ji HoonUpadhyaya, JasbirJung, KeehoonUprety, DipeshJung, KlausUra, BlendiJung, MichaelaUrabe, FumihikoJung, Yuh-SeogUrabe, YujiJunier, Marie-pierreUrashima, MitsuyoshiJunker, NielsUrbanelli, LorenaJunker, SteffenUrbaniak, AlicjaJurczyszyn, KamilUrbanska, Edyta MariaJurin, MislavUrbanucci, AlfonsoJurj, Ancuţa MariaUrbini, MilenaJurman, GiuseppeÜren, AykutJuszczyński, PrzemysławUreshino, HiroshiKa Fai, ToUrick, Mary EllenKaambre, TuuliUrra, Félix A.Kabała-Dzik, AgataUrsino, StefanoKachamakova-Trojanowska, NeliUshijima, KimioKadela-Tomanek, MonikaUshmorov, AlexeyKadenbach, BernhardUskoković, VukKadletz-Wanke, LorenzUssowicz, MarekKaesmann, LukasUsui, TatsuyaKagabu, MasahiroUsui, YoshihikoKagawa, TatehiroUtley, AdamKahlert, ChristophUversky, VladimirKahlert, UlfUyeda, TaroKahn, MichaelUygun, SahraKaido, ToshimiUyl-de Groot, Carin A.Kailayangiri, SareethaUysal-Onganer, PinarKaina, BerndUziel, OritKaipe, HelenUzunoglu, Faik G.Kaipparettu, Benny AbrahamV Ivanov, AlexanderKaira, KyoichiV. GRAHAM, SheilaKairemo, KaleviVacca, AngeloKairevičė, LauraVacher, PierreKaiser, JochenVachtenheim, JiriKaissis, GeorgiosVáclavíková, RadkaKajihara, MikioVagner, TatyanaKajtár, BélaVaidya, AmitaKakar, Sham SunderVaidya, AnandKakeji, YoshihiroVaidyanathan, GanesanKakizawa, NaoVaillant, FrancoisKakkassery, VinodhVaitkevičienė, GodaKakudo, KenichiValachis, AntonisKalamarides, MichelValdor, RutKalasekar, SharanyaValente, GuidoKalathiya, UmeshValentijn, L.J.Kale, AbhijitValentovic, MonicaKälin, Roland E.Valeria, RomeoKalita-de Croft, PriyakshiVali, RezaKállay, EniköValiante, SalvatoreKaller, MarkusValind, AndersKallergi, GalateaVallejo, AlejandroKallunki, TuulaVallera, DanielKalluru, PolirajuVallet, SoniaKalpadakis, ChristinaVallone, DanielaKaltsas, GregoryValmasoni, MicheleKaltschmidt, ChristianValtorta, SilviaKamada, YoshihiroVan Berge Henegouwen, Mark I.Kamath, DivyaVan Beusechem, VictorKambhampati, Siva PramodhVan Dam, PeterKameyama, KaoriVan De Stolpe, AnjaKamihira, MasamichiVan De Ven, RienekeKamijo, TakehikoVan De Vijver, KoenKamiya-Matsuoka, CarlosVan Den Berg, AnkeKamma, HiroshiVan Den Bergh, HubertKammala, Ananth KumarVan Den Brekel, Michiel Wilhelmus MariaKamphues, CarstenVan Den Eynden, JimmyKamposioras, KonstantinosVan Den Noortgate, WimKanai, TatsuakiVan Den Tempel, NathalieKanamori, TakaoVan der Burg, SjoerdKanayama, KojiVan Der Noort, VincentKanda, TatsuoVan Der Velden, Vincent H.J.Kanda, TeruVan Deurzen, Carolien H.M.Kandpal, ManojVan Eijck, Casper H.J.Kane, Patricia M.Van Es, Nick J.Kaneko, Syuzovan Geffen, Wouter H.Kanemitsu, YukihideVan Gent, DikKang, Dongchulvan Gerwen, Maaike A. G.Kang, EunjuVan Gompel, Jamie J.Kang, Hyun-CheolVan Gool, Stefaan W.Kang, Hyunseokvan Griethuysen, Joost J.M.Kang, JeonghyunVan Haele, MatthiasKang, JianmingVan Hal, GuidoKang, Koo JeongVan Kempen, LeonKang, Min KyuVan Leeuwen, Fijs W.B.Kang, StephenVan Leeuwen, Frank N.Kang, WeiVan Loveren, HenkKang, Yun PyoVan Luijk, PeterKanginakudru, SriramanaVan Marle, GuidoKannan, RaghuramanVan Meerbeeck, JanKanno, YuichiroVan Meeteren, Laurens A.Kant, ShivaVan Montfoort, NadineKantelhardt, Sven RainerVan Noorden, Cornelis Johannes ForrindinisKantyka, TomaszVan Overeem Hansen, ThomasKanugula, Anantha KoteswararaoVan Tienhoven, Geertjan J.Kao, Li-TingVan Tine, BrianKao, Shao-HsuanVan Tine, Brian AndrewKaplan, Michael J.Van Veldhoven, PaulKappos, Elisabeth A.Van Veldhuizen, PeterKapral, MałgorzataVan Vliet, Sandra J.Kar, AdwitiyaVan Waardenburg, RobertKar, AnirbanVan Werkhoven, ErikKarabon, LidiaVan Wyk, HesterKaragiannis, SophiaVan Zanten, MalouKarakaidos, PanagiotisVanan, Magimairajan IssaiKaram, SherifVandamme, TimonKaramouzis, MichalisVandenbroeck, KoenKaran, DevVanderLaan, Paul AKarantanos, TheodorosVandooren, JenniferKarayiannakis, AnastasiosVandyke, KateKardosh, AdelVankayalapati, HariprasadKaren, KeeshanVanneste, Ben G LKareta, Michael S.Vanni, DanieleKarmakar, ParthaVannini, EleonoraKarnoub, Antoine E.Vanpelt, JosKarp, JudithVanSaun, MichaelKarpel-Massler, GeorgVaquero, JavierKarpf, AdamVaradaraj, ArchanaKarras, PanagiotisVaradarajan, ShankarKarreman, Matthia A.Varas-Godoy, ManuelKarsak, MelihaVardaki, MarthaKarstedt, Silvia VonVarela, IgnacioKashaninejad, NavidVarela-Ramirez, ArmandoKashina, AnnaVarga, GeorgKashiwagi, ShinichiroVarkaris, AndreasKashofer, KarlVárkonyi, JuditKasi, Pashtoon MurtazaVarn, FrederickKasimir-Bauer, SabineVaron, ChristineKasinski, AndreaVarrault, AnnieKasman, LauraVartak, AbhishekKasparov, SergeyVasaikar, Suhas V.Kasper, BerndVashist, Yogesh KumarKaspi, EliseVassetzky, YegorKasprzak, AldonaVasseur, SophieKastan, MichaelVassilakopoulos, TheodorosKasten, Benjamin B.Vassiliadis, ThemistoklisKastritis, EfstathiosVassilopoulos, GeorgeKasza, AnetaVasuri, FrancescoKatabi, NoraVathipadiekal, VinodKataoka, NaoyukiVäyrynen, Juha P.Kataoka, Tatsuki RVazzana, MirellaKatapodi, Maria C.Vecchio, EleonoraKatarzyna, MalarzVeeramani, SureshKater, Arnon P.Veerapong, JulaKathawala, RishilVeeravalli, VijayabhaskarKato, KatsuhikoVega, Francisco M.Kato, MinoruVega-Rubín-De-Celis, SilviaKato, Takamitsu A.Vejux, AnneKato, YasumasaVel, Bharath KumarKatoh, HironoriVelasco, EladioKatsanis, EmmanuelVelasco, Gùillermo DanielKatsoris, PanagiotisVelasco-Hernández, TalíaKatsuta, ErikoVeldhuisen, Eran VanKaul, RashmiVeldkamp, ChristopherKauppi, LiisaVeleeparambil, ManojKauppila, Joonas H.Velez, ZéliaKaur, BalveenVelez-Cruz, RenierKaur, SandeepVelikova, TsvetelinaKaushik, Nagendra KumarVella, VeronicaKaushik, ShellyVellenga, EdoKawabata, HideakiVelleuer, EunikeKawakita, DaisukeVellone, Valerio GaetanoKawamura, TakahisaVelosa, TeresaKawamura, YusukeVeltkamp, ChristianKawano, KouichiroVelu, Sadanandan E.Kawano, YawaraVenderbos, LionneKawaoka, TomokazuVenerando, AndreaKawashima, MarikoVenkatadri, RajkumarKawauchi, KeikoVenkatesan, ThiagarajanKawiak, AnnaVenkateshaiah, Sathisha U.Kazdal, DanielVenner, Christopher PaulKe, YouqiangVenneti, SriramKeam, Simon P.Ventura, LuigiKędracka-Krok, SylwiaVenturelli, SaschaKeenan, JoanneVenturini, ElioKehoe, PatrickVenur, VyshakKei, Chan ChimVenuti, AldoKeisari, YonaVerdijk, Robert M.Kejík, ZdeněkVerdurmen, Wouter P.R.Kelleher, Fergal C.Vereb, GyörgyKeller, LauraVered, MarilenaKello, MartinVergani, FrancescoKelly, DervlaVergara, DanieleKelly, Gemma L.Verheij, JoanneKelly, KevinVerhelst, XavierKelts, JessicaVerheugd, PatriciaKemp, Michael G.Verhoef, LiaKemp, Zoe E.Verhoeyen, ElsKenawy, NihalVerlaan, Jorrit JanKendall, TimVerma, NupurKendel, FriederikeVerma, Shiv PrakashKennedy, LindseyVermeer, Paola D.Kennedy, MichaelVermijlen, DavidKennosuke, KarubeVernieri, ClaudioKenny, Hilary A.Veronese, AngeloKeramida, GeorgiaVeronesi, GiuliaKeresztes, AttilaVerrecchia, FranckKerkelä, ErjaVerrelle, PierreKerkhofs, MartijnVersino, ElisabettaKerl, KorneliusVersleijen-Jonkers, Yvonne M.H.Kern, PeterVersteeg, Anne L.Kerr, IanVerwaal, Victor JilbertKertmen, AhmetVerzoni, ElenaKesarwala, AparnaVesa, Stefan CristianKeshari, SunitaVesely, Matthew D.Kesharwani, Siddharth S.Veskoukis, Aristidis S.Kesterson, Joshua P.Vessoni, AlexandreKetteler, RobinVestal, Deborah J.Ketter, RalfVetrano, Ignazio GaspareKeung, EmilyVetsika, Eleni-KyriakiKhademi, AprilVetter, MarcusKhadka, Vedbar SinghViale, MaurizioKhakoo, ShelizeVicent Docón, María JesusKhaled, AnnetteVicentini, CaterinaKhalil, AndréVicier, CécileKhalilia, Walid MahmoudVickery, KarenKhammanivong, AliVideira, MafaldaKhan, HamidullahVideira, PaulaKhan, KrishnenduVider, JelenaKhan, Muhammad SaadVidili, GianpaoloKhan, NaeemViel, SébastienKhan, SabbirViganò, LucaKhan, SajidViganò, MauroKhan, ShafiqVíglaský, ViktorKhan, ShahanshahVigneri, RiccardoKhan, ShaheenVijay, Geraldine VidhyaKhan, SonjaVikas, PraveenKhanim, FarhatVilgelm, Anna E.Khare, TriptiVillabona, LisaKhatib, Abdel-MajidVillalonga, PriamKhella, HebaVillamizar, NestorKhleif, SamirVilla-Morales, MaríaKholová, IvanaVillani, RosannaKhong, TiffanyVillard, MarineKhoo, Bee LuanVillaverde, GonzaloKhoo, Ui-SoonVillegas, FernandaKhorram-Manesh, AmirVillegas-Sepulveda, NicolasKhoury, Joseph D.Vinall, RuthKhoury, ThaerVinante, LorenzoKhung, Yit LungVincent, AmyKhurana, NamrataVincenzi, BrunoKibriya, MuhammedVinciguerra, ManlioKidane, DawitViola, GiampietroKido, TatsuoVirag, LaszloKieber-Emmons, ThomasVirostko, JohnKiechle, MarionVirumbrales-Muñoz, MaríaKieda, ClaudineVisalli, Robert J.Kieffer, BrunoVisani, GiuseppeKikuchi, HidehikoVisconte, ValeriaKikuta, KazutakaVisconti, RobertaKikuyama, MasatakaVisentin, AndreaKil, Kun-EekVisser, LydiaKilic, EmineVitale, AlessandroKilvaer, Thomas KarstenVitale, ChiaraKim, Beom KyungVitale, MarioKim, Byung SooVitale, MassimoKim, Cheorl-HoVitale, Salvatore GiovanniKim, DonggeonVitiello, Pietro PaoloKim, DonginVito, AlyssaKim, EllaVitorino, Rui Miguel PinheiroKim, Euishin EdmundVittoria, RaffaKim, Grace Hyun J.Vittorio, OrazioKim, HaeryoungVivacqua, AdeleKim, Hun SikVivaldi, CaterinaKim, Hyo-CheolVivas Mejia, PabloKim, Hyoung-IlVizler, CsabaKim, JaehongVizoso-Vázquez, AngelKim, Jae-HoonVizovišek, MatejKim, Jae-hwanVlad, Anda MKim, Jin SuVlahopoulos, SpirosKim, Jin-WookVlahou, AntoniaKim, Jung HoVo, DatKim, Kristine M.Vodička, Pavel ErikKim, Kyeong KyuVodickova, LudmilaKim, Kyung EunVoena, ClaudiaKim, Min-HeeVoermans, CarlijnKim, Nam KyuVogt, Peter K.Kim, Pyung-HwanVoisin, Daniel L.Kim, Raymond H.Volante, MarcoKim, Ryong NamVolinia, StefanoKim, SangminVolk, Andrew G.Kim, Sang-WeVon Amsberg, GunhildKim, Seok JinVon Bubnhoff, NikolasKim, Seon-KyuVon Bueren, André O.Kim, Song CheolVon Der Thüsen, JanKim, Sung-HoonVoon, Dominic Chih ChengKim, Tae HyunVora, MehulKim, Tae IlVoso, Maria TeresaKim, Tae MinVoulgarelis, MichaelKim, Tae-DonVoutsinas, Gerassimos E.Kim, Tae-JungVrakas, GeorgiosKim, Won SeogVranic, SemirKim, WootaeVrieling, AlinaKim, Yeul HongVriend, JerryKim, Yong JunVučetić, MilicaKim, Yong-miVujanovic, NikolaKim, Young TaeVulpe, ChrisKim, Yu YeongVydra, NataliaKimer, NinaWach, SvenKimler, BruceWada, RyuichiKimura, KiminoriWaddell, NicolaKimura, MasahiroWade, Mark A.Kimura, ShojiWadham, CarolKimura, TakahiroWagner, AlainKing, Jonathan C.Wagner, Kay-DietrichKing, Peter H.Wagner, Kay-UweKinose, YasutoWagner, MarekKinugasa, HideakiWahl, Daniel R.Kirac, IvaWahli, WalterKirgan, Daniel M.Wakatsuki, MasaruKirschner, AustinWakimoto, HiroakiKiss, AndrásWałajtys-Rode, ElżbietaKiss, BernhardWalczyk, AgnieszkaKiss, CsongorWaldron, Richard T.Kitadate, AkihiroWaldum, HelgeKitagawa, SeiichiWalenkamp, AMEKitkit, WongWalerych, DawidKitt, JayWalkden-Brown, Stephen W.Kittakoop, PrasatWalker, BrianKittel, AgnesWalker, GraemeKiyohara, EijiWalker, LoganKiyokawa, EtsukoWalkley, CarlKjeldsen, EigilWallin, GöranKlampatsa, AsteroWalline, HeatherKlampfer, LidijaWallner, ChristophKlarenbeek, SjoerdWallwiener, MarkusKlassen, RolandWalpole, EuanKlebe, SonjaWalsh, Scott T.R.Klec, ChristianeWalters, ZoeKleeff, JörgWalton, JeffreyKlein, AndreasWaltz, SusanKlein, BrittaWan, ThomasKlein, StefanieWandosell, FranciscoKlener, PavelWang, BoKlepinin, AleksandrWang, Cheng-HsuKlijanienko, JerzyWang, Chia-SiuKlika, RiggsWang, Chia-YihKlimaszewska-Wiśniewska, AnnaWang, Chi-ChungKlinakis, ApostolosWang, Chih-hungKlinge, Carolyn M.Wang, ChihueiKlingemann, HansWang, ChunKlinkhammer-Schalke, MonikaWang, DaifengKlocker, HelmutWang, DejieKlose, JohannesWang, DennisKlughammer, JohannaWang, HaiKlümpen, Heinz JosefWang, HaifengKlussmann, Jens PeterWang, HeruiKlymenko, YuliyaWang, HsiuyingKnapp, StefanWang, HuaishanKnapper, StevenWang, Hui-minKnez, JureWang, Jaw-YuanKnight, KristinWang, JeffreyKnösel, ThomasWang, JunKnudsen, BeatriceWang, KaiKo, Jiunn-LiangWang, Kai-LeeKobayashi, HiroakiWang, KankanKobayashi, MakotoWang, KepengKobeissy, FirasWang, LishengKobiela, JarosławWang, LiyeKocbek, PetraWang, LizhongKoch, CameronWang, Peng-HuiKocoglu, MehmetWang, PingyuanKocot, EwaWang, Po-HuiKodama, TakahiroWang, QianbenKodet, OndrejWang, Qi-enKodidela, SunithaWang, RuizhongKoehl, Gudrun E.Wang, San MingKoekkoek, Johan A. F.Wang, ShidanKoelink, Pim J.Wang, ShikeKoff, Jean L.Wang, ShyiwuKofler, BarbaraWang, Tong-HongKoga, FumitakaWang, Tzu-FanKoga, TomoyukiWang, Wei-ShuKoga, YoshikatsuWang, WenqiKogawa, TakahiroWang, WentianKoh, DavidWang, XiaotangKoh, Kyung-NamWang, XiaoxingKoh, Mei YeeWang, Xin ShelleyKohaar, InduWang, XishaKohanbash, GaryWang, YaKoie, TakuyaWang, YihongKoivusalo, Antti IlmariWang, YoujinKoizume, ShiroWang, YulingKok, KlaasWang, ZhixiangKokabu, ShoichiroWängler, CarmenKolberg, Hans-ChristianWanibuchi, HidekiKolesar, Jill MarieWard, Stephen C.Kolettas, EvangelosWaring, PaulKoli, SwanandWarner, Blake M.Koliaraki, VasilikiWarner, EllenKolin, David L.Warnken, ZacharyKolisek, MartinWarta, RolfKoljonen, VirveWatabe, TadashiKolosnjaj-Tabi, JelenaWatanabe, EiichiKolostova, KatarinaWatanabe, HidetoKolot, MikhailWatanabe, TakashiKoltai, HinanitWatanabe, YoichiKomarowska, HannaWaterfall, Joshua J.Kommalapati, AnuhyaWaterhouse, MiguelKomohara, YoshihiroWaterton, JohnKomura, DaisukeWatson, WilliamKomuta, MinaWatson, ZacharyKonantz, MartinaWatts, Justin M.Kondo, TadashiWatzky, MurielleKondylis, EvangelisWdowiak, KamilKong, In DeokWeaver, JolantaKong, MoonkyooWeber, Georg F.Kong, Sun YoungWeber, K. ScottKönig, AlexanderWeber, RebekkaKonishi, HiroakiWebster, MarieKonishi, MasaakiWege, Anja KathrinKonner, Jason A.Weger, StefanKonstantakou, Eumorphia G.Weghorn, DonateKontny, UdoWegiel, BarbaraKontogianni, GeorgiaWegwitz, FlorianKontos, Christos K.Wehrhan, FalkKónya, JózsefWei, Hsiu-ChuanKoo, Bon SeokWei, Jun S.Koo, Kyo ChulWei, Wei-ChyouKoom, Woong SubWei, YonglongKoontz, Bridget F.Weidenhofer, JudithKopaczyńska, MartaWeidner-Glunde, MagdalenaKopanja, DraganaWeigand, IsabelKoper-Lenkiewicz, Olga MartynaWeihua, Zhang (John)Kopp, SaschaWeinfeld, MichaelKoprowski, PiotrWeinhold, NielsKorać, PetraWeis, EzekielKorade, ZeljkaWeis, SergeKorinek, VladimirWeisberg, EllenKorkaya, HasanWeiss, MartinKornek, MiroslawWelch, John S.Kornmann, MarkoWelin, StaffanKornprat, PeterWellner, Ulrich FriedrichKorolkova, Olga Y.Welsh, MichaelKorsching, EberhardWen, SijinKortüm, K. MartinWen, WeiKortylewski, MarcinWen, Yu-ChingKorytowski, WitoldWen-Chi, YangKosaka, YoshimasaWeng, Wen-KaiKosari, FarhadWenger, KatharinaKoschmieder, SteffenWennerberg, KristerKoscielny, SvenWerbowetski-Ogilvie, Tamra E.Koshiyama, MasafumiWerner, AndreasKosinsky, Robyn LauraWerner, JensKoskinas, JohnWery, MaximeKośla, KatarzynaWesche, JørgenKostelich, EricWest, NicholasKostopoulou, OuraniaWesterhout, Ellen M.Kosuge, YasuhiroWestermarck, JukkaKota, SatyaWesthoff, Mike-AndrewKotaška, KarelWesthrin, MaritaKotelevets, LarissaWhang, Young E.Kothari, VishalWhelan, Rebecca J.Kottaridi, ChristineWhipple, Chery A.Kotula, LeszekWhitby, DeniseKou, YiWhite, ChristineKoukourakis, Michael I.White, RobKoulouridis, StavrosWhitehall, VickiKounatidis, IliasWhitehurst, Christpher B.Koundourakis, PantelisWhiteside, ElizaKouokam, Joseph CalvinWhiteside, Theresa L.Kourtidis, AntonisWhitman, Eric DavidKoutelidakis, IoannisWhitman, GaryKovacic, HervéWhittaker, SeanKovács, AttilaWhittaker, StevenKoval, AlexeyWibowo, DavidKoval, OlgaWickström, MalinKovalenko, Elena I.Wiechec, EmiliaKovalszky, IlonaWieckowski, SébastienKővári, BenceWieczorek, EdytaKovbasnjuk, OlgaWiedenmann, BertramKowalczyk, Anne E.Wiegering, ArminKowalewska, MagdalenaWiegmans, Adrian P.Kowalik, ArturWiels, JoëlleKowalska, AldonaWiemer, Erik A.C.Kowalska, KarolinaWierman, Margaret E.Koya, RichardWieser, VerenaKoyama, ShoheiWiestler, BenediktKozaki, KoichiWiesweg, MarcelKozik, PatrycjaWietrzyk, JoannaKozłowska, JoannaWight, ThomasKrainer, Michael W.Wiita, Arun P.Krajewski, WojciechWijnhoven, Bas P.L.Krämer, Oliver H.Wilcox, Ryan AlanKranz, MathiasWilczek, BrigitteKrasniqi, AhmetWilder-Smith, PetraKratochwil, ClemensWilhelm, ThereseKrause, ElmarWilkens, LudwigKrause, GünterWilkins, SimonKrauthammer, MichaelWillenbacher, WolfgangKrawczyk, PrzemekWilley, Christopher D.Krebs, MarkusWilliams, Brent A.Kregel, StevenWilliams, BrianKreis, Nina-NaomiWilliams, Norman R.Kreis, StephanieWilliams, RichardKreissl, MichaelWilliams, RyanKrenács, TiborWilliams, Stephen J.Kretov, DmitryWilm, MatthiasKretzschmar, KaiWilmink, Johanna W.Krieg, AndreasWilson, EricaKrieg, SandroWilson, JustinKrishn, Shiv RamWilson, Philippe B.Krishna, Somashekar G.Wimmer, KatharinaKrishnan, PreethiWinchester, LauraKristensen, Lasse SommerWindhorst, SabineKristensen, MieWindle, Bradford E.Krizbai, IstvanWiner, Ira S.Krizbai, István A.Winer, JoshuaKroeze, Stephanie G.C.Winham, Stacey J.Kroiss, MatthiasWinqvist, OlaKrol, SilkeWinter, AlexanderKrstic, JelenaWinter, DominicKrug, SebastianWinter, GordonKruithof-de Julio, MariannaWinter, StefanKrum, Susan A.Winther-Larsen, AnneKrupar, RosemarieWirsching, Hans-GeorgKruspig, BjornWirsdörfer, FlorianKruyt, FrankWirth, ThomasKruyt, Frank A.e.Wischhusen, JörgKryeziu, KushtrimWise, Michael J.Kshirsagar, Prakash G.Wise-Draper, Trisha M.Książek, KrzysztofWitalisz-Siepracka, AgnieszkaKu, Cheol-ryongWitcher, MichaelKu, Hyo JeongWithrow, DianaKu, ZhiqiangWitjes, Max J.H.Kuban-Jankowska, AlicjaWittmann, JürgenKubiak, Jacek Z.Witz, IsaacKubik, MarkWitzel, IsabellKubo, NobuteruWitzens-Harig, MathiasKubo, YoshinaoWlodawer, AlexanderKucharczyk, MichaelWobus, ManjaKucinska, MalgorzataWohl, StefanieKudela, ErikWohlleber, DirkKudo, YasuseiWojda, UrszulaKueh, Hao YuanWojdacz, Tomasz K.Kuemmel, SherkoWolf, IdoKuhn, ElisabettaWölfler, AlbertKühn, ThorstenWollenberg, BarbaraKujan, OmarWon, MisunKukimoto, IwaoWong, AndreaKukuruzinska, Maria A.Wong, Chi-MingKulbacka, JulitaWong, Dennis C.C.Kulcenty, KatarzynaWong, Ee MingKulik, GeorgeWong, Ho LunKulka, JaninaWong, JudyKulkarni, PriyankaWong, Ka-LeungKulmacz, RichardWong, Kwan YeungKulus, MichałWong, Pamela T.Kumagai, YouichiWong, Richard W.Kumar Mishra, VivekWong, Stephen Q.Kumar, AshokWong, Yon-CheongKumar, NareshWoo, Ho-HyungKumar, PrashantWoo, ShiaoKumar, RajeshWood, Teresa L.Kumar, SunilWoodfield, SarahKumari, ShardaWoods, Nicholas T.Kumar-Sinha, ChandanWoodward, WendyKundrat, PavelWoodworth, Graeme F.Kung, Hsing-JienWorni, MathiasKung, Hsiu-NiWorst, Thomas StefanKung, SamWorth, Patrick J.Kunikowska, JolantaWozniak, Ann L.Kunkler, IanWozniak, MichalKunoh, TatsukiWoźniak-Budych, MartaKunz, MeikWren, DörteKuo, Chao-HungWrobel, Tomasz P.Kuo, Sung-HsinWu, (Jason) BoyangKuo, Wen-ChuanWu, CenKurata, MoritoWu, CheweiKure, ShokoWu, Chiao-EnKurita, HirofumiWu, Chin-ChungKuroi, YasuhiroWu, Chi-ReiKurozumi, SasaguWu, ChristinaKurrle, NinaWu, Chun-ChiKurth, InaWu, ChungChunKuryk, LukaszWu, Chung-ChunKusaczuk, MagdalenaWu, ErxiKushima, MikiWu, I-ChinKushner, Brian H.Wu, JenniferKuykendall, Andrew T.Wu, Jin-MingKveberg, LiseWu, JohnKveiborg, MarieWu, Kou-JueyKwack, KyuBumWu, MengKwak, Jong HwanWu, Meng-YuKwan, Hiu-yeeWu, Ming-HsunKwok, Henry Hang FaiWu, Min-HuanKwok, Hoi-HinWu, NanKwok, Wai-MengWu, Ting-FengKwok, Wing-Chi EdmundWu, Wen-ShengKwon, DeukwooWu, XianfangKwon, Seong YoungWu, YadiKwon, So HeeWu, YinghaoKwon, Tae-HyukWu, YoujunKwon, WoosungWu, Yuan HuaKwong, JosephWu, Yuh-LinKwun, Hyun JinWu, Yu-WeiKyo, SatoruWu, ZhaohuiKyprianou, NatashaWuescher, Leah M.Kypta, RobertWulf, Gerburg M.L. Royce, PeterWunderlich, MarkLa Forgia, DanieleWürth, RobertoLa Mendola, DiegoWust, PeterLa Monica, SilviaWyatt, HaleyLa Regina, GiuseppeXi, GuifaLa Starza, RobertaXi, YaguangLa Vecchia, CarloXia, BingLaakmann, ElenaXiao, JingjieLaas, EnoraXiao, ZhengtaoLaban, SimonXiao, ZhennaLabbé, SimonXie, HaiboLachowicz, Joanna IzabelaXie, JingwuLacina, LukasXie, YijingLacombe, JeromeXie, ZhiqunLadekarl, MortenXie, ZhongqiuLadomery, Michael R.Xie, Zhong-RuLaetsch, TheodoreXin, GangLafont, ElodieXin, YinLaFramboise, ThomasXing, FeiLagendijk, Anne KarineXiong, ShunbinLagopati, NefeliXiong, ZhaohuiLagundzin, DraganaXIRODIMAS, DmitrisLahlil, RachidXu, DaweiLahooti, HooshangXu, HongyanLai, CatherineXu, JieLai, Chian-HuiXu, RongLai, Jin-MeiXu, WenyanLai, KeaneXu, YanLai, QuirinoXu, YuexinLai, Wing-FuXu, Zhi PingLai, XianyinXu, ZhihongLai, XinXu, ZhijieLainas, PanagiotisXuan, ZhenyuLajolo, CarloXue, MingLakota, JanXue, XiangLakshmikuttyamma, AshakumaryXylinas, EvanguelosLalaoui, NajouaYabuki, KenichiroLallena, María JoséYadav, MarshleenLalli, EnzoYadav, SiddharthaLallous, NadaYaddanapudi, KavithaLam, Kim-HungYager, EricLam, MarnixYagishita, YokoLamango, Nazarius S.Yakirevich, EvgenyLamar, JohnYAMADA, HidetakaLamas-Maceiras, MónicaYamada, SuguruLamba, JatinderYamada, TaketoLambaudie, EricYamada, YoheiLambert, JoergYamakawa, DaishiLambrou, George IYamamichi, AkaneLamichhane, NarottamYamamoto, MasatoLamichhane, PurushottamYamamoto, MasayukiLamoureux, FrançoiseYamamoto, MizukiLampertico, PietroYamamoto, TakumiLamszus, KatrinYamamoto, YusukeLamy, Pierre-JeanYamamoto, YutakaLan, LanYamamura, SoichiroLan, Yuan-TzuYamanaka, RyuyaLandi, BrunoYamaoka, ToshimitsuLandon, Chelsea DawnYamasaki, TakahiroLandreville, SolangeYamashita, TatsuyaLandström, MareneYamauchi, AkiraLang, AlexanderYamauchi, HidekoLang, HaukeYamauchi, YoshioLang, LiweiYan, BowenLangdon, SimonYan, DayunLange, Carol A.YAN, LiangLanger, FlorianYan, ShengminLangerak, TonYan, XuefengLanikova, LucieYanagi, TerukiLannigan, DeborahYang, Chih-JenLannin, Donald R.Yang, Chih-YuLanza, EzioYang, ChunzhangLanzi, CinziaYang, Da-QingLanzillotta, MarcoYang, Eun JooLapalombella, RosaYang, FangfangLaPensee, ChristopherYang, HaifengLapidus, Rena S.Yang, Hung-ChiLapini, AlbertoYang, HushanLappano, RosamariaYang, JianLappin, TerryYang, Jiann-JouLapucci, AndreaYang, JiulingLardone, Patricia J.Yang, Ju DongLargaespada, David A.Yang, JunLarion, MioaraYang, Kuen-ChehLarionova, IrinaYang, Muh-HwaLarive, Romain MaximeYang, Pei-MingLarsen, Anders ChristianYang, QiyuanLascialfari, AlessandroYang, Seung-HoLasfar, AhmedYang, Sherry X.Lash, Lawrence H.Yang, Shun-FaLassalle, Henri-PierreYang, WancaiLastraioli, ElenaYang, Wei-hsiungLatenstein, CarmenYang, XiaoheLau, Allison N.Yang, XiaomingLaube, MarkusYang, Yea-Huei KaoLauby-Secretan, BéatriceYang, YoungLauder, BobYang, Zeng-QuanLaudisi, FedericaYang-Hartwich, YangLaura, EvangelistaYano, HajimeLaurent, GarderetYano, HirohitoLauricella, MariannaYano, ShuyaLaurino, SimonaYano, TomonoriLaursen, Kristian BruunYao, JianLauth, MatthiasYao, JunLautz, Timothy B.YAP, Celestial T.Lavarino, CinziaYashiro, MasakazuLavdaniti, MariaYasu, TakanoriLavrik, InnaYasuhara, TakaakiLaw, Man FaiYasumoto, MasatoLawler, JackYau, Siu-TungLawler, KatherineYazawa, TakashiLawrence, Theodore SYazdanifar, MaribelLayek, BuddhadevYe, Zhi-TingLayne, Tracy M.Yee, LisaLazar, CatalinYeh, Chun-NanLazarides, AlexanderYeh, I-JuLazennec, GwendalYeh, ShuyuanLe Dévédec, SylviaYeh, Ta-senLe Joncour, VadimYélamos, JoséLe Romancer, MurielYen, Meng-ChiLe Roy, ChristineYen, Yi-TingLe, MinhYeo, SynLea, SimonYeong, Joe Poh ShengLeal, Ana MendesYerushalmi, RinatLeask, AndrewYeung, Tsz-LunLebaron, SimonYi, Jin WookLechel, AndreYi, Joo MiLecureur, ValérieYi, Sun K.Lederer, AlbenaYi, TaoLederkremer, Gerardo Z.Yi, YongLee, A.-W.Yiallourou, AnnezaLee, Alvin J. X.Yilmaz, MusaLee, BelindaYimlamai, DeanLee, Byung IlYin, ChengqianLee, ByungHoonYin, LiLee, Chang HoonYing, HaoqiangLee, Chang LungYing, LeLee, Che-HsinYing, MingyaoLee, Cheng-I.Yip, Kevin Yuk-LapLee, Chia-HungYisraeli, JoelLee, Chia-HwaYlösmäki, ErkkoLee, Chien-HsingYlstra, BaukeLee, Dae HoYoda, SatoshiLee, Dean A.Yohe, MarielleLee, Dong HoYohe, Marielle E.Lee, Eun JungYokobori, TakehikoLee, Hee SeungYokoi, KenjiLee, Heung KyuYokota, HirokiLee, HoYokota, TakafumiLee, Hong-KiYokota, TomoyaLee, Hsin-ChenYokoyama, Kazunari K.Lee, Hsueh-TeYoneda, AkihiroLee, Huei-JaneYoneda, ToshiyukiLee, Hun JuYoneda, YukioLee, Ik JaeYoo, ChanghoonLee, Jae-HoYoo, Ji YoungLee, Jae-HyungYoo, So YoungLee, JandeeYoo, Tag KeunLee, Je-jungYoon, Hyeun JoongLee, Jenny H.J.Yoon, Karina J.Lee, Jeong-HoonYoon, Kyong-AhLee, Jeong-yeonYoon, Mee-SupLee, Ji MinYoon, Sung-SooLee, JieYoon, Won-SupLee, Jih-ChinYordanov, AngelLee, JiyunYoshida, Go J.Lee, JongbumYoshida, JunjiLee, Jong-SooYoshida, KatsunoriLee, Jong-wooYoshimura, YuichiLee, Joo HyoungYoshino, HironoriLee, Joo YongYoshioka, Ken-ichiLee, JungwooYoshiura, TakashiLee, Jung-YunYoshiyama, HironoriLee, Ki YoungYoshizaki, TomokazuLee, Kin Wah TerenceYou, JianxinLee, KitfaiYou, LiangLee, Kyu EunYou, Ren-InLee, MariaYou, XiaonaLee, MinaYoun, Hyun JoLee, Nita KarnikYoung, ArabellaLee, Sei YoungYoung, Jason C.Lee, Seung EunYousefi, BardiaLee, Shao-ChenYousefi, Behrooz HooshyarLee, Shih-yuYrlid, UlfLee, StephenYsebaert, LoicLee, SukmookYu, Cheng-PingLee, Tae JinYu, ChunsongLee, Tae SupYu, JeongsikLee, Tsung-HsienYu, Jian Q.LEE, Victor Ho-funYu, JinxingLee, Wai-LengYu, RunLee, Wei-HwaYu, StanleyLee, Wei-JiunnYu, TengtengLee, YaelimYu, WeimiaoLee, Yen ChienYu, Willie Shun ShingLee, Yi-JangYu, XinhuaLee, Young JinYu, XiupingLee, ZhenghongYu, YanpingLeenders, William P.J.Yu, YuLeffler, HakonYu, Yung-LuenLefort, SylvainYuan, JupingLeggett, BarbaraYuan, Shyng Shiou F.Legler, Daniel F.Yuan, XuegangLehen’kyi, V’yacheslavYuan, YanLehner, ManfredYuan, ZhihongLehner, RichardYue, JunmingLehnhardt, MarcusYue, XuyiLei, WeiYuen, Karen Wing YeeLei, Yu L.Yuh, Chiou-HwaLeibowitz-Amit, RayaYun, Chae-OkLeich, EllenYun, Sook JungLeichtle, Alexander BenediktYung, Hong-WaLeiphrakpam, PremilaYupo, MaLejeune, FabriceYura, YoshiakiLejman, MonikaYusuf, NabihaLejniece, SandraYuzhalin, ArseniyLele, ShashikantYuzhalin, Arseniy E.Leloup, LudovicYves, CombarnousLemay, GuyZaballos, MiguelLemieux, KiTani ParkerZabielska-Koczywąs, KatarzynaLemmon, ChristopherZaboronok, AlexanderLemonnier, LoicZaccara, SaraLendvai, GáborZadik, YehudaLeng, RogerZaenker, KurtLenzi, MoniaZaffaroni, NadiaLenzi, PaolaZage, Peter E.Leon Mateos, LuisZagorac, SladjanaLeon Serrano, JavierZaharoff, DavidLeonardo, CostantinoZahrieh, DavidLeoncini, LorenzoZaidi, SaheemLeone, PatriziaZaika, AlexanderLeonetti, FrancescoZaiss, DietmarLeong, Hon SingZajączkowska, RenataLeon-González, Antonio J.Zakany, RozaLepage, CecileZakaria, RasheedLe-Petross, Huong CarisaZakrocka, IzabelaLeporatti, StefanoZakrzewska, MagdalenaLeppert, JohnZalatnai, AttilaLe-Rademacher, Jennifer G.Zalewska, AnnaLerario, Antonio M.Zamarchi, RitaLerchen, Hans-GeorgZambelli, AlbertoLerebours, FlorenceZambello, RenatoLesch, BlumaZamboglou, ConstantinosLeser, UlfZamò, AlbertoLesina, MarinaZampini, MatteoLesinski, GregoryZan, HongLeslie, KimberlyZanagnolo, VannaLesniak, WieslawaZanetti, MaurizioLeso, VerusckaZangemeister-Wittke, UweLeszczynska, KatarzynaZani, Bianca MariaLetellier, ElisabethZaniboni, AlbertoLethaus, BerndZannoni, Gian FrancoLeuci, ValeriaZapico, Sara CasadoLeung, DilysZara, FrancescaLeung, Elaine Y.L.Zaravinos, ApostolosLev, SimaZarnegar, RasaLevantini, ElenaZarobkiewicz, MichałLevi Sandri, Giovanni BattistaZarrinpar, AliLévi, FrancisZatelli, Maria ChiaraLevine, Edward A.Zattoni, FabioLevine, HerbertZaucha, JanLevi-Polyachenko, NicoleZauderer, Marjorie G.Levonen, Anna-liisaZavattaro, ElisaLewicki, SławomirZavyalova, Elena G.Lewin, JeremyZawacka-Pankau, JoannaLewis Phillips, Gail D.Ždralević, MašaLewis, AnnabelleZdrowowicz, MagdalenaLewis, Stephen M.Zdzisińska, BarbaraLeyton, Jeffrey V.Zebisch, ArminLezot, FredericZedenius, JanLheureux, StephanieZehentmayr, FranzLi, BingZeidán-Chuliá, FaresLi, ChenghuiZeinali, MinaLi, ChenyuZeinomar, NurLi, Chia-YangZelenka, JaroslavLi, Chien-ChunZelensky, Alex N.Li, Chien-FengZeleznik, Oana AlinaLi, ChunyingZelionka, JacekLi, DeminZemmour, DavidLi, FuyangZeng, BijunLi, HaochengZeni, OlgaLi, HaoxunZerdes, IoannisLi, HeZhai, KuiLi, Heng-HongZhan, FenghuangLi, HuaZhan, YuLi, HuiZhang, ChengchengLi, HuinanZHANG, Daniel XinLi, JianjianZhang, DianbaoLi, JineZhang, DongLi, JoanZhang, Donna D.Li, LiZhang, DuoLi, LinZhang, ErshuaiLi, LingZhang, FanLi, LingxiaoZhang, HongLi, MinZhang, HuaLi, MinglunZhang, Jian-TingLi, RonghuiZhang, JiluLi, Ruei-NianZhang, JiwangLi, ShantaoZhang, LingLi, Shau-HsuanZhang, NuLi, Shengwen CalvinZhang, QiangLi, Shuai ChengZhang, QingLi, TianhuZhang, QiuyangLi, WeihanZhang, RuiLi, Xiang-GuoZhang, ShuLi, XiaohongZhang, SicongLi, Xiao-nanZhang, WeijieLi, XiaotaoZhang, WeizhouLi, XingZhang, WencaiLi, YanZhang, XiaoliLi, YongZhang, XiaonanLi, YulinZhang, XiaowenLi, YunfeiZhang, XiaoyuLi, YvonneZhang, XinminLi, ZuofengZhang, XinnaLiagre, BertrandZhang, XiyuanLiakina, ValentinaZhang, YanbinLian, IanZhang, YaqingLiang, FrankZhang, YeLiang, Jessie QiaoyiZhang, YifanLiang, JunZhang, YongliangLiang, ShuangZhang, YueLianidou, EviZhang, YumiaoLianos, Georgios D.Zhang, YuxiaLiao, Ai-HoZhang, ZhaoLiao, Joseph C.Zhang, ZhibingLiao, Jyh-feiZhang, ZhiguoLiao, PengZhao, ChunxiaLiao, Yi-JenZhao, GexinLiao, Yung-FengZhao, HongLiberi, GiordanoZhao, QianLibra, MassimoZhao, QingxiaLichner, ZsuzsannaZhao, WeixingLicht, Jonathan D.Zhao, WenxiuLicht, ThomasZhao, XiangLieberman, Paul M.Zhao, YuLien, Ming-YuZhao, YueLiesveld, JaneZhao, YunfengLieto, EvaZhao, ZiboLikar, RudolfZhen, DavidLiktor-Busa, ErikaZheng, DandanLillard, JamesZheng, SiyuanLiloglou, LakisZheng, Yun-WenLim, BoraZhong, Haizhen AndrewLim, Chwee MingZhong, JimLim, Dong JunZhou, BinhuaLim, EstherZhou, DawangLim, Hyunjung JadeZhou, GangLim, Megan S.Zhou, Heshan SamLim, Sang MooZhou, JianLim, Sung-JigZhou, JingyingLim, WhasunZhou, LinliLimagne, EmericZhou, QiminLin, AbrahamZhou, QingyuLin, Been-RenZhou, ShengmeiLin, Chang-ShenZhou, Tianhao (Vincent)Lin, Chien-MinZhou, WeijieLin, ChingyuZhou, WenLin, Chun-YenZhou, WenchaoLin, DechenZhou, Yi-hongLin, GiginZhou, ZhijunLin, HoZhou, ZhixiongLin, Huang-YunZhu, ChrisLin, JunZhu, JunLin, Kuo-ShyanZhu, XunjinLin, Liang-InZhu, YuLin, Liang-tingZhu, ZheLin, LiyongZhu, ZiwenLin, Mei-HsiangZhuang, XiaoxuanLin, Muh-ShiZhuang, ZhengpingLin, Pao-HwaZhurakivska, KhrystynaLin, Pei-HuiZi, XiaolinLin, Peter P.Zielinski, RafalLin, RubyZigrino, PaolaLin, Shian-RenZimmer, JacquesLin, Shi-MingZimpfer, AnnetteLin, Shu-ChunZimta, Alina-AndreeaLin, Su-FangZińczuk, JustynaLin, TaoZindler, Jaap DoekeLin, Tzer-BinZingone, FabianaLin, WeifengZiranu, PinaLin, WenshengZmarzły, NikolaLin, XianzhiZoccali, GiovanniLin, YanZong, Wei-XingLin, YanghsiangZoratti, MarioLin, YaoZoroddu, Maria AntoniettaLin, YibinZorzi, ManuelLin, Yi-TingZou, ChunbinLin, Yuh-YihZou, Wen-QuanLin, ZhenZou, YueLin, ZhoumengZschäbitz, StefanieLinardic, Corinne M.Zschaeck, SebastianLind, Michael J.Zsiros, EmeseLindahl, Lise M.Zsíros, JõzsefLindenmann, JoergZubair, HaseebLinder, BenediktZugaza, José LuisLinder, StigZugazagoitia, JonLindner, Holger A.Zur Hausen, AxelLindner, LarsZwahlen, DanielLindner, RobertZwergel, ClemensLindquist, Jonathan A.Zylicz, Maciej

